# Association between ADHD and vision problems. A systematic review and meta-analysis

**DOI:** 10.1038/s41380-022-01699-0

**Published:** 2022-08-05

**Authors:** Alessio Bellato, John Perna, Preethi S. Ganapathy, Marco Solmi, Andrea Zampieri, Samuele Cortese, Stephen V. Faraone

**Affiliations:** 1 School of Psychology, University of Nottingham Malaysia, Semenyih, Malaysia; 2 Institute of Psychiatry, Psychology and Neuroscience, King’s College London, London, UK; 3 Department of Psychiatry and Behavioral Sciences, SUNY Upstate Medical University, Syracuse, NY USA; 4 Department of Psychiatry and Behavioral Sciences, Emory University School of Medicine, Atlanta, GA USA; 5 Department of Ophthalmology & Visual Sciences, SUNY Upstate Medical University, Syracuse, NY USA; 6 Department of Psychiatry, University of Ottawa, Ottawa, ON Canada; 7 Department of Mental Health, The Ottawa Hospital, Ottawa, ON Canada; 8 Ottawa Hospital Research Institute (OHRI) Clinical Epidemiology Program, University of Ottawa, Ottawa, ON Canada; 9 School of Epidemiology and Public Health, Faculty of Medicine, University of Ottawa, Ottawa, ON Canada; 10 Centre for Innovation in Mental Health, School of Psychology, Faculty of Environmental and Life Sciences, University of Southampton, Southampton, UK; 11 Department of Child and Adolescent Psychiatry, Charité Universitätsmedizin, Berlin, Germany; 12 Vittorio Emanuele III Hospital, Montecchio Maggiore, Vicenza, Italy; 13 Solent NHS Trust, Southampton, UK; 14 Clinical and Experimental Sciences (CNS and Psychiatry), Faculty of Medicine, University of Southampton, Southampton, UK; 15 Hassenfeld Children’s Hospital at NYU Langone, New York University Child Study Center, New York, NY USA; 16 Division of Psychiatry and Applied Psychology, School of Medicine, University of Nottingham, Nottingham, UK

**Keywords:** Neuroscience, Molecular biology, ADHD

## Abstract

**Aim:**

To conduct the first systematic review and meta-analysis assessing whether attention-deficit/hyperactivity disorder (ADHD) is associated with disorders of the eye, and/or altered measures of visual function.

**Method:**

Based on a pre-registered protocol (PROSPERO: CRD42021256352), we searched PubMed, Web of Knowledge/Science, Ovid Medline, Embase and APA PsycINFO up to 16th November 2021, with no language/type of document restrictions. We included observational studies reporting at least one measure of vision in people of any age meeting DSM/ICD criteria for ADHD and in people without ADHD; or the prevalence of ADHD in people with and without vision disorders. Study quality was assessed with the Appraisal tool for Cross-Sectional Studies (AXIS). Random effects meta-analyses were used for data synthesis.

**Results:**

We included 42 studies in the narrative synthesis and 35 studies in the meta-analyses (3,250,905 participants). We found meta-analytic evidence of increased risk of astigmatism (OR = 1.79 [CI: 1.50, 2.14]), hyperopia and hypermetropia (OR = 1.79 [CI: 1.66, 1.94]), strabismus (OR = 1.93 [CI: 1.75, 2.12]), unspecified vision problems (OR = 1.94 [CI: 1.38, 2.73]) and reduced near point of convergence (OR = 5.02 [CI: 1.78, 14.11]); increased lag (Hedge’s *g* = 0.63 [CI: 0.30, 0.96]) and variability (Hedge’s *g* = 0.40 [CI: 0.17, 0.64]) of the accommodative response; and increased self-reported vision problems (Hedge’s *g* = 0.63 [CI: 0.44, 0.82]) in people with ADHD compared to those without ADHD (with no significant heterogeneity). We also found meta-analytic evidence of no differences between people with and without ADHD on retinal nerve fiber layer thickness (Hedge’s *g* = −0.19 [CI: −0.41, 0.02]) and refractive error (Hedge’s *g* = 0.08 [CI: −0.26, 0.42]) (with no significant heterogeneity).

**Discussion:**

ADHD is associated with some self-reported and objectively ascertained functional vision problems, but not with structural alterations of the eye. Further studies should clarify the causal relationship, if any, between ADHD and problems of vision.

**Trial registration:**

PROSPERO registration: CRD42021256352.

## Introduction

Attention-deficit hyperactivity disorder (ADHD) is a neurodevelopmental disorder affecting 5% of children and 3% of adults and characterized by developmentally inappropriate inattention and/or hyperactivity/impulsiveness [1–3]. ADHD is associated with negative life outcomes such as low academic and professional achievements, increased risk of substance use and psychiatric comorbidities [1].

Considering that vision problems, including vision loss, blurred vision and strabismus, are influenced by environmental biological factors (e.g., pre-term birth [4] and/or systemic infections such as toxoplasmosis) [5, 6], and given the established evidence on the involvement of these environmental risk factors in ADHD etiology [7], altered neurodevelopment may concurrently lead to the onset of vision disorders and symptoms of ADHD. Moreover, structures of the eye develop from the same embryological tissue as the brain [7, 8] and ADHD is a neurodevelopmental disorder presenting with structural brain abnormalities [9]. Therefore, the development of ocular structures (including major structures of the eye and neural connections with brain structures involved in visual information processing and perception) might be affected by the same processes that cause ADHD [7, 8, 10]. Although the exact etiology of ADHD is not well understood [11, 12], the mechanisms of first line medications to treat ADHD, in conjunction with molecular and neuroimaging studies, implicate serotonergic, noradrenergic and dopaminergic neural pathways in its pathophysiology [13–15]. Of note, dopaminergic neurons in the retina play a key role in color perception, contrast sensitivity, light adaptation, and spatial and temporal processing [16, 17], and altered dopamine transmission characterizes ADHD (but also neurological and neurodegenerative conditions [18]). Therefore, we hypothesized the presence of an association between ADHD and alterations in functional or perceptual domains of vision (e.g., color vision and contrast sensitivity).

There is meta-analytic evidence of structural abnormalities of the eye itself in ADHD, together with altered oculomotor control. Li et al. [19] found reduced retinal nerve fiber layer (RNFL) in people with ADHD; however, their systematic search resulted in only four eligible studies to be included in the meta-analyses. Maron et al. [20] recently confirmed the presence of oculomotor disturbances in people with ADHD compared to neurotypical individuals, especially for saccade inhibition and control, and visuo-spatial memory; considering that their systematic review and meta-analysis was published in 2021, we decided to not include, in our study, any study investigating eye movements in ADHD. There is also evidence of increased prevalence of vision problems [21–23] and disorders of vision such as strabismus [24–26], hyperopia [24, 27, 28], astigmatism [25, 26, 28], altered contrast sensitivity [29, 30] and color vision [31–36] in ADHD. However, as of today, no meta-analysis has been conducted to systematically investigate the prevalence of vision disorders in ADHD, or whether ADHD is more prevalent in people with vision problems.

To sum up, the current study aimed to: (a) investigate if the prevalence of ADHD differed in people with and without vision problem and, vice versa, if the prevalence of vision problems/conditions differed in people with and without ADHD; and (b) investigate differences in objective measures of vision (e.g., structural ocular measures, visual acuity, contrast sensitivity and color vision) between people with and without ADHD.

## Methods

This systematic review and meta-analysis followed the 2020 PRISMA (Preferred Reporting Items for Systematic Reviews and Meta-Analyses) guidelines [37]. The PRISMA Checklist is reported in Supplementary 1 and the protocol was pre-registered on PROSPERO (CRD42021256352).

### Search strategy

We systematically searched Pubmed, Web of Knowledge/Science, Ovid Medline, Embase and APA PsycInfo until 16th November 2021, with no language/type of document limits. The search strategy/syntax included keywords associated with (a) ADHD and (b) vision (additional details in Supplementary 2).

### Selection criteria

Studies were considered eligible for inclusion if they met the following criteria: (1) original, observational studies (case studies and previous systematic or narrative reviews were not included, but reference lists were searched to identify any additional eligible studies); (2) including people of any age meeting DSM III, III-R, IV (TR), 5 or ICD 9–10 diagnostic criteria for ADHD; and (3) comparing at least one measure of vision in people with vs. those without ADHD. Moreover, we also included studies reporting the prevalence of ADHD in people with and without any disorders of vision.

### Data selection, extraction, and coding

Titles and abstracts of studies retrieved from the searches were screened independently by two authors (AB and JP) to identify those that potentially met inclusion criteria; disagreements were resolved through discussion. The full text of each article marked as eligible for inclusion was assessed for final inclusion. Data were extracted from eligible studies using standardized forms by two authors (AB and JP). Extracted information included: study design, sample characteristics (size, age, sex, socio-demographic background), clinical characteristics (ascertainment of clinical diagnosis, presence of co-occurring conditions), outcome measures (type of measure, unit measure, method and tool utilized, mean and standard deviation, SD). Data not available from publications were systematically requested from corresponding, first or senior authors via e-mail. Publications for which data were initially not available and were received by the authors are indicated in Table 1.

**Table 1:** Characteristics of studies investigating vision in ADHD.

Study, ref.	Sample size	Developmental stage	Measure(s)	Main findings
Ababneh et al. [27]	55 ADHD; 55 no-ADHD	Children and adolescents	Macular thickness; myopia, hyperopia and hypermetropia; near point convergence; corneal topography; cycloplegic refraction; visual acuity	No differences in tomography between ADHD and no-ADHD. Abnormal NPC in 41.9% of children with ADHD.
Akmatov et al. [51]	258,662 ADHD; 2,327,958 no-ADHD	Children and adolescents	Unspecific eye problems	Unspecific eye problems were found in 16% of patients with ADHD vs. 8.6% of those without.
Aslan et al. [61]	32 ADHD; 43 no-ADHD	Children and adolescents	Macular thickness and volume; retinal nerve fiber layer thickness	No statistically significant differences between ADHD and no-ADHD.
Ayyildiz and Ayyildiz [54]	30 ADHD; 30 no-ADHD	Children and adolescents	Axial length (of the eye); corneal curvature radius, diameter and thickness; macular thickness; retinal nerve fiber layer thickness	Corneal thickness and axial length were significantly higher in ADHD, while corneal curvature radius was significantly lower. No significant difference on RNFL thickness, macular thickness, corneal diameter and anterior chamber depth measurements.
Bae et al. [59]	12 ADHD; 13 no-ADHD	Children and adolescents	Intraocular pressure; macular thickness; refractive error	Increased macular thicknesses in ADHD.
Banaschewski et al. [31]	14 ADHD; 13 no-ADHD	Children and adolescents	Color discrimination	Increased difficulties in color discrimination in ADHD, especially along the blue–yellow axis.
Bartgis et al. [67]	54 ADHD; 56 no-ADHD	Children and adolescents	Contrast sensitivity	Reduced contrast sensitivity in patients with ADHD-Combined vs. no-ADHD (no differences between ADHD-inattentive and no-ADHD).
Berger et al. [21]	7584 ADHD; 298,380 no-ADHD	Young adults	ADHD in people with color vision deficiency	ADHD was reported in 0.36% of patients with color vision deficiency and 0.03% of those without.
Bodur et al. [55]	31 ADHD; 31 no-ADHD	Children and adolescents	Ganglion cell layer thickness; optic nerve thickness; retinal nerve fiber layer thickness	Reduced GCL thickness in ADHD.
Brown et al. [68]	12 ADHD; 12 no-ADHD	Children and adolescents	Flicker fusion threshold at high and low contrast	No statistically significant differences between ADHD and no-ADHD.
Bubl et al. [65]	20 ADHD; 20 no-ADHD	Adults	Contrast gain	No statistically significant differences between ADHD and no-ADHD.
Bubl et al. [64]	20 ADHD; 20 no-ADHD	Adults	Retinal background noise	Increased retinal background noise in adults with ADHD compared to no-ADHD.
DeCarlo et al. [72]	56 ADHD; 189 no-ADHD	Children and adolescents	Visual acuity	ADHD was found associated with less risk of having nystagmus and having worse visual acuity.
DeCarlo et al. [22]	1,017 ADHD; 74,073 no-ADHD	Children and adolescents	ADHD in people with vision problems	ADHD was more prevalent among children with vision problems vs. normal vision.
Donmez et al. [29]	30 ADHD; 30 no-ADHD	Children and adolescents	Contrast sensitivity	Reduced contrast sensitivity in ADHD.
Fabian et al. [53]	56 ADHD; 66 no-ADHD	Children and adolescents	Visual acuity; amplitude of accommodation; convergence insufficiency; fusional amplitude: heterophoria: near point convergence: refraction: stereoacuity	Reduced NPC in ADHD.
Farrar et al. [74]	24 ADHD; 19 no-ADHD	Children and adolescents	Visual symptoms	Increased self-reported visual symptoms in ADHD.
Grönlund et al. [24]	42 ADHD; 50 no-ADHD	Children and adolescents	Axial length (of the eye); inner canthial distance; refractive error; anisometropia; astigmatism; heterophoria; visual acuity; hyperopia/hypermetropia; myopia; near point convergence; stereoacuity; vision problems; neuroretinal rim; optic cup and disc; index of tortuosity for veins of the optic fundus;	Subtle morphological changes of the optic nerve and retinal vasculature were found in ADHD.
Guvenmez et al. [60]	40 ADHD; 36 no-ADHD	Children and adolescents	Intraocular pressure	No statistically significant differences between ADHD and no-ADHD.
Hergüner et al. [62]	45 ADHD; 45 no-ADHD	Children and adolescents	Macular thickness and volume; retinal nerve fiber layer thickness	Lower RNFL thickness in ADHD, in nasal quadrant.
Ho et al. [25]	116,308 ADHD; 116,308 no-ADHD	Children and adolescents	Amblyopia; astigmatism; heterotropia; hyperopia and hypermetropia	Higher prevalence of amblyopia, hypermetropia, astigmatism and heterotropia in ADHD.
Işik and Kaygisiz [56]	58 ADHD; 44 no-ADHD	Children and adolescents	Ganglion cell layer thickness; intraocular pressure; macular thickness; retinal nerve fiber layer thickness	No significant differences in IOP, global RNFL thickness, central macular thickness, and GCL thickness.
Karaca et al. [28]	23 ADHD; 48 no-ADHD	Children and adolescents	Astigmatism; hyperopia/hypermetropia; myopia; convergence and divergence angle; stereoacuity	Lower stereoacuity in ADHD.
Kim et al. [32]	30 ADHD; 30 no-ADHD	Adults	Color discrimination; contrast sensitivity; Visual symptoms	Increased difficulties in discriminating blue and red, in females with ADHD vs. no-ADHD. No differences in contrast sensitivity.
Kim et al. [33]	30 ADHD; 30 no-ADHD	Adults	Color discrimination	Increased difficulties in discriminating blue and red, in females with ADHD vs. no-ADHD. No differences in contrast sensitivity.
Kim et al. [35]	30 ADHD; 30 no-ADHD	Adults	Color discrimination; visual symptoms	Increased self-reported visual symptoms in ADHD.
Kim et al. [34]	16 ADHD; 15 no-ADHD	Children and adolescents	Spherical correction; color blindness; visual acuity	No significant differences in ophthalmological measures or color discrimination.
Kooij and Bijlenga [52]	149 ADHD; 263 no-ADHD	Adults	Visual symptoms (photophobia)	Increased self-reported visual symptoms in ADHD.
Martin et al. [73]	18 ADHD; 24 no-ADHD	Children and adolescents	Refractive error; visual acuity	No differences in visual acuity or refractive errors in ADHD, but improvement with medication.
McBride and Bijan [50]^a^	95677 (ADHD and no-ADHD)	Children and adolescents	ADHD in people with vision problems	Increased prevalence of ADHD in people with vision problems.
Merdler et al. [48]	1598 ADHD; 661,043 no-ADHD	Children and adolescents	ADHD in people with strabismus	Increased prevalence of ADHD in people with corrected strabismus.
Mohney et al. [49]	407 ADHD; 407 no-ADHD	Children and adolescents	ADHD in people with strabismus	Increased prevalence of ADHD in people with exotropia.
Redondo et al. [69]	18 ADHD; 18 no-ADHD	Children and adolescents	Accommodation; visual symptoms; refractive error; visual acuity	Increased lags and variability of accommodation in ADHD.
Redondo et al. [70]	23 ADHD; 31 no-ADHD	Children and adolescents	Accommodation; refractive error	Increased lags of accommodation in ADHD.
Redondo et al. [71]	22 ADHD; 22 no-ADHD	Children and adolescents	Accommodation	Increased lags of accommodation in ADHD.
Reimelt et al. [26]	660 ADHD; 12,828 no-ADHD	Children and adolescents	Astigmatism; hyperopia; myopia; strabismus	Increased prevalence of hyperopia, astigmatism, and strabismus (but not myopia) in ADHD.
Roessner et al. [66]	14 ADHD; 14 no-ADHD	Children and adolescents	Color discrimination	Increased difficulties in color discrimination in ADHD, especially along the blue–yellow axis.
Sánchez-Guillén et al. [57]	23 ADHD; 23 no-ADHD	Children and adolescents	Macular thickness; retinal nerve fiber layer thickness; refractive error; visual acuity	Lower central macular thickness in ADHD, no differences in GCC or RNFL.
Su et al. [23]	6817 ADHD; 27,268 no-ADHD	Children and adolescents	ADHD in children with amblyopia	Increased prevalence of ADHD in people with amblyopia.
Tunel and Keskek [58]	26 ADHD; 26 no-ADHD	Adults	Macular thickness; retinal nerve fiber layer thickness; ganglion cell layer thickness	Lower central macular thickness, RNFL thickness, ganglion cell layer thickness, inner macular ring outer macular ring thicknesses in ADHD.
Uebel-von Sanderslebenet al. [36]	14 ADHD; 15 no-ADHD	Children and adolescents	Color discrimination	Increased difficulties in color discrimination in ADHD, especially along the blue–yellow axis.
Ulucan Ataset al. [30]	37 ADHD; 37 no-ADHD	Children and adolescents	Contrast sensitivity; macular thickness; retinal nerve fiber layer thickness; ganglion cell complex thickness	Lower contrast sensitivity and RNFL thickness in ADHD.
Werner et al. [63]	20 ADHD; 21 no-ADHD	Adults	Retinal background noise	Increased retinal background noise in ADHD.

^a^Data not available in the original paper, gathered via e-mail from the authors.

### Outcomes and assessment of study quality

For studies that reported mean and SD of any outcome measure within the scope of the review, in people with and without ADHD, the standardized mean difference (Hedge’s g) and its variance were calculated [38]. The natural logarithm of the odds ratio (LogOR) and its variance were calculated for studies that reported the number of people with and without a certain vision disorder/problem in people with ADHD and without ADHD, or the prevalence of ADHD (e.g., number of patients) in people with and without vision problems/disorders [38]. Study quality was rated by two authors (AB and JP) with the Appraisal tool for Cross-Sectional Studies (AXIS [39]) (see Supplementary 5).

### Data synthesis and analysis

A narrative synthesis was performed for all studies included in the systematic review. Meta-analyses were conducted in *R 4.1.0* [40] to estimate the pooled effect size across studies for each outcome, whenever at least two studies reporting on the same outcome were available. Random effects meta-analytic models were fitted to the data in *metafor* [41] with effect sizes nested within studies for those that reported multiple effect sizes for the same component to account for non-independence of data (multivariate models). The Restricted Maximum-Likelihood (REML) estimator was used with Knapp-Hartung confidence interval adjustment [42]. The Cochran’s Q test was used to investigate the presence of significant heterogeneity [43]. Publication (small study) bias was assessed visually using funnel plots and quantitatively with the Egger’s test [44], whenever at least 10 effect sizes were included in the meta-analysis, as suggested by Borestein et al. [45]. For multivariate meta-analytic models, the rank correlation test for funnel plot asymmetry was used [46]. Trim and fill analyses were performed to identify any potentially missing studies in funnel plots, due to publication bias [47]. Supplementary 3 reports the amendments to the original protocol, with reasons for the changes.

## Results

**Fig. 1:**
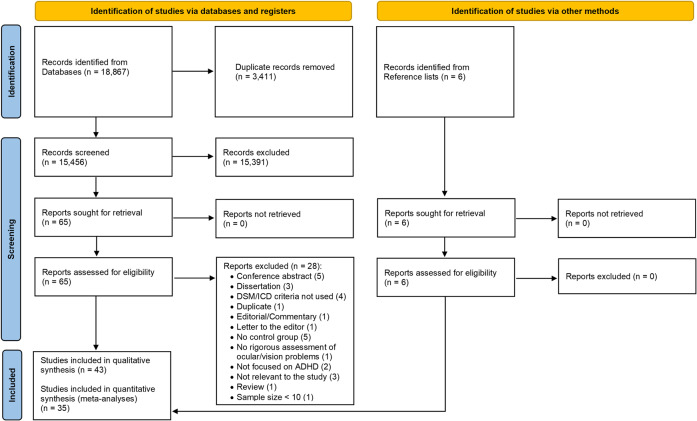
PRISMA flowchart. Graphical representation of number of papers retrieved, screened and included in the narrative reviews and meta-analyses. From: Page et al. [37].

**Fig. 2:**
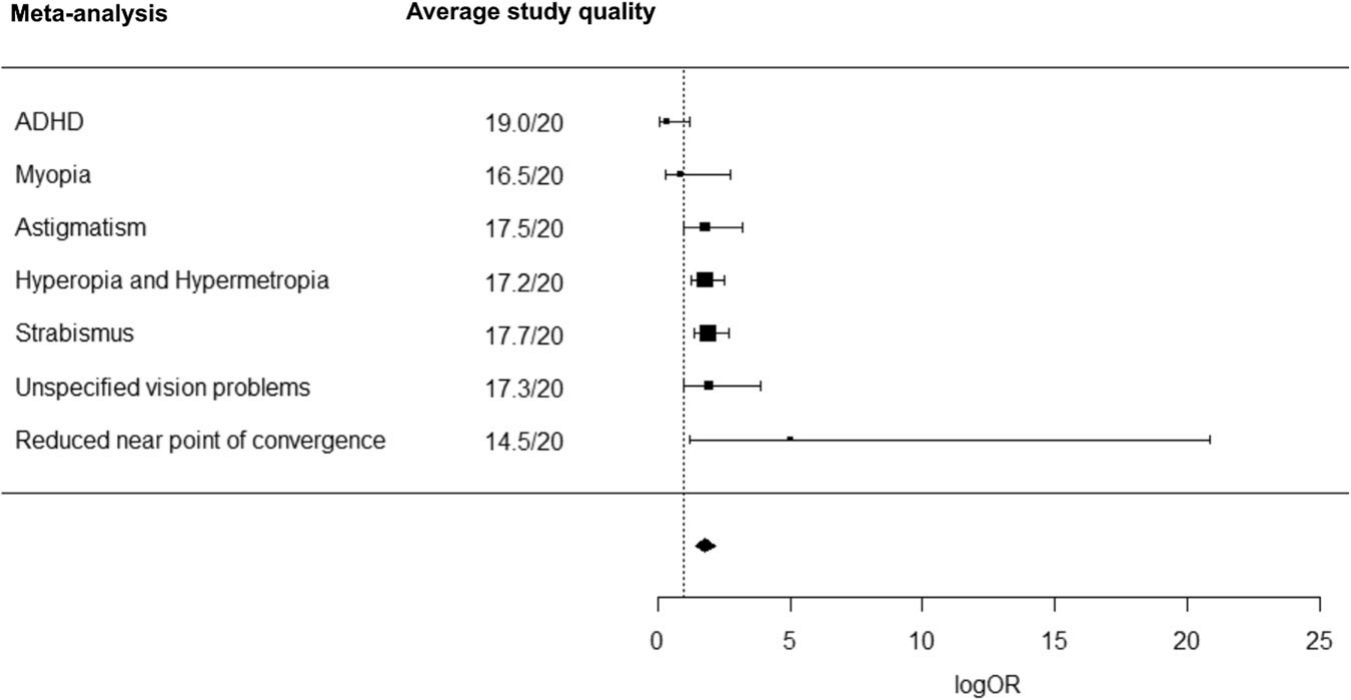
Forest plot of meta-analytic results using logOR as effect estimate. Visual representation of pooled odds ratios (logOR) for each meta-analysis on studies that reported number of people with and without a certain vision disorder/problem in people with ADHD and without ADHD, or the number of patients with ADHD in people with and without vision problems/disorders (average study quality for each meta-analysis is reported in the central panel).

**Fig. 3:**
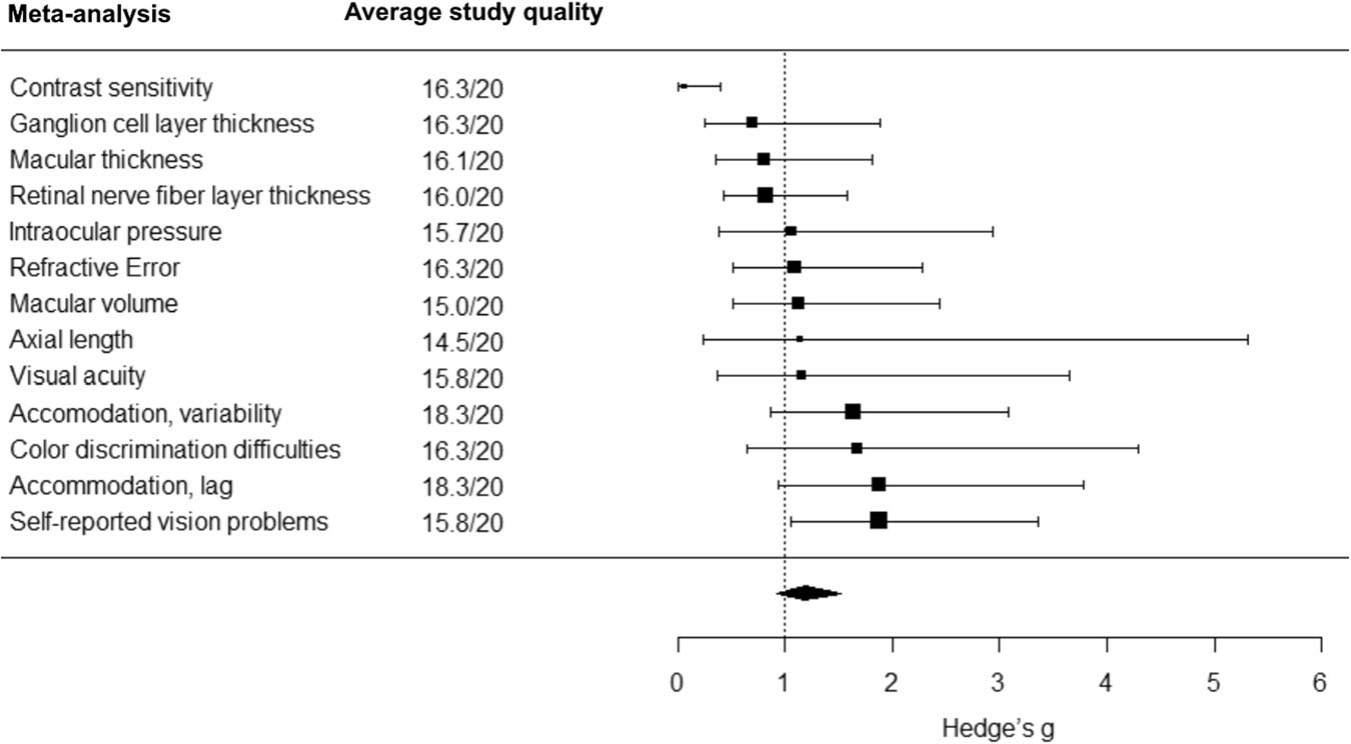
Forest plot of meta-analytic results using Hedge’s g as effect estimate. Visual representation of pooled standardized mean differences (Hedge’s g) for each meta-analysis on studies that compared outcome measures between people with and without ADHD (average study quality for each meta-analysis is reported in the central panel).

Out of 15,456 de-duplicated references initially retrieved, we screened 65 potentially eligible full texts, of which 28 were excluded (reasons for exclusion are in Supplementary 4). Thirty-seven studies met inclusion criteria and were included in the systematic review, together with six additional studies identified from references of retrieved articles (4,009,538 participants in total) (Fig. 1, Table 1). All these 43 studies were included in the narrative synthesis and 35 studies (392,423 participants with ADHD; 2,858,482 without ADHD) were included in the meta-analyses (Figs. 1, 2, 3). The following sections report the results of the narrative synthesis and of the meta-analyses (summarized in Table 2), grouped by type of study/outcome.

**Table 2:** Summary of meta-analytic results for each outcome.

Outcome	*N* of studies	Effect			Heterogeneity		Average quality of studies included^b^
		**OR**	**95% CI**	***p***	***Q***	***p***	
ADHD^a^	4	2.91	0.90; 9.45	0.0651	109.7743	<0.0001*	19.0/20
Astigmatism	4	1.79	1.50; 2.14	<0.001*	2.3058	0.5114	17.5/20
Hyperopia and Hypermetropia	5	1.79	1.66; 1.94	<0.001*	9.3200	0.0970	17.2/20
Myopia	4	0.88	0.35; 2.25	0.7272	7.9475	0.0935	16.5/20
Reduced near point of convergence	2	5.02	1.78; 14.11	0.0022*	0.0029	0.9571	14.5/20
Strabismus	3	1.93	1.75; 2.12	0.0002*	2.3025	0.5120	17.7/20
Unspecified vision problems	3	1.94	1.38; 2.73	0.0056	6.3402	0.1751	17.3/20
		***g***	**95% CI**	***p***	***Q***	***p***	
Axial length	2	0.1305	−1.0807; 1.3417	0.8328	13.1137	0.0003*	14.5/20
Ganglion cell layer thickness	4	−0.3604	−1.0720; 0.3511	0.2323	14.7801	0.0052*	16.3/20
Intraocular pressure	3	0.0579	−0.6913; 0.8071	0.8405	13.2278	0.0102*	15.7/20
Macular thickness	8	−0.2219	−0.5910; 0.1472	0.2181	49.3732	<0.0001*	16.1/20
Macular volume	2	0.1164	−0.1909; 0.4236	0.4579	0.6813	0.4091	15.0/20
Retinal nerve fiber layer thickness	8	−0.1917	−0.4079; 0.0244	0.0821	11.8039	0.1072	16.0/20
Color discrimination difficulties	6	0.5136	0.0351; 0.9921	0.0366*	70.6120	<0.0001*	16.3/20
Contrast sensitivity	4	−2.8191	−4.8895; −0.7486	0.0118*	359.8974	<0.0001*	16.3/20
Accommodation, lag	3	0.6291	0.3003; 0.9579	0.0044*	0.6230	0.9869	18.3/20
Accommodation, variability	3	0.4922	0.2319; 0.7524	0.0029*	7.0976	0.4188	18.3/20
Refractive error	6	0.0815	−0.2555; 0.4185	0.5853	9.4744	0.2204	16.3/20
Visual acuity	4	0.1485	−0.7976; 1.0947	0.6855	20.7980	0.0003*	15.8/20
Self-reported vision problems	4	0.6326	0.4420; 0.8232	<0.0001*	16.3265	0.1767	15.8/20

*LogOR* natural logarithm of odds ratio, *CI* confidence interval, *g* Hedge’s g, *Q* Cochran’s Q.

^*^Statistically significant result.

^a^In people with and without vision problems.

^b^Based on AXIS scale scores.

### ADHD in people with and without vision problems

Three studies investigated ADHD in patients with strabismus [23, 48, 49], two in patients with unspecified vision problems [22, 50] and one in patients with Color Vision Deficiency [21]. Four studies [21–23, 49] were included in the meta-analysis (Supplementary 6). Because the pooled odds ratio was not significant, these data do not support the idea that ADHD is more prevalent in people with vision problems compared to those without vision problems (logOR = 1.0692, SE = 0.4237, 95% CI = [−0.1071; 2.2456], *t* = 2.5236, *p* = 0.0651) (Table S2, Fig. S1); similar results were found when conducting the meta-analysis only on studies on children (Supplementary 6). Cross-study heterogeneity was significant (*Q* = 109.7743; *p* < 0.0001), no publication bias was detected (Kendall’s tau = 0.2000, *p* = 0.8167) (Fig. S2a) and trim and fill analyses estimated that no study was missing due to publication bias (Fig. S2b, c). The two studies included in the narrative synthesis only [48, 50] (for which we did not have available data to compute effect sizes) reported a significantly increased prevalence of ADHD in people with vision problems [50] and in people with corrected strabismus [48] compared with those without these vision disorders.

### Vision disorders in people with and without ADHD

Seven studies analyzing the prevalence of vision disorders in people with and without ADHD were included in the meta-analyses (see Supplementary 7). Among these, four investigated astigmatism [24–26, 28], five hyperopia and hypermetropia [24–28], four myopia [24, 26–28], two reduced near point of convergence [24, 27], three strabismus [24–26] and three general vision problems [24, 51, 52].

The meta-analyses showed a significantly increased risk of astigmatism (logOR = 0.5838, SE = 0.0901, 95% CI = [0.4073; 0.7603], *z* = 6.4819, *p* < 0.001; Table S3, Fig. S3), hyperopia and hypermetropia (logOR = 0.5843, SE = 0.0308, 95% CI = [0.5050; 0.6636], *t* = 18.9427, *p* < 0.001; Table S4, Fig. S5), reduced near point of convergence (logOR = 1.6129, SE = 0.5276, 95% CI = [0.5788; 2.6469], *z* = 3.0571, *p* = 0.0022; Table S6, Fig. S9), strabismus (logOR = 0.6557, SE = 0.0299, 95% CI = [0.5604; 0.7510], *t* = 21.8953, *p* = 0.0002; Table S7, Fig. S11) and unspecified vision problems (logOR = 0.6644, SE = 0.1227, 95% CI = [0.3237; 1.0050], *t* = 5.4148, *p* = 0.0056; Table S8, Fig. S13) in people with ADHD compared to without ADHD. No increased risk of myopia (logOR = −0.1261, SE = 0.3370, 95% CI = [−1.0619; 0.8096], *t* = 0.3743, *p* = 0.7272; Table S5, Fig. S7) was found in people with ADHD compared to those without. Importantly, cross-study heterogeneity was non-significant for each of these meta-analyses and publication bias was not detected (tests for heterogeneity and funnel plots are reported in Supplementary 7a–f). When investigating unspecified vision problems in children and adolescents only (i.e., excluding [52], which was on adults), the meta-analytic model was non-significant (logOR = 0.8288, SE = 0.2440, 95% CI = [−0.2212; 1.8788], *t* = 3.3964, *p* = 0.0768), with non-significant heterogeneity (Q = 3.3973; *p* = 0.1829) and no publication bias detected (Kendall’s tau = 1.000, *p* = 0.3333).

Estimation from trim and fill analyses showed that no study was missing due to publication bias in the meta-analysis conducted on astigmatism (Fig. S4b). However, one study was probably missing due to publication bias for the meta-analysis on hyperopia and hypermetropia (Fig. S6b, c). Specifically, since trim and fill analyses cannot be performed on multilevel models, we performed two trim and fill sensitivity analyses for studies reporting on hyperopia and hypermetropia (each with a single effect size from [27]): in one case the uni-level meta-analytic model was not significant (*p* changed from <0.0001 to 0.0763), while in the other case the uni-level meta-analytic model remained significant (*p* did not change from <0.0001). We performed two trim and fill sensitivity analyses for studies reporting data on myopia (each with a single effect size from [27]; Fig. S8b, c), for which one study was estimated as missing due to publication bias: in both cases the uni-level meta-analytic model remained not significant (*p* changed from 0.9978 to 0.5704, and from 0.9088 to 0.7989, respectively). Although for the meta-analysis on strabismus two studies were estimated as missing due to publication bias, the two trim and fill sensitivity analyses we conducted (each with a single effect size from [25]; Fig. S8b, c), in both cases the uni-level meta-analytic models remained significant (*p* did not change from <0.0001). For unspecified vision problems (Fig. S14b–e), one study was estimated as missing due to publication bias in two out of four trim and fill sensitivity analyses conducted (excluding one effect size each time from refs. [24, 52]); however, the meta-analysis remained significant (all *p* < 0.0023). Trim and fill analyses could not be performed for the meta-analysis on NPC, because only two studies were included in such meta-analyses.

Two studies (for which we did not have available data to compute effect sizes) were included in the narrative synthesis. Gronlund et al. [24] found a similar prevalence of anisometropia in people with and without ADHD, but a significantly increased prevalence of heterophoria in ADHD (in line with findings from the meta-analysis on strabismus). Fabian et al. [53] did not find any significant difference in heterophoria at distance between children with and without ADHD, and no difference in the prevalence of convergence insufficiency in children with and without ADHD.

### Anatomic ocular measures in people with and without ADHD

Eleven studies analyzing anatomic ocular measures in people with and without ADHD were included in the meta-analyses. Two for axial length [24, 54], four for ganglion cell layer thickness [55–58], three for intraocular pressure [56, 59, 60], eight for macular thickness [30, 54, 56–59, 61, 62], two for macular volume [61, 62] and eight for RNFL thickness [30, 54–58, 61, 62].

The meta-analyses showed no significant differences between people with and without ADHD on axial length (Hedge’s *g* = 0.1305, SE = 0.6180, 95% CI = [−1.0807; 1.3417], *z* = 0.2112, *p* = 0.8328; Table S9, Fig. S15), ganglion cell layer thickness (Hedge’s *g* = −0.3604, SE = 0.2563, 95% CI = [−1.0720; 0.3511], *t* = −1.4065, *p* = 0.2323; Table S10, Fig. S17), intraocular pressure (Hedge’s *g* = 0.0579, SE = 0.2698, 95% CI = [−0.6913; 0.8071], *t* = 0.2147, *p* = 0.8405; Table S11, Fig. S19), macular thickness (Hedge’s *g* = −0.2219, SE = 0.1721, 95% CI = [−0.5910; 0.1472], *t* = −1.2895, *p* = 0.2181; Table S12, Fig. S21), macular volume (Hedge’s *g* = 0.1164, SE = 0.1568, 95% CI = [−0.1909; 0.4236], *z* = 0.7423, *p* = 0.4579; Table S13, Fig. S23) or RNFL thickness (Hedge’s *g* = −0.1917, SE = 0.1103, 95% CI = [−0.4079; 0.0244], *z* = −1.7386, *p* = 0.0821; Table S14, Fig. S25). Of note, cross-study heterogeneity was significant for all meta-analyses (except for those on macular volume and RNFL thickness), but publication bias was not detected (tests for heterogeneity and funnel plots are reported in Supplementary 8a–f). The meta-analytic results for ganglion cell layer thickness, macular thickness and RNFL thickness did not change when we only included studies on children and adolescents (i.e., excluding studies on adults) (see Supplementary 8b, e, f).

Estimation from trim and fill analyses showed that no study was missing due to publication bias in the meta-analyses conducted on GCLT (Fig. S18b, c), intraocular pressure (Fig. S20b–e), macular thickness (Fig. S22b). Although for the meta-analysis on RNFL thickness two studies were estimated as missing due to publication bias (Fig. S26b), the trim and fill sensitivity analysis showed no change in the non-significance of the pooled effect size (*p* changed from 0.0821 to 0.5660). Trim and fill analyses could not be performed for the meta-analyses on axial length and macular volume, because only two studies were included in such meta-analyses.

Among the 10 studies that could not be included in the meta-analysis (for which we did not have available data to compute effect sizes), Ababneh et al. [27] found a similar prevalence of abnormal central foveal thickness (and, therefore, no significant differences in macular thickness) in people with and without ADHD. Moreover, while they [27] reported reduced near point of convergence in people with ADHD compared to without, Fabian et al. [53] reported *increased* near point of convergence in ADHD. Karaca et al. [28] found no significant differences between children with ADHD and without ADHD on convergence and divergence amplitudes for either distance or at near vision. Ulucan Atas et al. [30] did not find significant differences between children with and without ADHD on macular ganglion cell complex thickness.

Gronlund et al. [24] analyzed ocular fundus photographs in children with and without ADHD and found smaller optic disc area, smaller neuroretinal rim area, smaller optic cup area, lower index of tortuosity for arteries and lower index of tortuosity for veins associated with ADHD. They also reported significantly increased inner canthial distance in children with ADHD [24]. Similarly. Bodur et al. [55] reported significantly reduced optical nerve thickness in children with ADHD compared to those without. Ayyildiz et al. [54] found increased corneal thickness and reduced corneal curvature radius in children with ADHD, but no significant differences in corneal diameter. Conversely, Ababneh et al. [27] found no significant differences in corneal curvature power or maximum curvature power between children with and without ADHD. Werner et al. [63] and Bubl et al. [64] found significantly elevated retinal background noise in adults with ADHD. Conversely, Bubl et al. [65] analyzed the pattern electroretinogram in relation to different type of contrasts and found no significant difference between adults with and without ADHD.

### Differences on functional measures of vision in people with and without ADHD

Ten studies analyzing functional measures of vision in people with and without ADHD were included in the meta-analyses (Supplementary 9). Among these, six investigated color vision [31, 32, 34–36, 66], and four contrast sensitivity [29, 30, 32, 34].

The meta-analysis on color vision showed significantly increased difficulties and errors in color discrimination in people with ADHD compared to those without (Hedge’s *g* = 0.5136, SE = 0.2307, 95% CI = [0.0351; 0.9921], *t* = 2.2259, *p* = 0.0366; Table S15, Fig. S27). Another meta-analysis showed reduced contrast sensitivity in people with ADHD, compared to those without (Hedge’s *g* = −2.8191, SE = 0.9503, 95% CI = [−4.8895; −0.7486], *t* = −2.9666, *p* = 0.0118; Table S16, Fig. S29). However, cross-study heterogeneity was significant for both meta-analyses, and publication bias was detected (tests for heterogeneity and funnel plots are reported in Supplementary 9a, b). Both meta-analytic models remained significant when only including studies on children and adolescents (see Supplementary 9a, b).

We performed two trim and fill sensitivity analyses for studies reporting on color discrimination (one excluding largest effect sizes for each study reporting more than one effect size, and the other excluding smallest effect sizes; Fig. S28b, c): in the former case, two studies were estimated as missing, and the uni-level meta-analytic model was not significant anymore (*p* changed from 0.0056 to 0.1104), while in the latter no studies were estimated as missing but the uni-level meta-analytic model became non-significant (*p* = 0.1903). Trim and fill sensitivity analyses were also performed for studies reporting on contrast sensitivity (one excluding largest effect sizes for each study reporting more than one effect size, and the other excluding smallest effect sizes; Fig. S30b, c): in both cases, no studies were estimated as missing, but the uni-level meta-analytic models became non-significant (both *p* > 0.05).

Findings from Bartgis et al. [67] and Kim et al. [33] for which we could not compute effect sizes and were therefore included in narrative synthesis only, were in line with these meta-analyses, showing significantly reduced contrast sensitivity in ADHD compared to those without. Brown et al. [68] analyzed flicker fusion thresholds, i.e., the frequency at which two sources of light with different contrast were perceived differently but did not find any differences between children with and without ADHD.

### Differences on measures of visual acuity in people with and without ADHD

Eleven studies analyzing measures of visual acuity in people with and without ADHD were included in the meta-analyses (Supplementary 10). Among these, three investigated lag and variability of the accommodative response [69–71], six refractive error [24, 28, 57, 59, 69, 70] and four visual acuity [34, 53, 69, 72].

The meta-analyses for accommodation reported significantly increased lag (Hedge’s *g* = 0.6291, SE = 0.1279, 95% CI = [0.3003; 0.9579], *t* = 4.9179, *p* = 0.0044; Table S17, Fig. S31) and variability (Hedge’s *g* = 0.4039, SE = 0.1041, 95% CI = [0.1685; 0.6393], *t* = 3.8807, *p* = 0.0037; Table S18, Fig. S33) in people with ADHD compared to those without. For both meta-analyses, cross-study heterogeneity was non-significant, and publication bias was not detected (Supplementary 10a, b). Estimation from trim and fill analyses showed that no study was missing due to publication bias in the meta-analyses conducted on lag (Fig. S32b, c) or variability (Fig. S34b, c).

Refractive Error (measured through Spherical Equivalents) did not significantly differ between people with and without ADHD (Hedge’s *g* = 0.0815, SE = 0.1425, 95% CI = [−0.2555; 0.4185], *t* = 0.5718, *p* = 0.5853; Table S19, Fig. S35). Cross-study heterogeneity was non-significant (*Q* = 9.4744, *p* = 0.2204) and publication bias was not detected (Kendall’s tau = −0.3571, *p* = 0.2751) (Fig. S36). Trim and fill sensitivity analyses suggested that two studies were estimated as missing, with the uni-level meta-analytic model remaining not significant (all *p* > 0.0793; Fig. 36b–e).

Visual acuity did not differ between people with and without ADHD (Hedge’s *g* = 0.1485, SE = 0.3408, 95% CI = [−0.7976; 1.0947], *t* = 0.4358, *p* = 0.6855; Table S20, Fig. S37). Cross-study heterogeneity was significant (*Q* = 20.7980, *p* = 0.0003) and publication bias was not detected (Kendall’s tau = 0.6000, *p* = 0.2333) (Fig. S38). Estimation from trim and fill analyses showed that no study was missing due to publication bias in the meta-analyses conducted on visual acuity (Fig. S38b, c).

Among the studies that could not be included in the meta-analysis (for which we did not have available data to compute effect sizes), Fabian et al. [53] found no differences between people with and without ADHD on amplitude of the accommodative response. Lower stereoacuity in ADHD was reported by Gronlund et al. [24] and Karaca et al. [28] but not by Fabian et al. [53], Ababneh et al. [27], Fabian et al. [53], Kim et al. [34], and Martin et al. [73] found no differences in refraction between children with and without ADHD, in line with the meta-analysis. Reduced visual acuity in ADHD was found by Gronlund et al. [24] and Martin et al. [73] while Ababneh et al. [27] and Sánchez-Guillén et al. [57] found no significant differences between children with and without ADHD on visual acuity, in line with the findings from the meta-analysis.

### Differences on self-reported vision problems in people with and without ADHD

Four studies analyzing self-reported vision problems in people with and without ADHD were included in the meta-analyses [32, 35, 69, 74] (Supplementary 11). Increased self-reported vision problems were found in ADHD compared to those without (Hedge’s *g* = 0.6326, SE = 0.0875, 95% CI = [0.4420; 0.8232], *t* = 7.2322, *p* < 0.0001; Table S21, Fig. S39). Cross-study heterogeneity was not significant (*Q* = 16.3265, *p* = 0.1767) but publication bias was detected (Kendall’s tau = 0.9487, p < .0001) (Fig. S40). Trim and fill analyses were conducted; one study was estimated as missing and the meta-analysis remained significant (all *p* < 0.0479) both when conducting the trim and fill analysis on all effect sizes except the largest effect sizes for [35], and when conducting the same analysis excluding the smallest effect sizes for [35]. When investigating self-reported vision problems in children and adolescents (i.e., excluding [32, 35] which however reduced the sample size), the meta-analytic model was non-significant (Hedge’s *g* = 0.5827, SE = 0.3723, 95% CI = −0.1469; 1.3124], *t* = 1.5653, *p* = 0.1175) with no significant heterogeneity (Q = 3.2268; *p* = 0.0724). No additional studies were included for narrative synthesis only.

## Discussion

We conducted a systematic review and meta-analysis to investigate the association between ADHD and disorders or problems of vision. We found evidence of an association between ADHD and reduced color discrimination and contrast sensitivity, atypical accommodative response and convergence. No association between ADHD and visual acuity or refractive error was detected, and we did not find evidence of an association between ADHD and anatomic ocular measures (axial length, ganglion cell layer thickness, intraocular pressure, macular thickness, macular volume, RNFL thickness). However, we found an association between ADHD and astigmatism, hyperopia and hypermetropia, and strabismus (but not myopia), but we did not detect a higher prevalence of ADHD in patients diagnosed with problems of vision.

Our findings reaffirm the importance of physical and visual examinations in evaluating patients with ADHD, as suggested by multiple practice guidelines [75–77]. Various disorders of vision, including strabismus [78] and refractive errors [79–82], can present with features that mimic neurocognitive features of ADHD [83–85]. Impaired perception may influence not only cognitive function but also long-term psychosocial development by diminishing engagement in activities [86]. Therefore, we speculate that the presence of vision problems from an early age—especially if not appropriately and promptly treated—may be partly associated with increased risk for ADHD and that untreated visual impairment could exacerbate neurocognitive symptoms in children with ADHD. Although our findings demonstrate multiple relationships between ADHD, disorders of vision and impaired measures of vision, it does not suggest how this complex relationship should be interpreted.

This is further confounded by the close and intertwined relationship between perception and higher-level cognitive functions, and an incomplete understanding of the pathophysiology of ADHD. So far, the neurocognitive symptoms that define ADHD are largely conceived of as arising from structural and molecular abnormalities in the brain [9]. The brain and retina share embryological origins [7, 8, 10] and structural abnormalities of the eye have been detected in neuropsychiatric disorders with a genetic overlap with ADHD [87], including schizophrenia [88–90], bipolar disorder [91] and autism [92]. Thus, we expected to find an association between ADHD and anatomic structural abnormalities of the eye and the retina, but we did not. Contrary to Li et al. [19] we found no differences between ADHD and controls on retinal fiber layer thickness; our finding, however, emerged from a larger pool of studies.

While we did not detect a relationship between ADHD and anatomic measures, ADHD was associated with increased risk of disorders caused by atypical corneal curvature and eye shape, and problems in controlling eye muscles (e.g., astigmatism, hyperopia and hypermetropia, reduced near point of convergence, and strabismus). Although the development of specific components of the retina may not be affected in ADHD, other major structures of the eye may develop atypically in ADHD, increasing the risk of problems of vision.

In contrast to the anatomic measures, we detected an association between ADHD and diminished contrast sensitivity and impaired color discrimination, in which the retina is potentially implicated. Impairments in these functions have also been reported in neuropsychiatric disorders in which catecholaminergic transmission is affected (e.g., schizophrenia [88, 93], and Parkinson’s [94, 95]). The association between ADHD and diminished contrast sensitivity and color discrimination may therefore derive from altered functioning of retinal dopaminergic neurons in ADHD [96, 97]. Elevated retinal background noise, another possible sign of dopaminergic dysfunction, is associated with increased severity of ADHD symptoms [63, 64] and it is likely to be associated with inattention and distractibility due to involuntary orienting of attention towards irrelevant information [98, 99]. Dopamine, on the other hand, has been shown to reduce neuronal noise [100–103], as does stimulant medication [63, 97, 99, 104].

Given that the retina receives little if any top-down connections from higher cortical regions [105], deficits in contrast sensitivity and color discrimination in ADHD may arise at the level of the retina itself. Moreover, considering that normalization of neuronal background noise elsewhere in the brain is observed following administration of stimulants in conjunction with reduced symptoms of ADHD [97, 99, 104], one might hypothesize that stimulant-induced normalization of retinal background noise could be associated with improved measures of visual function such as contrast sensitivity or color vision sensitivity. However, research in this area is limited, and only a few studies included in our review (e.g., [29],) found medication-related improvement in contrast sensitivity.

Additionally, we found a significant relationship between ADHD and accommodation lag and variability. This result is consistent with our finding of an association between ADHD and convergence insufficiency, and with other studies that found altered accommodative functions in other neurodevelopmental disorders [106]. Accommodation is the process by which the ciliary muscles of the eye adjust optical power to maintain focus of an object at various distances [107]. While the presence of oculomotor abnormalities in ADHD is well established [20], it is difficult to interpret this finding given the overlap between the neural systems that regulate attention and ocular dynamics [108, 109]. The relationship between attention and accommodative function has been proposed to be bidirectional [110, 111], so that accommodative dysfunction reduces attentional resources, induces asthenopia, visual discomfort and decreases task performance efficiency [111, 112], contributing to inattentive symptoms. However, it is also possible that the differences in accommodative performance could arise from the neurocognitive deficits of ADHD [113–115]. Because the accommodative response is influenced by the autonomic nervous system [116], accommodative dysfunction can also be understood in the context of autonomic hypo-arousal seen during cognitive, reward and socio-emotional tasks in ADHD [117].

Despite finding an association between ADHD, disorders of vision and multiple measures of vision, we did not detect a relationship for either refractive error or visual acuity, which is inconsistent with our other findings. If ADHD is indeed a risk factor for astigmatism, hyperopia, strabismus, and accommodative dysfunction, we would expect these measures to be similarly implicated. For example, both astigmatism and hyperopia require refractive errors beyond certain clinical thresholds in order to be diagnosed. One explanation for this discrepancy is that, in many of the included studies, visual acuity was often reported as *best-corrected* visual acuity.

If unaddressed, sensory deficits may contribute to functional impairment in ADHD [118–121], including impairments in driving performance [122, 123], emotional recognition and social functioning [124]. The close relationship between perceptual deficits and neurocognitive deficits [125, 126] raises the important question of to what extent, if any, visual problems are associated with ADHD and the neurocognitive symptoms that define the disorder. Given reports of children with disorders of vision being misdiagnosed with ADHD [84, 85, 127, 128] and the effect of these disorders on attention [83], it is possible that the presence of problems of vision confounds the diagnosis of ADHD, and vice versa. Furthermore, another question is if visual deficits could be an underrecognized treatment target in some patients with ADHD. For pediatric patients having both ADHD and vision problems, future work should address their independent contributions to cognitive and global functioning. Identifying and promptly implementing specific treatments for both vision problems and ADHD symptoms may lead to more positive interventional outcomes. For example, inattentive symptoms secondary to disorders of vision could be reduced by addressing the underlying visual dysfunction, at least in some patients [78, 79].

Clinicians should be aware that using neuropsychological instruments that do not account for potential visual deficits may overstate the presence and magnitude of neuropsychological deficits. Similarly, assessing visual deficits without accounting for neuropsychological deficits may overstate the presence of vision problems. Additionally, given that normal perception function changes throughout life, longitudinal studies are needed to clarify the causal links between ADHD and visual deficits. Generalization of our results is limited by the underrepresentation of females given that there are gender and sex differences with ADHD [129], disorders of vision [130] and normal visual function [131]. Future research should also consider additional risk factors that are associated with ADHD and multiple disorders of vision, including low socioeconomic status, young maternal age, low birth weight, congenital infections [132, 133] and prematurity [81, 82, 134–136].

Considering the scarce and mixed evidence investigating the influence of medication for ADHD on retinal background noise and measures of visual function in which retinal dopaminergic neurons are implicated, this is another important area that should be explored by future research. For example, Martin et al. [73] found significant improvements in visual acuity and visual fields in children with ADHD after treatment with stimulants, while Gronlund, Mezer and Wygnanski-Jaffe [24, 137] did not report this effect. Similarly, Redondo et al. [71] found that while there were significant differences in accommodation between ADHD children and controls, stimulants did not significantly improve accommodation in the ADHD group. Various case reports document potential ocular side effects of both stimulants and non-stimulants in patients with ADHD such as accommodation dysfunction, cataracts, mydriasis, cataracts and increased intraocular pressure [138–142]. Conversely, other authors [60, 143] found no relationship between intraocular pressure and treatment with these medications. Stimulants and various non-stimulant medications used to treat ADHD are adrenergic agonizts, and therefore may affect autonomic regulation of various ocular structures [144]. Given the sparse literature on this topic, the effects of stimulants may confound the relationship between ADHD and measures of vision.

The present study has several limitations. Some meta-analyses only included a few studies. Moreover, cross-study heterogeneity was significant for the meta-analyses on the prevalence of ADHD in people with and without vision problems, all meta-analyses on anatomic measures (except for macular volume and RNFL thickness), color vision and contrast sensitivity, and visual acuity. These results, although statistically significant, need to be considered cautiously. Heterogeneity is probably due to differences in the methodology used for obtaining anatomic measures, different paradigms used to investigate color vision and contrast sensitivity, and heterogeneity in the ascertainment and diagnosis of study participants. Lastly, publication bias was detected for the meta-analyses of studies on color vision and contrast sensitivity, and for differences in self-reported vision problems. The trim and fill analyses for color vision and contrast discrimination found that, after correcting for publication biases, their pooled effect sizes were not statistically significant. Considering that measures of vision are less variable than measures of ADHD symptoms, they may not be as sensitive in capturing the heterogeneity of ADHD compared with diagnostic measures. Moreover, future research should investigate if specific disorders of vision are specifically associated with ADHD or also with other neurodevelopmental conditions, e.g., autism. Lastly, because most of the studies in the meta-analysis were sampled from clinically referred populations, the generalizability of our results is limited to such samples due to Berkson’s bias (i.e., a selection bias that can arise when the sample is taken not from the general population, but from a subpopulation) and other methodological issues, as previously discussed.

In conclusion, we found meta-analytic evidence of a significant association of ADHD with self-reported and objectively ascertained functional vision problems, but not with structural or anatomic alterations. Further studies are needed to investigate what type of causal relationships exist between ADHD and specific problems of vision, and how much one can explain the others.

## Supplementary information

Supplemental Materials
