# Supplementary material for: Association between ADHD and vision problems. A systematic review and meta-analysis
Source: Mol Psychiatry. 2022 Aug 5;28(1):410–22. doi: 10.1038/s41380-022-01699-0 (PMC9812778; doi:10.1038/s41380-022-01699-0)
Supplement: Supplementary file 1 — Supplemental Materials [file 41380_2022_1699_MOESM1_ESM.pdf]

**Supplementary Materials**

Supplement 1: PRISMA Checklist ........................................................................................ 5
Supplement 2: Search strategy (last search: 16th November 2021) ........................................ 11
Supplement 3. Changes/additions to the original protocol, with reasons for the changes....... 12
Supplement 4. List of excluded papers after full-text screening ........................................... 13
Supplement 5. Quality appraisal of studies included in the review ........................................ 17
Table S1. Appraisal tool for Cross-Sectional Studies (AXIS)............................................ 17
Supplement 6: Meta-analysis of studies investigating the prevalence of ADHD in people with and without vision problems ........................................................................................ 21
Table S2. Description of studies ........................................................................................ 21
Figure S1. Forest plot.......................................................................................................... 21
Figure S2. Funnel plots (a. no trim and fill analysis; b. trim and fill analysis excluding Mohney, 2008.2; c. trim and fill analysis excluding Mohney, 2008.1).............................. 22
Supplement 7: Meta-analyses of studies investigating the prevalence of vision problems/conditions in people with and without ADHD ...................................................... 24
a. Astigmatism ...................................................................................................................... 24
Table S3. Description of studies ..................................................................................... 24
Figure S3. Forest plot....................................................................................................... 24
Figure S4. Funnel plots (a. no trim and fill analysis; b. trim and fill analysis) ............... 25
b. Hyperopia and Hypermetropia.......................................................................................... 26
Table S4. Description of studies ..................................................................................... 26
Figure S5. Forest plot....................................................................................................... 26
Figure S6. Funnel plots (a. no trim and fill analysis; b. trim and fill analysis excluding Ababneh, 2020.2; c. trim and fill analysis excluding Ababneh, 2020.1)........................ 27
c. Myopia .............................................................................................................................. 28
Table S5. Description of studies ..................................................................................... 28
Figure S7. Forest plot....................................................................................................... 28
Figure S8. Funnel plots (a. no trim and fill analysis; b. trim and fill analysis excluding Ababneh, 2020.2; c. trim and fill analysis excluding Ababneh, 2020.1)........................ 29
d. Reduced near point of convergence ................................................................................. 30
Table S6. Description of studies ..................................................................................... 30
Figure S9. Forest plot....................................................................................................... 30
Figure S10. Funnel plot.................................................................................................... 31
e. Strabismus ......................................................................................................................... 32
Table S7. Description of studies ..................................................................................... 32
Figure S11. Forest plot..................................................................................................... 32

Figure S12. Funnel plots (a. no trim and fill analysis; b. trim and fill analysis excluding Ho, 2020.2; c. trim and fill analysis excluding Ho, 2020.1) 33

f. Unspecified Vision problems 34

Table S8. Description of studies 34

Figure S13. Forest plot 34

Figure S14. Funnel plots (a. no trim and fill analysis; b. trim and fill analysis excluding Grönlund, 2007.2 and Kooij, 2014.2; c. trim and fill analysis excluding Grönlund, 2007.1 and Kooij, 2014.1; d. trim and fill analysis excluding Grönlund, 2007.2 and Kooij, 2014.1; e. trim and fill analysis excluding Grönlund, 2007.1 and Kooij, 2014.2) 35

Supplement 8: Results, forest and funnel plots for studies investigating the differences on anatomic (measurable) measures 37

a. Axial Length (of the eye) 37

Table S9. Description of studies 37

Figure S15. Forest plot 37

Figure S16. Funnel plot 38

b. Ganglion Cell Layer Thickness 39

Table S10. Description of studies 39

Figure S17. Forest plot 39

Figure S18. Funnel plots (a. no trim and fill analysis; b. trim and fill analysis excluding Isik, 2020.2; c. trim and fill analysis excluding Isik, 2020.1) 40

c. Intraocular pressure 42

Table S11. Description of studies 42

Figure S19. Forest plot 42

Figure S20. Funnel plots (a. no trim and fill analysis; b. trim and fill analysis excluding Guvenmez, 2020.2 and Isik, 2014.2; c. trim and fill analysis excluding Guvenmez, 2007.1 and Isik, 2014.1; d. trim and fill analysis excluding Guvenmez, 2007.2 and Isik, 2014.1; e. trim and fill analysis excluding Guvenmez, 2007.1 and Isik, 2014.2) 43

d. Macular Thickness 45

Table S12. Description of studies 45

Figure S21. Forest plot 46

Figure S22. Funnel plots (a. no trim and fill analysis; b. trim and fill analysis excluding largest effect sizes for each study reporting more than one effect size) 47

e. Macular Volume 48

Table S13. Description of studies 48

Figure S23. Forest plot 48

Figure S24. Funnel plot 49

f. Retinal Nerve Fiber Layer Thickness 50

Table S14. Description of studies 50

Figure S25. Forest plots 50

Figure S26. Funnel plots (a. no trim and fill analysis; b. trim and fill analysis) 51

Supplement 9: Results, forest and funnel plots for studies investigating differences on functional measures of vision ..... 52

a. Color discrimination ..... 52

Table S15. Description of studies ..... 52

Figure S27. Forest plot ..... 53

Figure S28. Funnel plots (a. no trim and fill analysis; b. trim and fill analysis excluding largest effect sizes for each study reporting more than one effect size, c. trim and fill analysis excluding smallest effect sizes for each study reporting more than one effect size) ..... 54

b. Contrast sensitivity ..... 56

Table S16. Description of studies ..... 56

Figure S29. Forest plot ..... 57

Figure S30. Funnel plots (a. no trim and fill analysis; b. trim and fill analysis excluding largest effect sizes for each study reporting more than one effect size, c. trim and fill analysis excluding smallest effect sizes for each study reporting more than one effect size) ..... 58

Supplement 10: Results, forest and funnel plots for studies investigating differences on measures of visual acuity ..... 60

a. Accommodation: lag ..... 60

Table S17. Description of studies ..... 60

Figure S31. Forest plot ..... 60

Figure S32. Funnel plots (a. no trim and fill analysis; b. trim and fill analysis excluding largest effect sizes for Redondo, 2018 and Redondo, 2020b, c. trim and fill analysis excluding smallest effect sizes for Redondo, 2018 and Redondo, 2020b) ..... 61

b. Accommodation: variability ..... 63

Table S18. Description of studies ..... 63

Figure S33. Forest plot ..... 63

Figure S34. Funnel plots (a. no trim and fill analysis; b. trim and fill analysis excluding largest effect sizes for Redondo 2018, Redondo 2020a and Redondo 2020b, c. trim and fill analysis excluding smallest effect sizes for Redondo 2018, Redondo 2020a and Redondo 2020b) ..... 64

c. Refractive Error (Spherical Equivalents) ..... 66

Table S19. Description of studies ..... 66

Figure S35. Forest plot ..... 66

Figure S36. Funnel plots (a. no trim and fill analysis; b. trim and fill analysis excluding Bae 2019.2 and Gronlund 2007.2, c. trim and fill analysis excluding Bae 2019.1 and Gronlund 2007.1, d. trim and fill analysis excluding Bae 2019.2 and Gronlund 2007.1, e. trim and fill analysis excluding Bae 2019.1 and Gronlund 2007.2) ..... 67

d. Visual Acuity ..... 69

Table S20. Description of studies ..... 69

Figure S37. Forest plot ..... 69

Figure S38. Funnel plots (a. no trim and fill analysis; b. trim and fill analysis excluding DeCarlo 2014.2, c. trim and fill analysis excluding DeCarlo 2014.1)............................70

Supplement 11: Results, forest and funnel plots for studies investigating differences on self-reported vision problems..........................................................................................71

Table S21. Description of studies ......................................................................71

Figure S39. Forest plot......................................................................................71

Figure S40. Funnel plots (a. no trim and fill analysis; b. trim and fill analysis excluding largest effect sizes for Kim, 2014c, c. trim and fill analysis excluding smallest effect sizes for Kim, 2014c).........................................................................................72

**Supplement 1: PRISMA Checklist**

| Section and Topic | Item # | Checklist item | Location where item is reported |
|---|---|---|---|
| **TITLE** | | | |
| Title | 1 | Identify the report as a systematic review. | Title Page |
| **ABSTRACT** | | | |
| Abstract | 2 | See the PRISMA 2020 for Abstracts checklist. | Abstract |
| **INTRODUCTION** | | | |
| Rationale | 3 | Describe the rationale for the review in the context of existing knowledge. | Introduction |
| Objectives | 4 | Provide an explicit statement of the objective(s) or question(s) the review addresses. | Introduction |
| **METHODS** | | | |
| Eligibility criteria | 5 | Specify the inclusion and exclusion criteria for the review and how studies were grouped for the syntheses. | Methods, paragraph "Selection criteria" |
| Information sources | 6 | Specify all databases, registers, websites, organisations, reference lists and other sources searched or consulted to identify studies. Specify the date when each source was last searched or consulted. | Methods, paragraph "Search strategy" |
| Search strategy | 7 | Present the full search strategies for all databases, registers and websites, including any filters and limits used. | Supplement 2 |
| Selection process | 8 | Specify the methods used to decide whether a study met the inclusion criteria of the review, including how many reviewers screened each record and each report retrieved, whether they worked independently, and if applicable, details of automation tools used in the process. | Methods, paragraph "Data selection, extraction and coding" |
| Data collection process | 9 | Specify the methods used to collect data from reports, including how many reviewers collected data from each report, whether they worked independently, any processes for | Methods, paragraph "Data |

| Section and Topic | Item # | Checklist item | Location where item is reported |
|---|---|---|---|
| | | obtaining or confirming data from study investigators, and if applicable, details of automation tools used in the process. | selection, extraction and coding” |
| Data items | 10a | List and define all outcomes for which data were sought. Specify whether all results that were compatible with each outcome domain in each study were sought (e.g. for all measures, time points, analyses), and if not, the methods used to decide which results to collect. | Methods, paragraphs “Data selection, extraction and coding” and “Outcomes and assessment of study quality” |
| | 10b | List and define all other variables for which data were sought (e.g. participant and intervention characteristics, funding sources). Describe any assumptions made about any missing or unclear information. | Methods, paragraph “Data selection, extraction and coding” |
| Study risk of bias assessment | 11 | Specify the methods used to assess risk of bias in the included studies, including details of the tool(s) used, how many reviewers assessed each study and whether they worked independently, and if applicable, details of automation tools used in the process. | Methods, paragraph “Outcomes and assessment of study quality” and Supplement 5 |
| Effect measures | 12 | Specify for each outcome the effect measure(s) (e.g. risk ratio, mean difference) used in the synthesis or presentation of results. | Methods, paragraph “Data synthesis and analysis” |
| Synthesis methods | 13a | Describe the processes used to decide which studies were eligible for each synthesis (e.g. | Methods, |

| Section and Topic | Item # | Checklist item | Location where item is reported |
|---|---|---|---|
| | | tabulating the study intervention characteristics and comparing against the planned groups for each synthesis (item #5)). | paragraph "Data synthesis and analysis" |
| | 13b | Describe any methods required to prepare the data for presentation or synthesis, such as handling of missing summary statistics, or data conversions. | Methods, paragraph "Data synthesis and analysis" |
| | 13c | Describe any methods used to tabulate or visually display results of individual studies and syntheses. | Methods, paragraph "Data synthesis and analysis" |
| | 13d | Describe any methods used to synthesize results and provide a rationale for the choice(s). If meta-analysis was performed, describe the model(s), method(s) to identify the presence and extent of statistical heterogeneity, and software package(s) used. | Methods, paragraph "Data synthesis and analysis" |
| | 13e | Describe any methods used to explore possible causes of heterogeneity among study results (e.g. subgroup analysis, meta-regression). | n/a. No subgroup analyses or meta-regression conducted. |
| | 13f | Describe any sensitivity analyses conducted to assess robustness of the synthesized results. | n/a. No sensitivity analyses conducted. |
| Reporting bias assessment | 14 | Describe any methods used to assess risk of bias due to missing results in a synthesis (arising from reporting biases). | Methods, paragraphs "Outcomes and assessment of study quality" |

| Section and Topic | Item # | Checklist item | Location where item is reported |
|---|---|---|---|
| | | | and "Data synthesis and analysis", and Supplement 5 |
| Certainty assessment | 15 | Describe any methods used to assess certainty (or confidence) in the body of evidence for an outcome. | Methods, paragraph "Data synthesis and analysis" |
| **RESULTS** | | | |
| Study selection | 16a | Describe the results of the search and selection process, from the number of records identified in the search to the number of studies included in the review, ideally using a flow diagram. | Results and Figure 1 |
| | 16b | Cite studies that might appear to meet the inclusion criteria, but which were excluded, and explain why they were excluded. | Supplement 4 |
| Study characteristics | 17 | Cite each included study and present its characteristics. | Table 1 |
| Risk of bias in studies | 18 | Present assessments of risk of bias for each included study. | Supplement 5 |
| Results of individual studies | 19 | For all outcomes, present, for each study: (a) summary statistics for each group (where appropriate) and (b) an effect estimate and its precision (e.g. confidence/credible interval), ideally using structured tables or plots. | Tables 1 and 2 |
| Results of syntheses | 20a | For each synthesis, briefly summarise the characteristics and risk of bias among contributing studies. | Results |
| | 20b | Present results of all statistical syntheses conducted. If meta-analysis was done, present for each the summary estimate and its precision (e.g. confidence/credible interval) and measures of statistical heterogeneity. If comparing groups, describe the direction of the | Results, Table 2 |

| Section and Topic | Item # | Checklist item | Location where item is reported |
|---|---|---|---|
| | | effect. | |
| | 20c | Present results of all investigations of possible causes of heterogeneity among study results. | n/a |
| | 20d | Present results of all sensitivity analyses conducted to assess the robustness of the synthesized results. | n/a |
| Reporting biases | 21 | Present assessments of risk of bias due to missing results (arising from reporting biases) for each synthesis assessed. | Results |
| Certainty of evidence | 22 | Present assessments of certainty (or confidence) in the body of evidence for each outcome assessed. | n/a |
| **DISCUSSION** | | | |
| Discussion | 23a | Provide a general interpretation of the results in the context of other evidence. | Discussion |
| | 23b | Discuss any limitations of the evidence included in the review. | Discussion |
| | 23c | Discuss any limitations of the review processes used. | Discussion, |
| | 23d | Discuss implications of the results for practice, policy, and future research. | Discussion |
| **OTHER INFORMATION** | | | |
| Registration and protocol | 24a | Provide registration information for the review, including register name and registration number, or state that the review was not registered. | Abstract, Methods |
| | 24b | Indicate where the review protocol can be accessed, or state that a protocol was not prepared. | Abstract, Methods |
| | 24c | Describe and explain any amendments to information provided at registration or in the protocol. | Supplement 3 |
| Support | 25 | Describe sources of financial or non-financial support for the review, and the role of the funders or sponsors in the review. | Title Page |
| Competing interests | 26 | Declare any competing interests of review authors. | Title Page |

| Section and Topic | Item # | Checklist item | Location where item is reported |
|---|---|---|---|
| Availability of data, code and other materials | 27 | Report which of the following are publicly available and where they can be found: template data collection forms; data extracted from included studies; data used for all analyses; analytic code; any other materials used in the review. | Methods, paragraph "Data synthesis and analysis" |

*From:* Page MJ, McKenzie JE, Bossuyt PM, Boutron I, Hoffmann TC, Mulrow CD, et al. The PRISMA 2020 statement: an updated guideline for reporting systematic reviews. BMJ 2021;372:n71. doi: 10.1136/bmj.n71

**Supplement 2: Search strategy (last search: 16th November 2021)**

***Pubmed***

(ADHD [tiab] OR attention-deficit [tiab] OR Attention Deficit [tiab] OR hyperkinetic syndrome [tiab] OR hyperkinetic disorder [tiab]) AND (vision [tiab] OR visual [tiab] or color* [tiab] OR colour* [tiab] OR eye* movement* [tiab] OR saccadic movement* [tiab] OR convergence insufficiency [tiab] or Ocular coherence tomography [tiab] or Optical coherence tomography [tiab] or OCT [tiab] or perimetry [tiab] or optical [tiab])

**OVID databases:** ***PsycInfo, EMBASE+EMBASE classic, OVID Medline***

(ADHD OR attention-deficit OR Attention Deficit OR hyperkinetic syndrome OR hyperkinetic disorder) AND (vision OR visual or color* OR colour* OR eye* movement* OR saccadic movement* OR convergence insufficiency or Ocular coherence tomography or Optical coherence tomography OR OCT or perimetry or optical)

***WEB OF KNOWLEDGE***

***(Web of science (science citation index expanded), Biological abstracts, Biosis, Food science and technology abstracts)***

ADHD OR attention-deficit OR Attention Deficit OR hyperkinetic syndrome OR hyperkinetic disorder

vision OR visual or color* OR colour* OR eye* movement* OR saccadic movement* OR convergence insufficiency or Ocular coherence tomography or Optical coherence tomography OR OCT or perimetry or optical

**Supplement 3. Changes/additions to the original protocol, with reasons for the changes**

The original protocol was submitted on PROSPERO on 24th May 2021.

Upon agreement of all authors, for population studies that used surveys as the main method for data collection, we allowed the fact that ICD/DSM-based diagnoses of ADHD were not confirmed by clinicians but were self-reported or parent-reported. This was only appliable to the meta-analyses on the prevalence of ADHD in people with and without vision problems/disorders, and the prevalence of unspecified vision problems/disorders in people with reported ADHD.

**Supplement 4. List of excluded papers after full-text screening**

- Bartgis, J. D. (2006). The relation of contrast sensitivity and ADHD: Discriminant validity and correlations with laboratory measures.

  Reason for exclusion: Dissertation

- Beyer (2021). Dimensions of attention-deficit/hyperactivity disorder and sluggish cognitive tempo as predictors of executive functioning, depression, anxiety, substance use, and convergence insufficiency.

  Reason for exclusion: Dissertation

- Bilbao (2021). Distribution of visual and oculomotor alterations in a clinical population of children with and without neurodevelopmental disorders.

  Reason for exclusion: Sample size < 10

- Casal, P. V., et al. (2019). Clinical Validation of Eye Vergence as an Objective Marker for Diagnosis of ADHD in Children.

  Reason for exclusion: Not relevant to the study

- Chung, S. A., et al. (2012). Parent-Reported Symptoms of Attention Deficit Hyperactivity Disorder in Children with Intermittent Exotropia before and after Strabismus Surgery.

  Reason for exclusion: Not focused on ADHD

- DeCarlo, D. K. (2018). The Impact of Vision Impairment on Children Through the Eyes of an Optometrist.

  Reason for exclusion: Not focused on ADHD

- Dorani, F., et al (2016). Looking into the eye of ADHD. First data on photophobia in adults with ADHD.

  Reason for exclusion: Conference abstract, published paper sent by the authors and added to the study

- Elsayed, D. A. and R. M. Abdou (2015). The study of convergence insufficiency in children with attention deficit hyperactivity disorder.

  Reason for exclusion: No control group
- Friedburg, D. (2002). Prismatic correction in attention deficit disorders.

  Reason for exclusion: Editorial/Commentary
- Ghanizadeh, A. (2010). Visual fields in children with attention-deficit/hyperactivity disorder before and after treatment with stimulants.

  Reason for exclusion: Letter to the editor
- Gomes, A. and A. Barbosa (2014). Convergence insufficiency in children with attention deficit hyperactivity disorder.

  Reason for exclusion: No control group
- Granet, D. B., et al. (2005). The relationship between convergence insufficiency and ADHD.

  Reason for exclusion: No control group
- Hinkley, S., et al. (2016). Association of accommodative amplitude and lag with Attention Deficit/Hyperactivity Disorder.

  Reason for exclusion: DSM/ICD criteria not used
- Jimenez, E. C., et al. (2020). Eye Vergence Responses During an Attention Task in Adults With ADHD and Clinical Controls.

  Reason for exclusion: No control group
- Kara, K., et al. (2013). Investigation of autonomic nervous system functions by pupillometry in children with Attention-Deficit/Hyperactivity Disorder. [References].

  Reason for exclusion: Not relevant to the study

- Kara, K., et al. (2012). Pupillometric assessment of autonomic nervous system functions in children with attention deficit hyperactivity disorder.

  Reason for exclusion: Conference abstract, not relevant
- Kim, S. (2016). Color vision and its mechanisms in college students with attention deficit hyperactivity disorder.

  Reason for exclusion: Dissertation
- Kooij, S. and D. Bijlenga (2015). Looking into the eye of Attention-Deficit/Hyperactivity Disorder - First data on photophobia in adults with ADHD.

  Reason for exclusion: Conference abstract, published paper sent by the authors and added to the study
- Kuga, M., et al. (2017). Visual acuity measurement for children with developmental disorder. [Japanese].

  Reason for exclusion: Review
- Loew, S. J. and K. Watson (2013). The prevalence of symptoms of scotopic sensitivity/Meares-Irlen syndrome in subjects diagnosed with ADHD: - Does misdiagnosis play a significant role? [References].

  Reason for exclusion: No rigorous assessment of ocular/vision problems
- Mezer, E. and T. Wygnanski-Jaffe (2012). Do children and adolescents with attention deficit hyperactivity disorder have ocular abnormalities?

  Reason for exclusion: DSM/ICD criteria not used
- Moran (2021). Effects of attention deficit hyperactivity disorder and learning disability on vestibular and ocular baseline concussion assessment in pediatric athletes.

  Reason for exclusion: DSM/ICD criteria not used

- Perera, S. J. (2017). Vision problems in children and adolescents with ADHD-A preliminary survey.

  Reason for exclusion: Conference abstract

- Puig, M. S., et al. (2015). Attention-related eye vergence measured in children with attention deficit hyperactivity disorder.

  Reason for exclusion: Not relevant to the study

- Rouse, M., et al (2009). Academic behaviors in children with convergence insufficiency with and without parent-reported ADHD.

  Reason for exclusion: DSM/ICD criteria not used

- Savchuk, L. V., et al. (2018). Psychophysiological correlates of color function in children with Attention Deficit Hyperactivity Disorder.

  Reason for exclusion: Duplicate stored with a different title

- Savchuk, L. V., et al. (2016). Psychophysiological correlates of Attention Deficit Hyperactivity Disorder in children.

  Reason for exclusion: Conference abstract

- Tarakcioglu, H. N., et al. (2020). Foveal avascular zone and vessel density in children with attention deficit hyperactivity disorder.

  Reason for exclusion: No control group

## Supplement 5. Quality appraisal of studies included in the review

### *Table S1. Appraisal tool for Cross-Sectional Studies (AXIS)*

| | **Clear aims/objectives** | **Appropriate study design** | **Justified sample size** | **Clear target population** | **Representative sample** | **Appropriate selection process** | **Appropriate categorisation of non-responders** | **Appropriate measurement of risk factors and outcome variables** | **Use of appropriate instruments /measurements** | **Appropriate determination of statistical significance** | **Clear description of methods** |
|---|---|---|---|---|---|---|---|---|---|---|---|
| Ababneh 2020 | Y | Y | N | Y | Y | Y | N | Y | Y | Y | Y |
| Akmatov 2021 | Y | Y | Y | Y | Y | Y | Y | Y | Y | Y | Y |
| Aslan 2020 | Y | Y | N | Y | Y | N | N | Y | Y | Y | Y |
| Ayyildiz & Ayyildiz 2019 | Y | Y | N | Y | Y | Y | Y | Y | Y | Y | Y |
| Bae 2019 | Y | Y | N | Y | N | Y | N | Y | Y | Y | Y |
| Banaschewski 2006 | Y | Y | N | Y | N | Y | N | Y | Y | Y | Y |
| Bartgis 2009 | Y | Y | N | Y | N | Y | N | Y | Y | Y | Y |
| Berger 2016 | Y | Y | Y | Y | Y | Y | Y | Y | Y | Y | Y |
| Bodur 2018 | Y | Y | N | Y | Y | Y | N | Y | Y | Y | Y |
| Brown 2020 | Y | Y | N | Y | Y | Y | Y | Y | Y | Y | Y |
| Bubl 2013 | Y | Y | N | Y | Y | Y | N | Y | Y | Y | Y |
| Bubl 2015 | Y | Y | N | Y | Y | Y | N | Y | Y | Y | Y |
| DeCarlo 2014 | Y | Y | N | Y | Y | Y | Y | Y | Y | Y | Y |
| DeCarlo 2016 | Y | Y | Y | Y | Y | Y | Y | Y | Y | Y | Y |
| Donmez 2020 | Y | Y | N | Y | Y | Y | N | Y | Y | Y | Y |
| Fabian 2013 | Y | Y | N | Y | N | Y | N | Y | Y | Y | Y |
| Farrar 2001 | Y | Y | N | Y | N | Y | N | Y | Y | Y | Y |
| Grönlund 2007 | Y | Y | N | Y | Y | Y | N | Y | Y | Y | Y |
| Guvenmez 2020 | Y | Y | N | Y | Y | Y | N | Y | Y | Y | Y |
| Hergüner 2018 | Y | Y | N | Y | N | Y | N | Y | Y | Y | Y |

| | **Clear aims/objectives** | **Appropriate study design** | **Justified sample size** | **Clear target population** | **Representative sample** | **Appropriate selection process** | **Appropriate categorisation of non-responders** | **Appropriate measurement of risk factors and outcome variables** | **Use of appropriate instruments /measurements** | **Appropriate determination of statistical significance** | **Clear description of methods** |
|---|---|---|---|---|---|---|---|---|---|---|---|
| Ho 2020 | Y | Y | Y | Y | Y | Y | Y | Y | Y | Y | Y |
| Işik & Kaygisiz 2020 | Y | Y | N | Y | Y | Y | N | Y | Y | Y | Y |
| Karaca 2020 | Y | Y | N | Y | Y | Y | N | Y | Y | Y | Y |
| Kim 2014a | Y | Y | N | Y | Y | Y | N | Y | Y | N | Y |
| Kim 2014b | Y | Y | N | Y | Y | Y | N | Y | Y | N | Y |
| Kim 2014c | Y | Y | N | Y | Y | Y | N | Y | Y | Y | Y |
| Kim 2015 | Y | Y | N | Y | Y | Y | N | Y | Y | N | Y |
| Kooij & Bijlenga 2014 | Y | Y | Y | Y | Y | Y | Y | Y | Y | Y | Y |
| Martin 2008 | Y | Y | N | Y | Y | Y | N | Y | Y | Y | Y |
| McBride & Bijan 2017* | Y | Y | Y | Y | Y | Y | N | Y | N | N | N |
| Merdler 2017 | Y | Y | Y | Y | Y | Y | N | Y | Y | Y | Y |
| Mohney 2008 | Y | Y | Y | Y | Y | Y | N | Y | Y | Y | Y |
| Redondo 2018 | Y | Y | N | Y | Y | Y | N | Y | Y | Y | Y |
| Redondo 2020a | Y | Y | Y | Y | Y | Y | Y | Y | Y | Y | Y |
| Redondo 2020b | Y | Y | Y | Y | Y | Y | Y | Y | Y | Y | Y |
| Reimelt 2021 | Y | Y | Y | Y | Y | Y | Y | Y | Y | Y | Y |
| Roessner 2008 | Y | Y | N | Y | Y | Y | N | Y | Y | Y | Y |
| Sánchez-Guillén 2020 | Y | Y | N | Y | Y | Y | N | Y | Y | Y | Y |
| Su 2018 | Y | Y | Y | Y | Y | Y | Y | Y | Y | Y | Y |
| Tunel 2021 | Y | Y | Y | Y | Y | Y | N | Y | Y | Y | Y |
| Uebel-von Sandersleben 2017 | Y | Y | Y | Y | Y | Y | N | Y | Y | Y | Y |
| Ulucan Atas 2020 | Y | Y | N | Y | Y | Y | N | Y | Y | Y | Y |
| Werner 2020 | Y | Y | Y | Y | Y | Y | Y | Y | Y | Y | Y |

(continue)

| | **Adequate description of basic data** | **Absence of non-response bias** | **Appropriate description of non-responders** | **Consistency of results** | **Clear presentation of results for all analyses described in methods** | **Justified discussions and conclusions** | **Discussion of limitations of the study** | **Absence of funding- or conflict of interest-related biases** | **Ethical approval and consent** |
|---|---|---|---|---|---|---|---|---|---|
| Ababneh 2020 | Y | N | N | Y | Y | Y | Y | Y | Y |
| Akmatov 2021 | Y | Y | Y | Y | Y | Y | Y | Y | Y |
| Aslan 2020 | Y | N | N | Y | Y | Y | Y | Y | Y |
| Ayyildiz & Ayyildiz 2019 | Y | N | N | Y | Y | Y | Y | Y | N |
| Bae 2019 | Y | N | N | Y | Y | Y | Y | Y | Y |
| Banaschewski 2006 | Y | N | N | Y | Y | Y | Y | N | Y |
| Bartgis 2009 | Y | N | N | Y | Y | Y | Y | Y | Y |
| Berger 2016 | Y | Y | Y | Y | Y | Y | Y | Y | Y |
| Bodur 2018 | Y | N | N | Y | Y | Y | N | N | Y |
| Brown 2020 | Y | N | N | Y | Y | Y | Y | Y | Y |
| Bubl 2013 | N | N | N | Y | Y | Y | Y | Y | Y |
| Bubl 2015 | Y | N | N | Y | Y | Y | Y | Y | Y |
| DeCarlo 2014 | Y | N | N | Y | Y | Y | Y | N | Y |
| DeCarlo 2016 | Y | Y | Y | Y | Y | Y | Y | N | NA |
| Donmez 2020 | Y | N | N | Y | Y | Y | Y | Y | Y |
| Fabian 2013 | Y | N | N | Y | Y | Y | Y | N | Y |
| Farrar 2001 | Y | N | N | Y | Y | Y | N | N | Y |
| Grönlund 2007 | N | N | N | Y | Y | Y | N | N | Y |
| Guvenmez 2020 | Y | N | N | Y | Y | Y | Y | Y | Y |
| Hergüner 2018 | Y | N | N | Y | Y | Y | Y | Y | Y |
| Ho 2020 | Y | Y | Y | Y | Y | Y | Y | Y | Y |
| Işik & Kaygisiz 2020 | Y | N | N | Y | Y | Y | Y | Y | Y |
| Karaca 2020 | Y | Y | N | Y | Y | Y | Y | Y | Y |
| Kim 2014a | Y | Y | N | Y | Y | Y | Y | Y | Y |
| Kim 2014b | Y | Y | N | Y | Y | Y | Y | Y | Y |

| | Adequate description of basic data | Absence of non-response bias | Appropriate description of non-responders | Consistency of results | Clear presentation of results for all analyses described in methods | Justified discussions and conclusions | Discussion of limitations of the study | Absence of funding- or conflict of interest-related biases | Ethical approval and consent |
|---|---|---|---|---|---|---|---|---|---|
| Kim 2014c | Y | Y | N | Y | Y | Y | Y | Y | Y |
| Kim 2015 | Y | Y | N | Y | Y | Y | Y | Y | Y |
| Kooij & Bijlenga 2014 | Y | Y | N | Y | Y | Y | Y | Y | Y |
| Martin 2008 | Y | Y | N | Y | Y | Y | N | Y | Y |
| McBride & Bijan 2017* | Y | Y | N | Y | Y | n/a | N | n/a | n/a |
| Merdler 2017 | Y | Y | N | Y | Y | Y | Y | Y | N |
| Mohney 2008 | Y | Y | N | Y | Y | Y | Y | Y | Y |
| Redondo 2018 | Y | Y | N | Y | Y | Y | Y | Y | Y |
| Redondo 2020a | Y | Y | N | Y | Y | Y | Y | Y | Y |
| Redondo 2020b | Y | Y | N | Y | Y | Y | Y | Y | Y |
| Reimelt 2021 | Y | Y | Y | Y | Y | Y | Y | Y | Y |
| Roessner 2008 | Y | Y | N | Y | Y | Y | Y | Y | Y |
| Sánchez-Guillén 2020 | Y | Y | N | Y | Y | Y | Y | Y | Y |
| Su 2018 | Y | Y | Y | Y | Y | Y | Y | Y | Y |
| Tunel 2021 | Y | Y | N | Y | Y | Y | Y | Y | Y |
| Uebel-von Sandersleben 2017 | Y | Y | N | Y | Y | Y | Y | Y | Y |
| Ulucan Atas 2020 | Y | Y | N | Y | Y | Y | Y | Y | Y |
| Werner 2020 | Y | Y | Y | Y | Y | Y | Y | Y | Y |

Based on: Downes, M.J.; Brennan, M.L.; Williams, H.C.; Dean, R.S. Development of a critical appraisal tool to assess the quality of cross-sectional studies (AXIS). BMJ Open 2016, 6, e011458, doi:10.1136/bmjopen-2016-011458.
Legend. Y: yes; N: no
*Conference abstract including relevant data on population study

**Supplement 6: Meta-analysis of studies investigating the prevalence of ADHD in people with and without vision problems**

*Table S2. Description of studies*

| Author, year | Vision problem | LogOdds [variance] |
|---|---|---|
| **Included in the meta-analysis** | | |
| **Author, year** | **Vision problem** | **LogOdds [variance]** |
| Berger, 2016 | Color Vision Deficiency | 2.4716 [0.0483] |
| DeCarlo, 2016 | Unspecified vision problems | 1.0433 [0.0063] |
| Mohney, 2008.1 | Strabismus | 1.4172 [0.1989] |
| Mohney, 2008.2 | Strabismus | 0.0577 [0.0577] |
| Su, 2018 | Strabismus | 0.4309 [0.0042] |
| **Included in the narrative review** | | |
| **Author, year** | **Vision problem** | **Descriptive summary of findings** |
| DeCarlo, 2016 | Unspecified vision problems | ADHD was more prevalent among children with vision problems vs normal vision. |
| Merdler, 2017 | Strabismus | Increased prevalence of ADHD in people with corrected strabismus. |

*Figure S1. Forest plot*

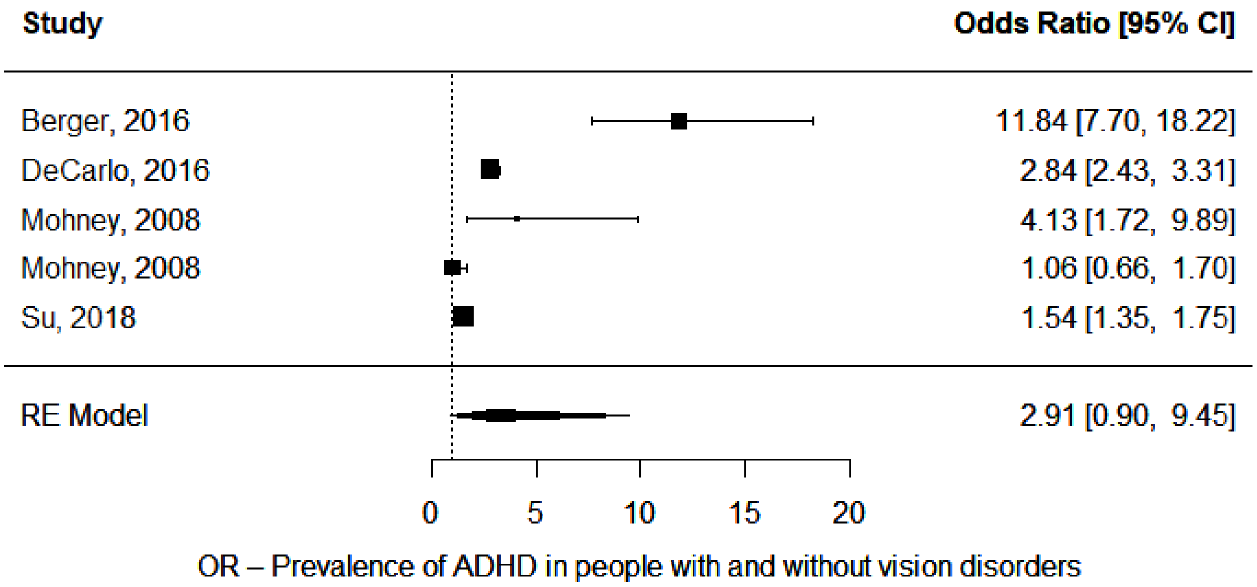


*RE: Random Effects; CI: Confidence Interval; OR: Odds Ratio*

***Figure S2. Funnel plots (a. no trim and fill analysis; b. trim and fill analysis excluding Mohney, 2008.2; c. trim and fill analysis excluding Mohney, 2008.1)***

a.

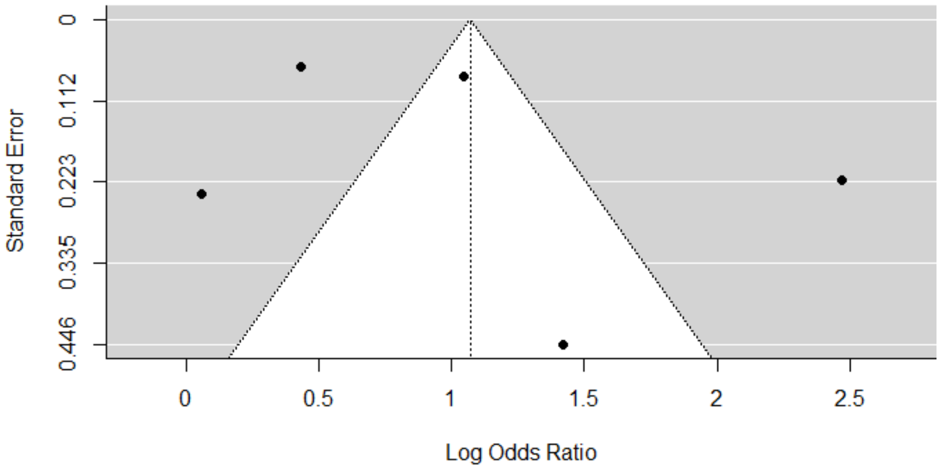


b.

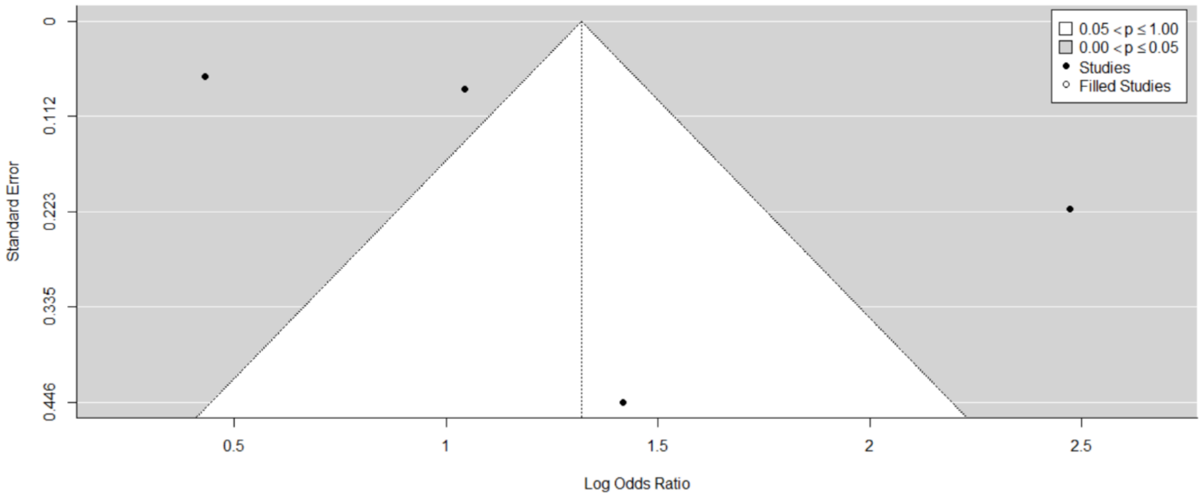


c.

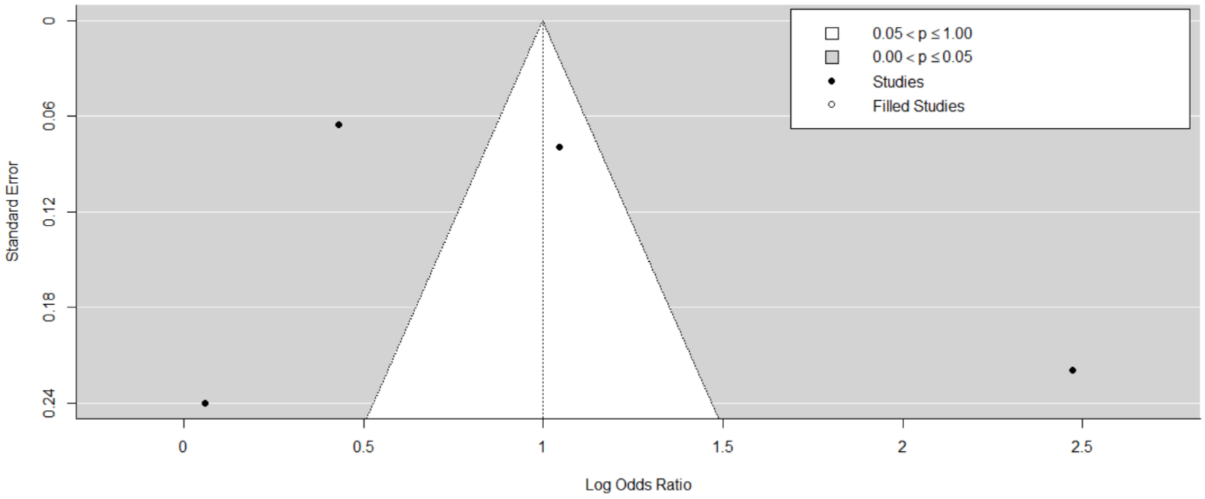

We carried out the meta-analysis only on studies on children and adolescents (i.e., excluding [21], which was on adults), and the model remained non-significant (logOR = 0.6840, SE = 0.2729, 95% CI = [-0.1843; 1.5524], t = 2.5069, $p = 0.0872$), with significant heterogeneity ($Q = 45.0488$; $p < 0.0001$) and no publication bias detected (Kendall's tau = 0.3333, $p = 0.7500$).

**Supplement 7: Meta-analyses of studies investigating the prevalence of vision problems/conditions in people with and without ADHD**

***a. Astigmatism***

***Table S3. Description of studies***

| **Included in the meta-analysis** | |
|---|---|
| **Author, year** | **LogOdds [variance]** |
| Grönlund, 2007 | 1.5884 [0.4859] |
| Ho, 2020 | 0.5397 [0.0142] |
| Karaca, 2020 | 0.3567 [0.9032] |
| Reimelt, 2021 | 0.6101 [0.0202] |

***Figure S3. Forest plot***

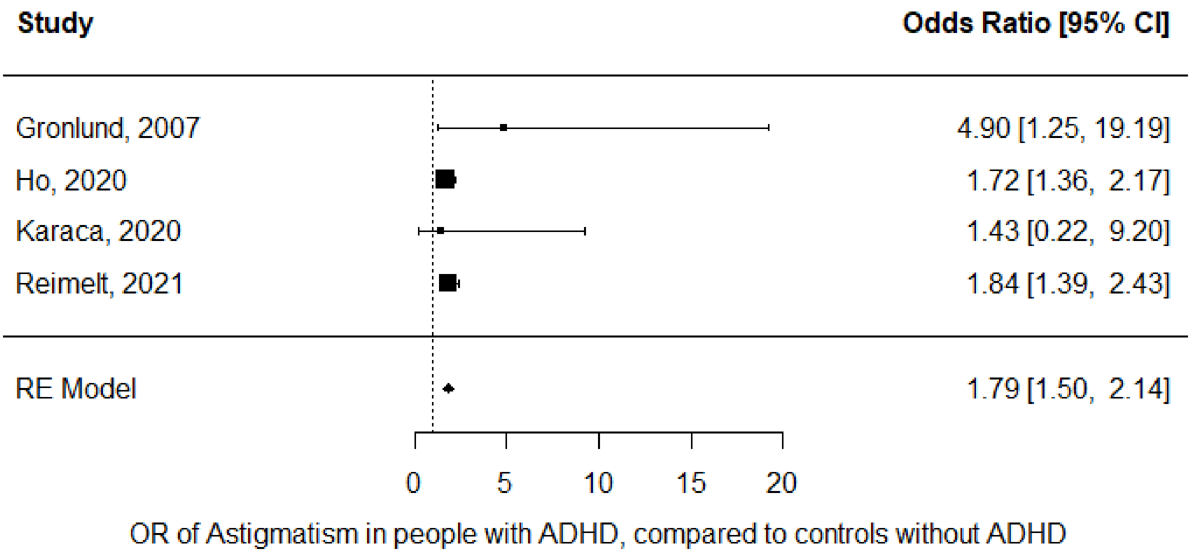


*RE: Random Effects; CI: Confidence Interval; OR: Odds Ratio*

**Test for Heterogeneity:** Q(df = 2) = 2.3058, p-val = 0.5114

***Figure S4. Funnel plots (a. no trim and fill analysis; b. trim and fill analysis)***

a.

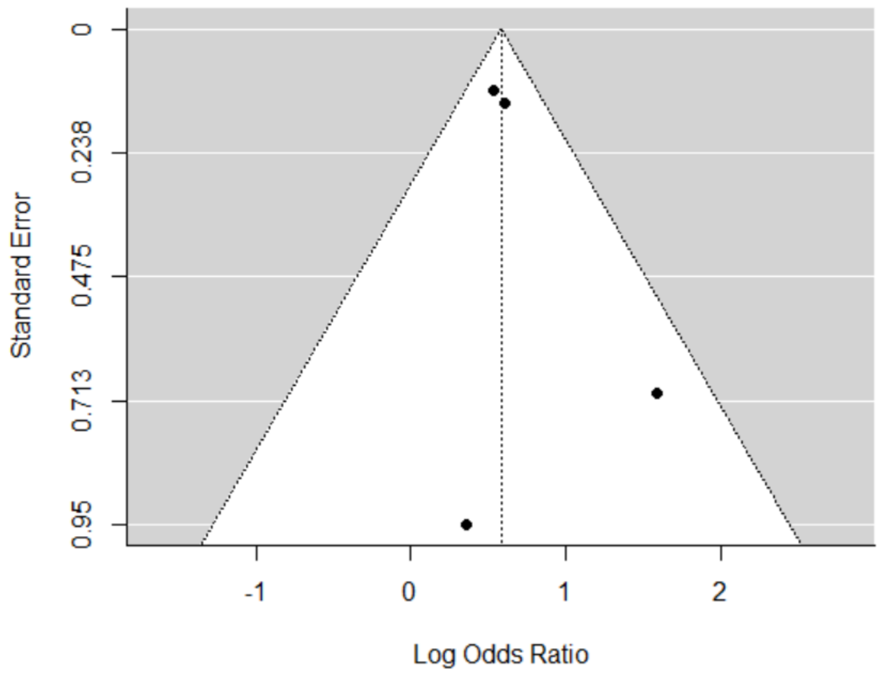


b.

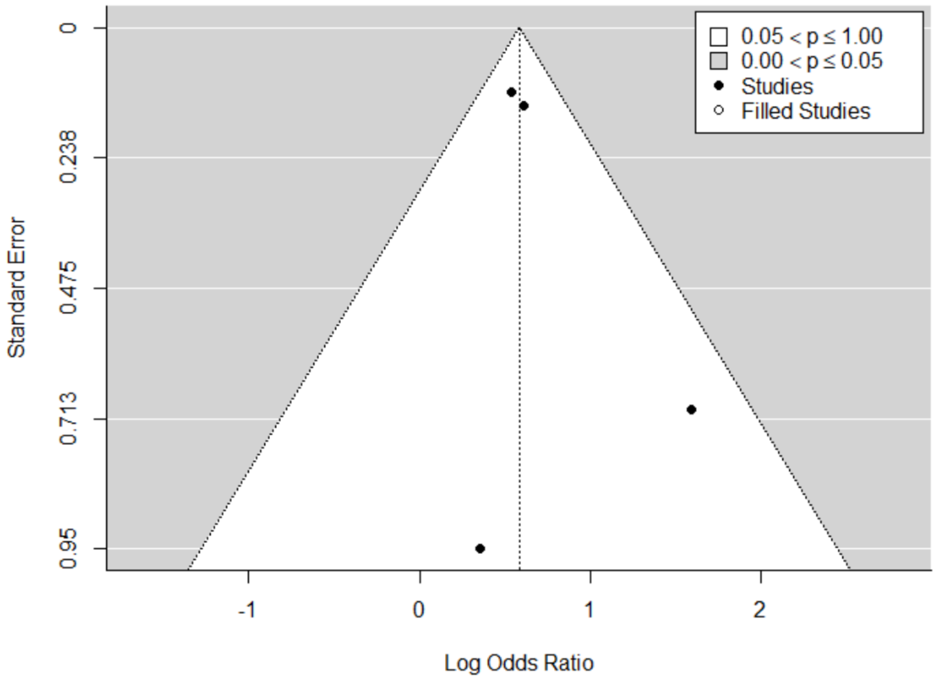

*b. Hyperopia and Hypermetropia*

*Table S4. Description of studies*

| **Included in the meta-analysis** | |
|---|---|
| **Author, year** | **LogOdds [variance]** |
| Ababneh, 2020.1 | 0.0728 [0.1457] |
| Ababneh, 2020.2 | 0.2915 [0.1463] |
| Grönlund, 2007 | 1.0341 [0.3535] |
| Ho, 2020 | 0.5884 [0.001] |
| Karaca, 2020 | 2.03 [0.3442] |
| Reimelt, 2021 | 0.515 [0.0174] |

*Figure S5. Forest plot*

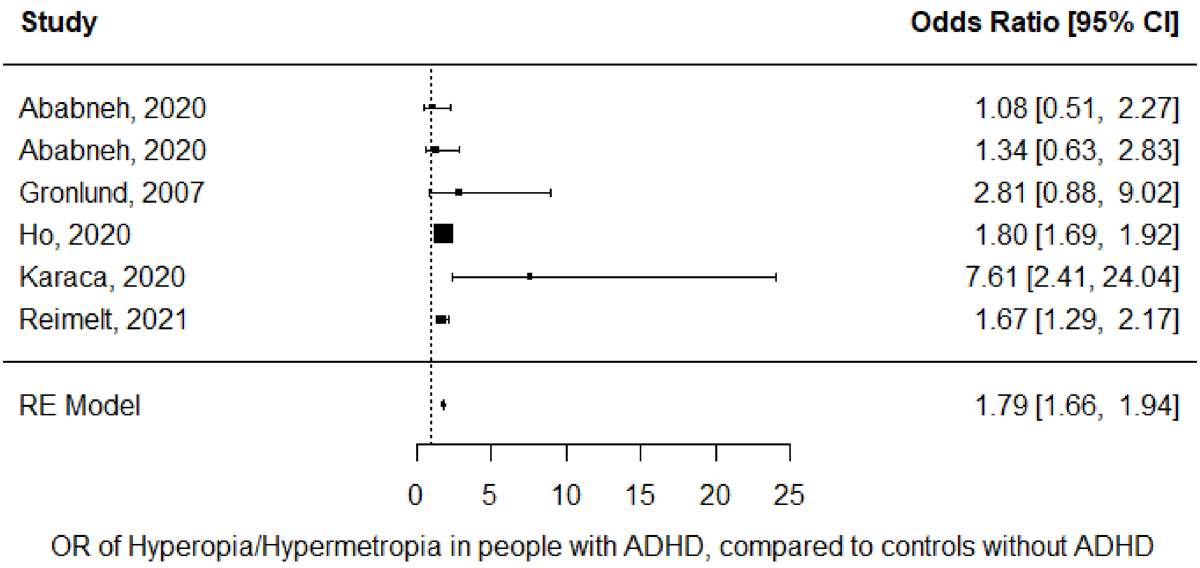


*RE: Random Effects; CI: Confidence Interval; OR: Odds Ratio*

**Test for Heterogeneity:** Q(df = 4) = 9.3200, p-val = 0.0970

*Figure S6. Funnel plots (a. no trim and fill analysis; b. trim and fill analysis excluding Ababneh, 2020.2; c. trim and fill analysis excluding Ababneh, 2020.1)*

**Rank Correlation Test for Funnel Plot Asymmetry:** Kendall's tau = 0.2000, p = 0.7194

a.

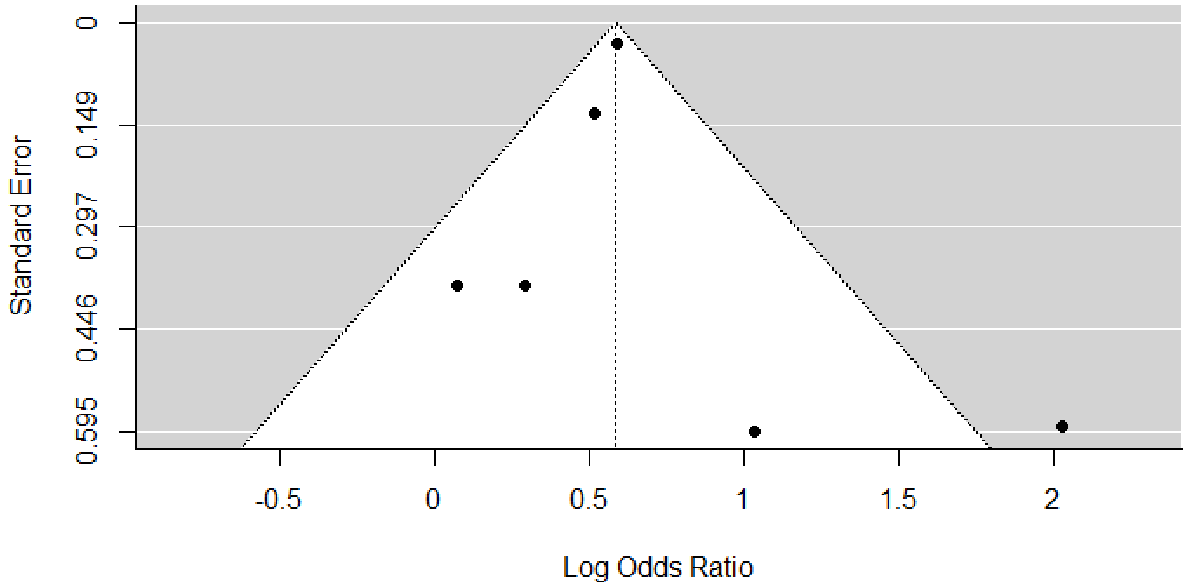


b.

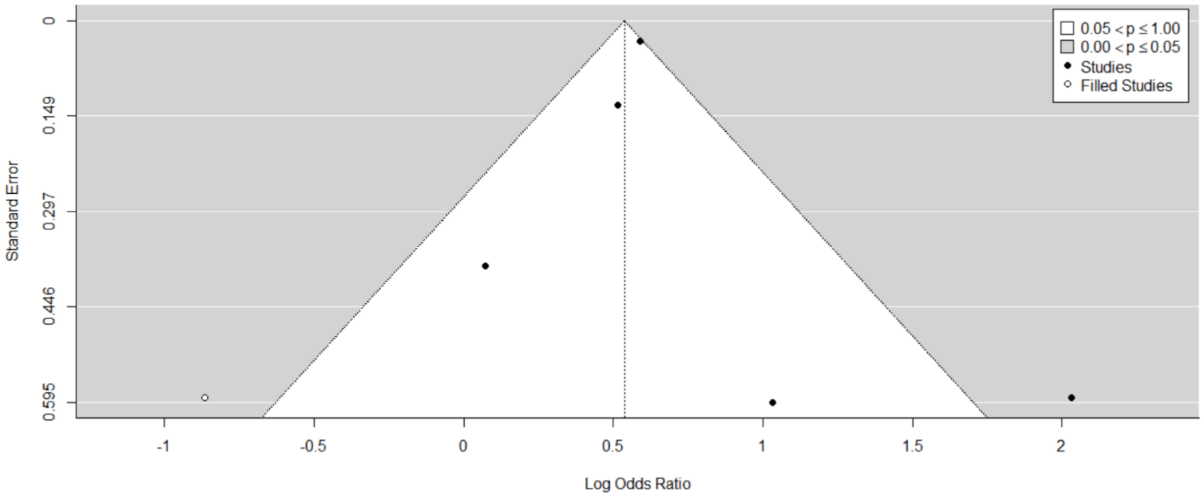


c.

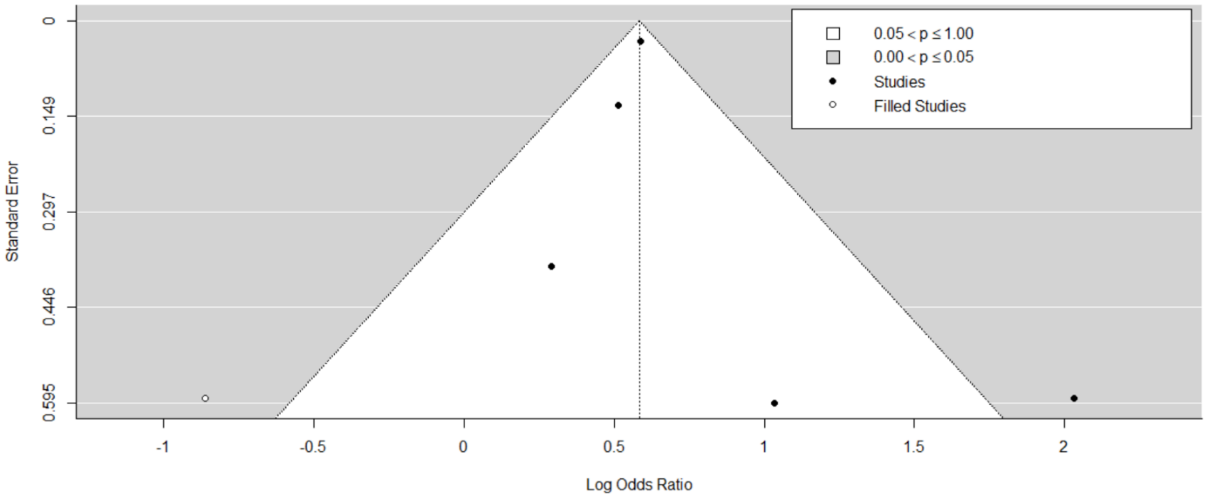

### *c. Myopia*

***Table S5. Description of studies***

| **Included in the meta-analysis** | |
|---|---|
| **Author, year** | **LogOdds [variance]** |
| Ababneh, 2020.1 | -0.7985 [0.3422] |
| Ababneh, 2020.2 | -0.7526 [0.2644] |
| Grönlund, 2007 | 0.3684 [0.3205] |
| Karaca, 2020 | -1.1451 [1.2359] |
| Reimelt, 2021 | 0.2512 [0.0139] |

***Figure S7. Forest plot***

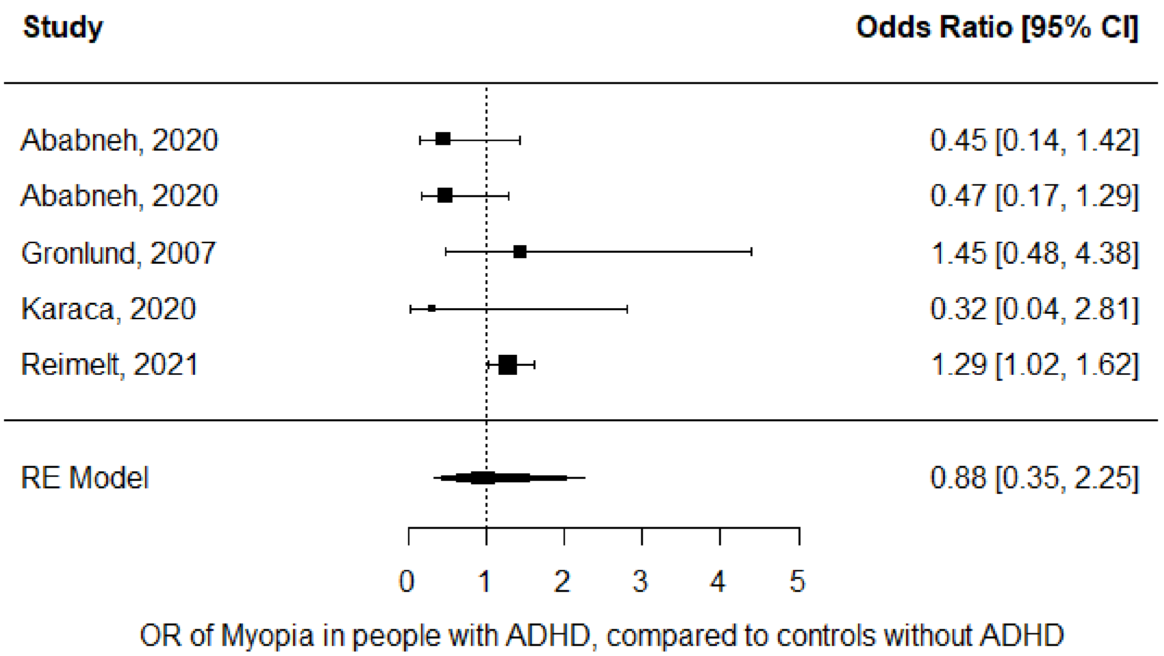


*RE: Random Effects; CI: Confidence Interval; OR: Odds Ratio*

**Test for Heterogeneity:** Q(df = 3) = 7.9475, p-val = 0.0935

*Figure S8. Funnel plots (a. no trim and fill analysis; b. trim and fill analysis excluding Ababneh, 2020.2; c. trim and fill analysis excluding Ababneh, 2020.1)*

**Rank Correlation Test for Funnel Plot Asymmetry:** Kendall's tau = -0.2000, p = 0.8167

a.

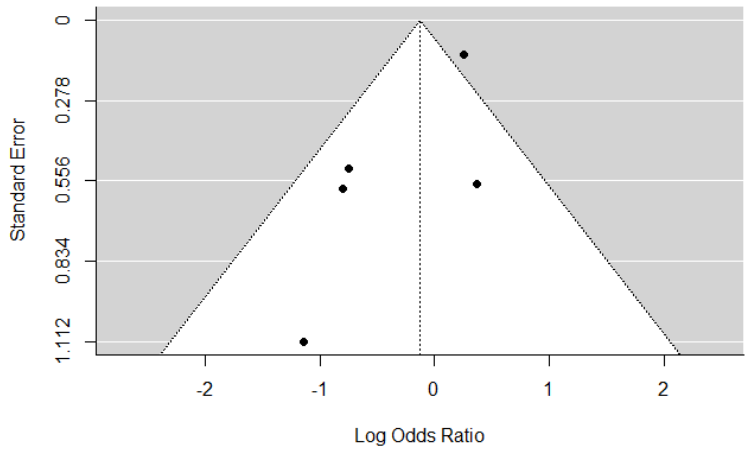


b.

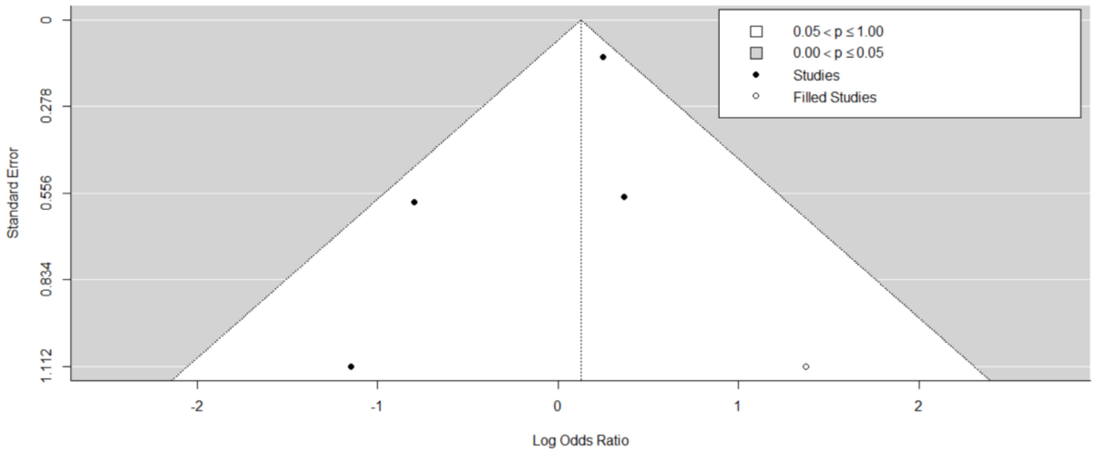


c.

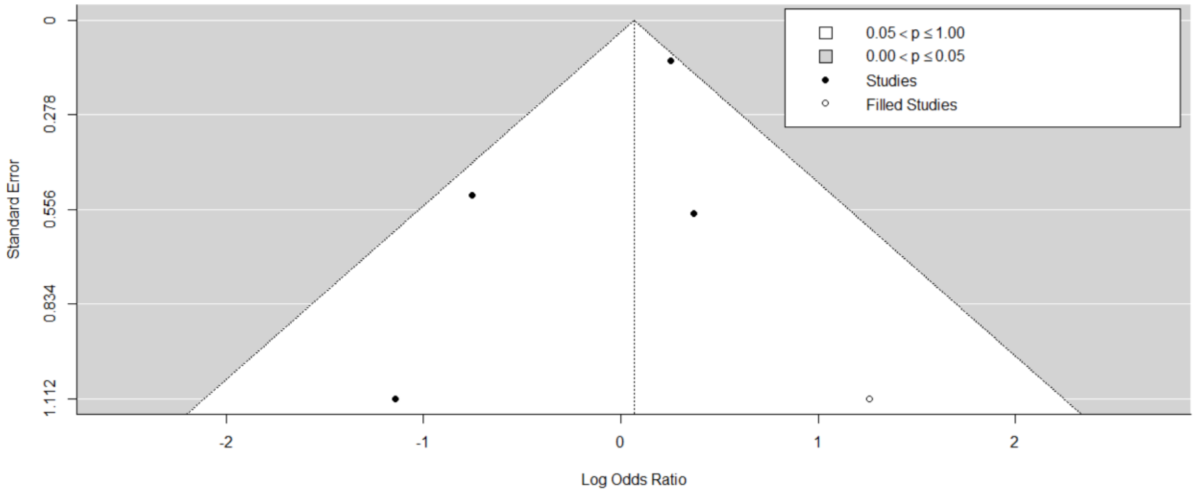

*d. Reduced near point of convergence*

*Table S6. Description of studies*

| **Included in the meta-analysis** | |
|---|---|
| **Author, year** | **LogOdds [variance]** |
| Ababneh, 2020 | 1.6457 [0.6517] |
| Grönlund, 2007 | 1.5884 [0.4859] |
| **Included in the narrative review** | |
| **Author, year** | **Descriptive summary of findings** |
| Fabian, 2013 | No significant difference in prevalence of convergence insufficiency in children with and without ADHD. |

*Figure S9. Forest plot*

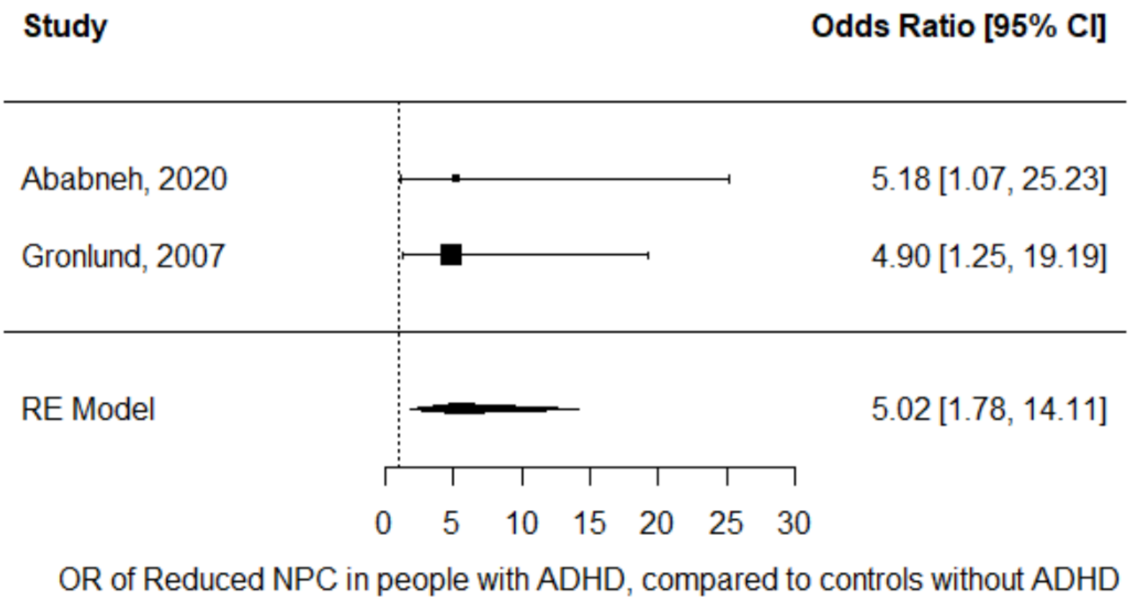


*RE: Random Effects; CI: Confidence Interval; OR: Odds Ratio; NPC: Near Point of Convergence*

**Test for Heterogeneity:** Q(df = 1) = 0.0029, p-val = 0.9571

***Figure S10. Funnel plot***

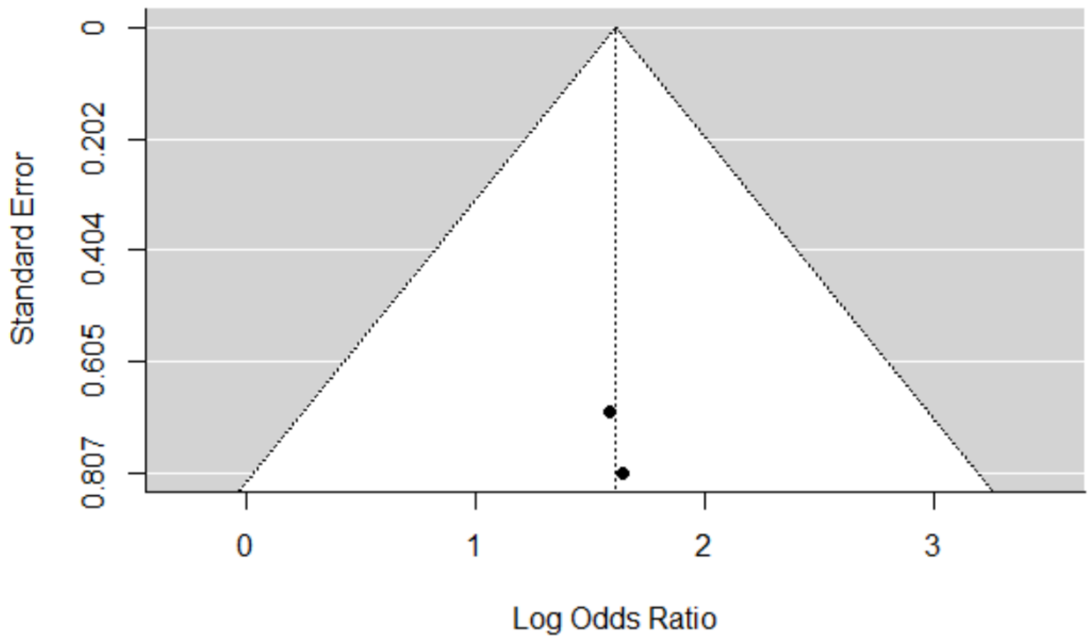

*e. Strabismus*

*Table S7. Description of studies*

| **Included in the meta-analysis** | |
|---|---|
| **Author, year** | **LogOdds [variance]** |
| Grönlund, 2007 | 1.2809 [0.3389] |
| Ho, 2020.1 | 0.6278 [0.0015] |
| Ho, 2020.2 | 0.6904 [0.0024] |
| Reimelt, 2021 | 0.7149 [0.0253] |
| **Included in the narrative review** | |
| **Author, year** | **Descriptive summary of findings** |
| Grönlund, 2007 | Significantly increased prevalence of heterophoria in ADHD |
| Fabian, 2013 | No significant differences in heterophoria at distance between children with and without ADHD |

*Figure S11. Forest plot*

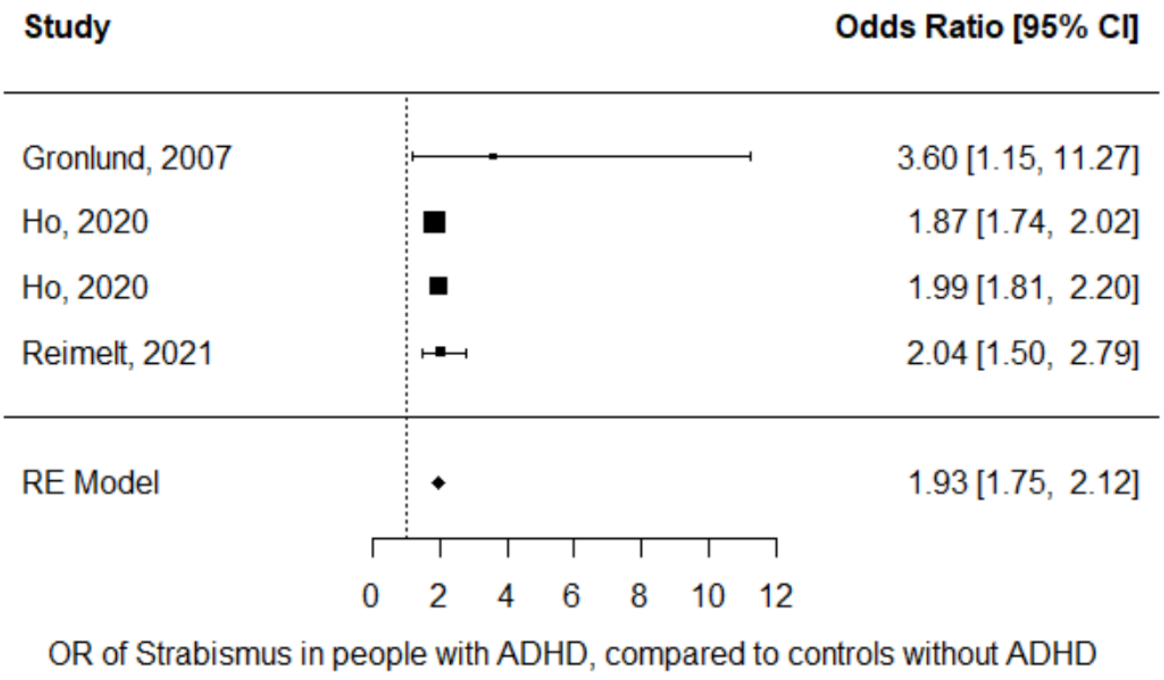


*RE: Random Effects; CI: Confidence Interval; OR: Odds Ratio*

**Test for Heterogeneity:** Q(df = 3) = 2.3025, p-val = 0.5120

***Figure S12. Funnel plots (a. no trim and fill analysis; b. trim and fill analysis excluding Ho, 2020.2; c. trim and fill analysis excluding Ho, 2020.1)***

**Rank Correlation Test for Funnel Plot Asymmetry:** Kendall's tau = 0.6667, p = 0.3333

a.

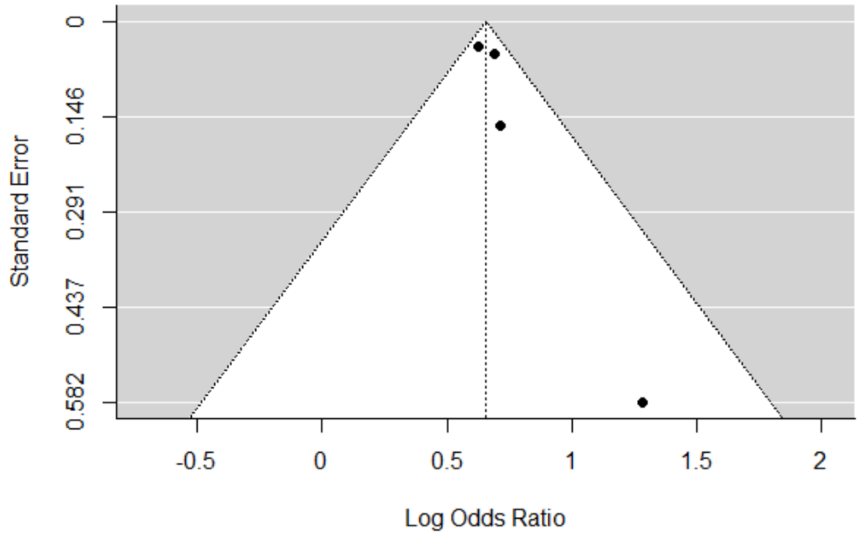


b.

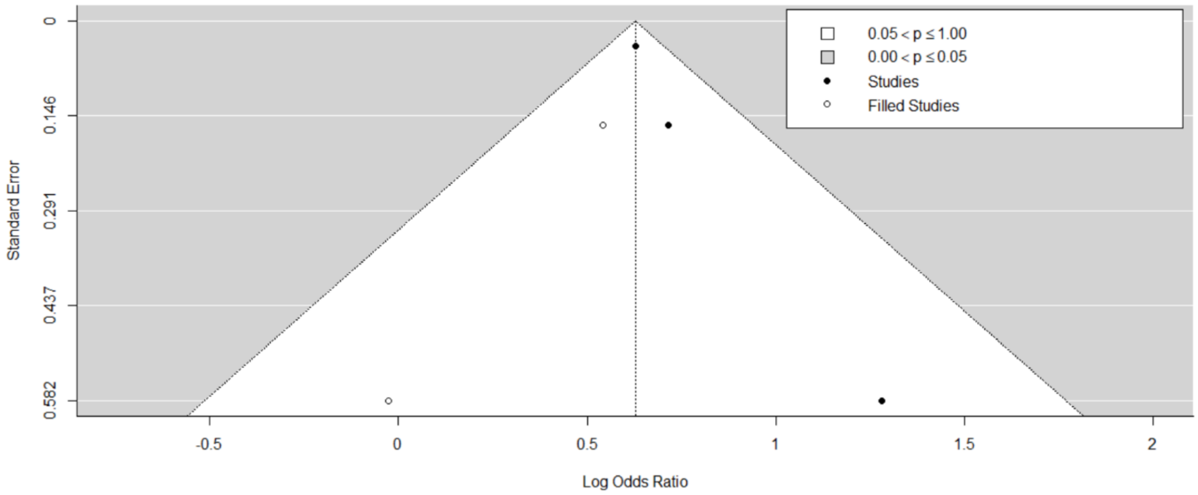


c.

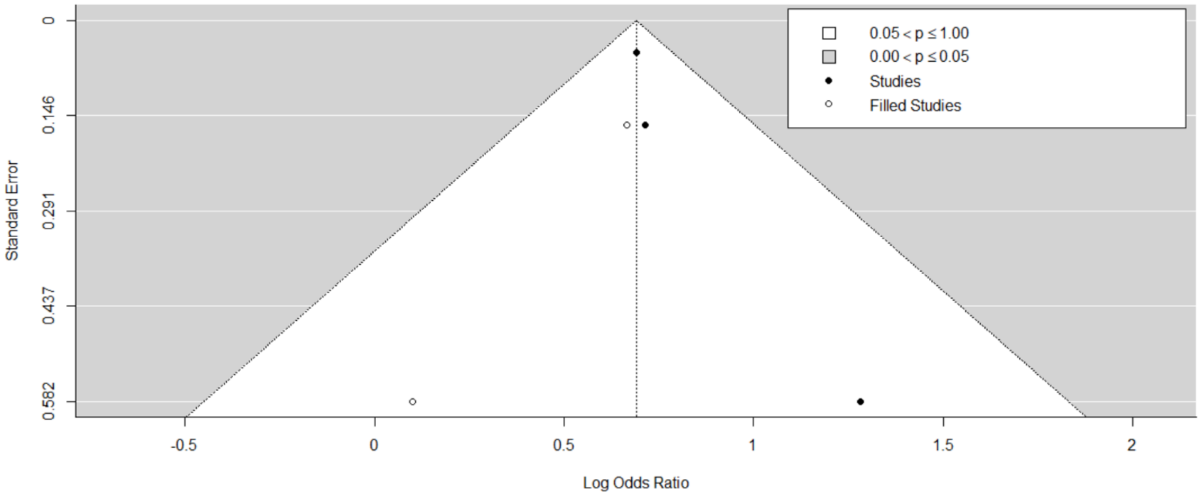

*f. Unspecified Vision problems*

***Table S8. Description of studies***

| **Included in the meta-analysis** | |
|---|---|
| **Author, year** | **LogOdds [variance]** |
| Akmatov, 2021 | 0.707 [<0.0001] |
| Grönlund, 2007.1 | 2.5925 [1.1618] |
| Grönlund, 2007.2 | 1.0129 [0.2775] |
| Kooij, 2014.1 | 0.3538 [0.0432] |
| Kooij, 2014.2 | 0.6082 [0.1653] |

***Figure S13. Forest plot***

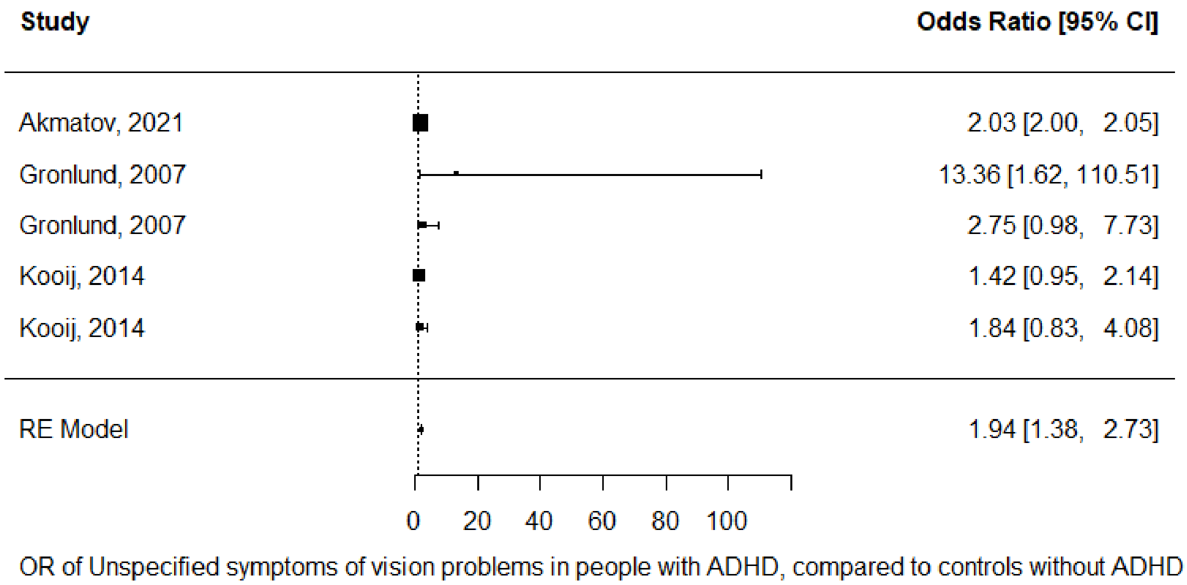


*RE: Random Effects; CI: Confidence Interval; OR: Odds Ratio*

**Test for Heterogeneity:** Q(df = 3) =6.3402, p-val = 0.1751

***Figure S14. Funnel plots (a. no trim and fill analysis; b. trim and fill analysis excluding Grönlund, 2007.2 and Kooij, 2014.2; c. trim and fill analysis excluding Grönlund, 2007.1 and Kooij, 2014.1; d. trim and fill analysis excluding Grönlund, 2007.2 and Kooij, 2014.1; e. trim and fill analysis excluding Grönlund, 2007.1 and Kooij, 2014.2)***

**Rank Correlation Test for Funnel Plot Asymmetry**: Kendall's tau = 0.4000, p = 0.4833

a.

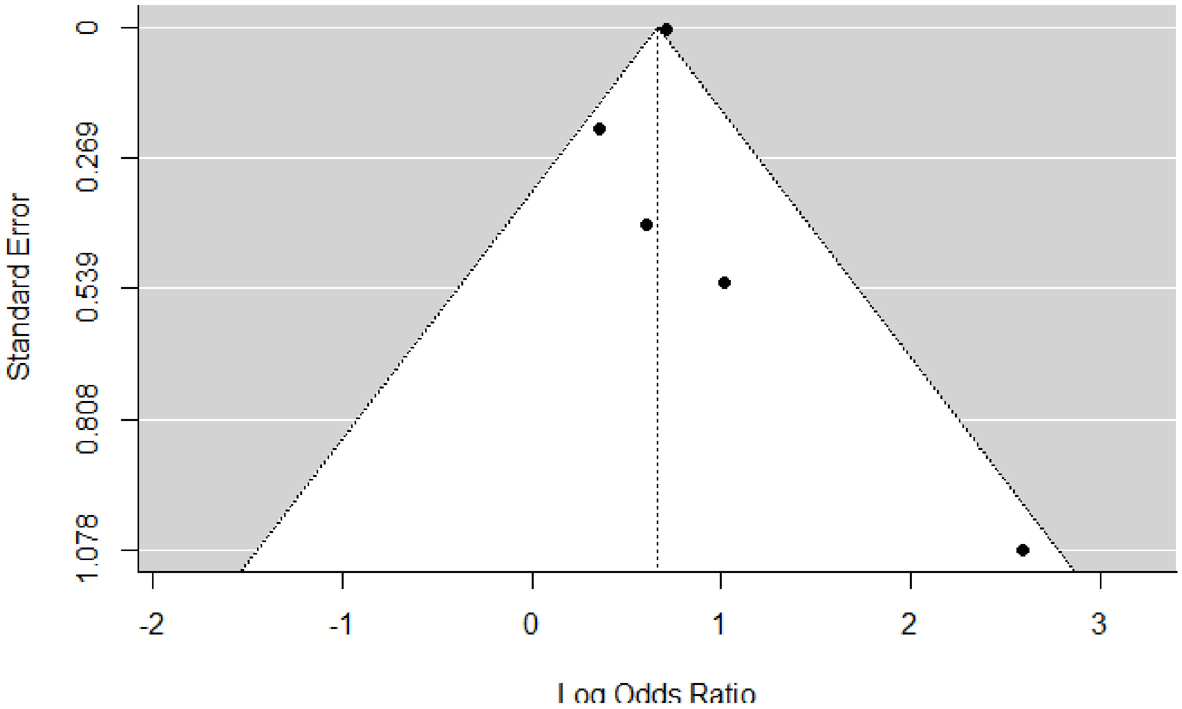


b.

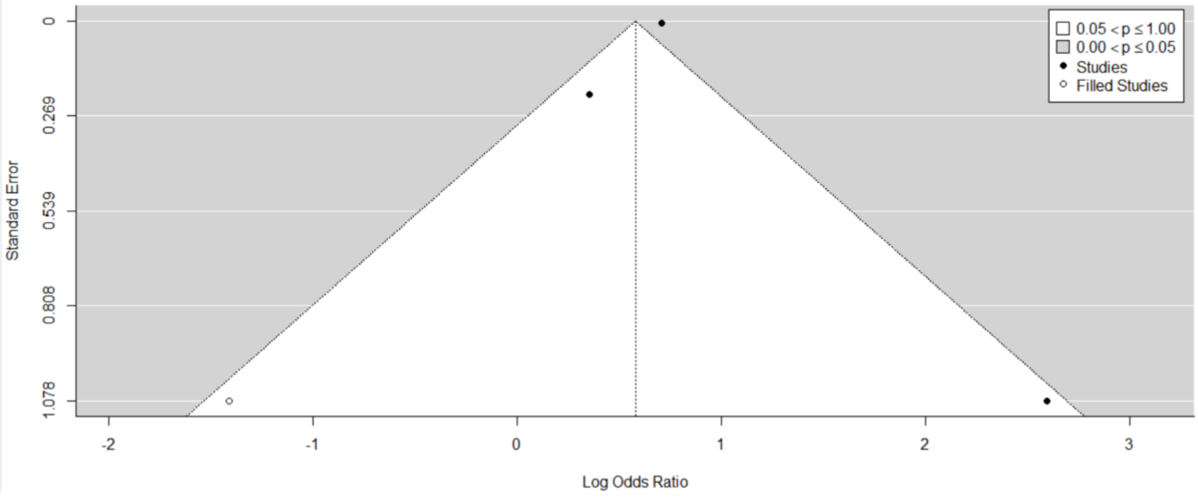

c.

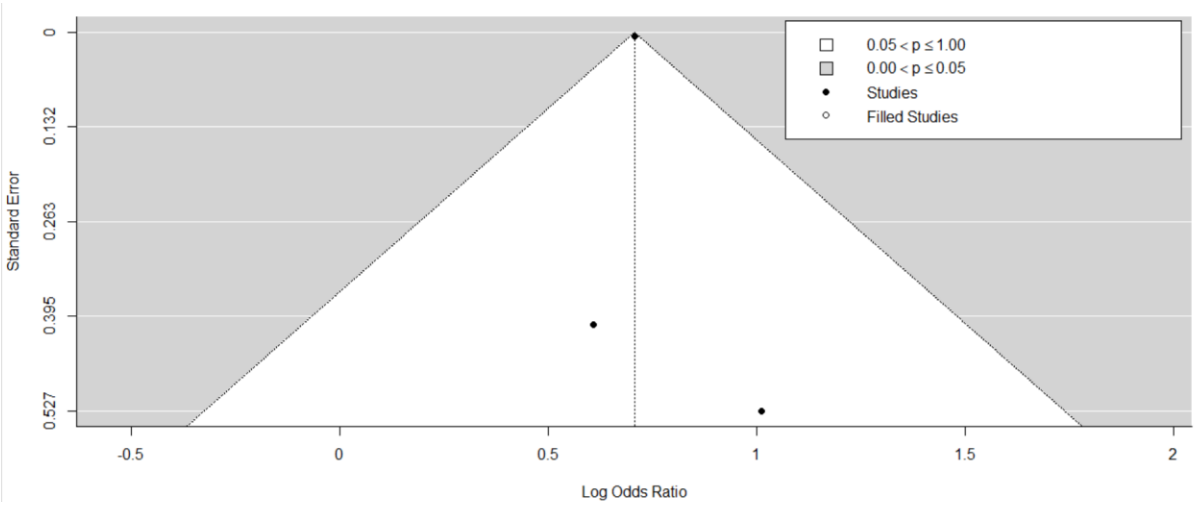
0.05 < p ≤ 1.00
0.00 < p ≤ 0.05
Studies
Filled Studies
Standard Error
0
0.132
0.263
0.395
0.527
-0.5
0
0.5
1
1.5
2
Log Odds Ratio

d.

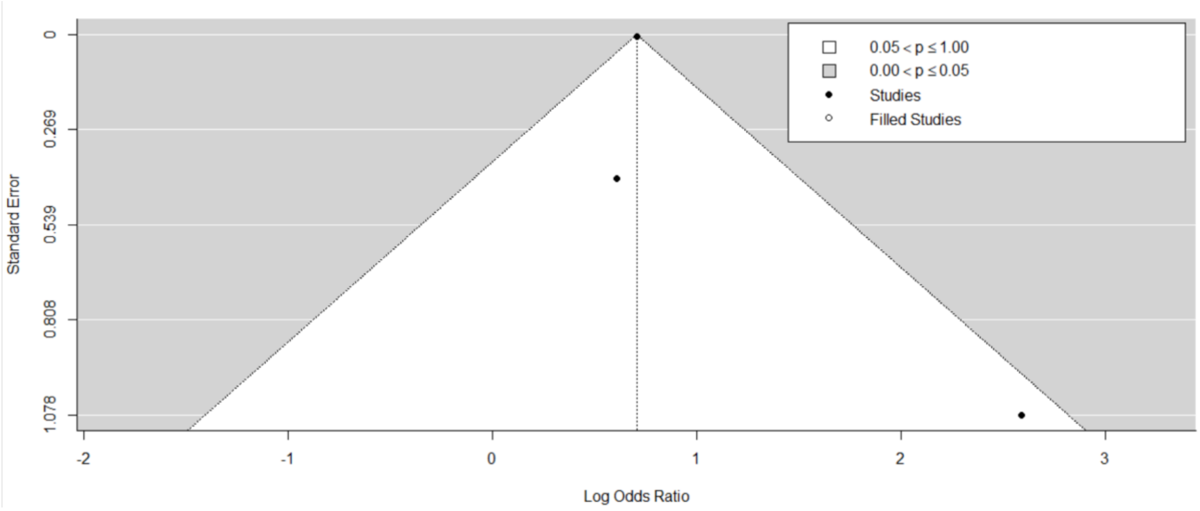
0.05 < p ≤ 1.00
0.00 < p ≤ 0.05
Studies
Filled Studies
Standard Error
0
0.269
0.539
0.808
1.078
-2
-1
0
1
2
3
Log Odds Ratio

e.

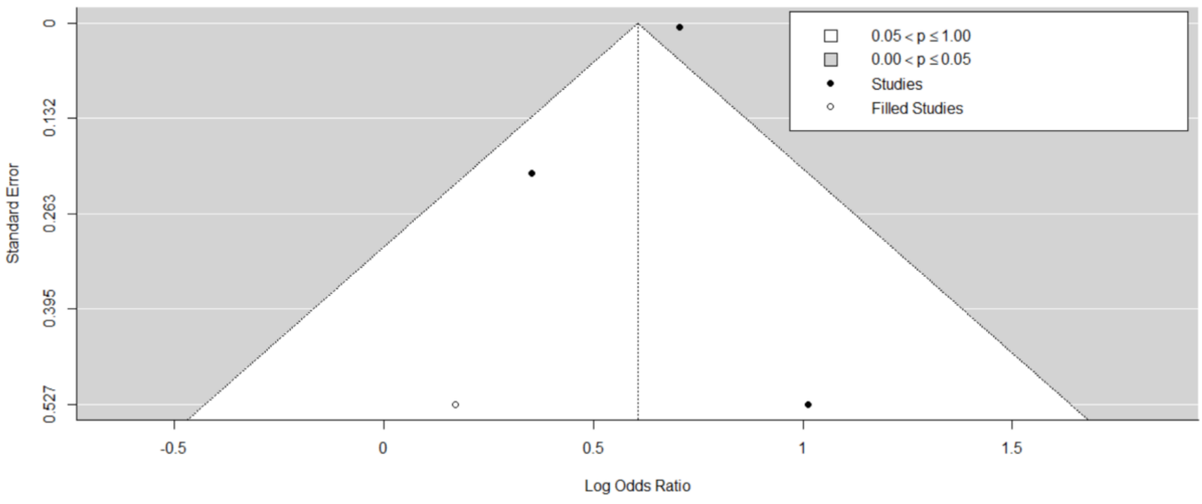
0.05 < p ≤ 1.00
0.00 < p ≤ 0.05
Studies
Filled Studies
Standard Error
0
0.132
0.263
0.395
0.527
-0.5
0
0.5
1
1.5
Log Odds Ratio

**Supplement 8: Results, forest and funnel plots for studies investigating the differences on anatomic (measurable) measures**

*a. Axial Length (of the eye)*

*Table S9. Description of studies*

| Included in the meta-analysis | |
|---|---|
| **Author, year** | **Hedge's g [variance]** |
| Ayyildiz, 2019 | 0.7592 [0.0715] |
| Grönlund, 2007 | -0.4769 [0.045] |

*Figure S15. Forest plot*

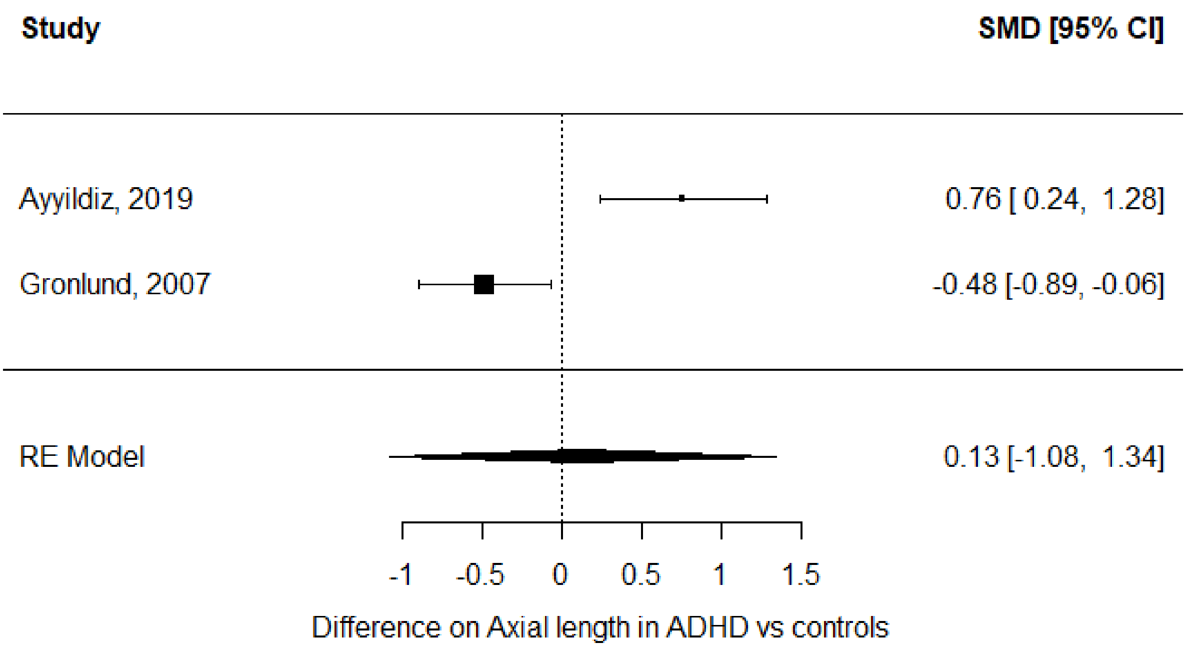


*RE: Random Effects; CI: Confidence Interval; SMD: Standardized Mean Difference*

**Test for Heterogeneity:** Q(df = 1) = 13.1137, p-val = 0.0003

***Figure S16. Funnel plot***

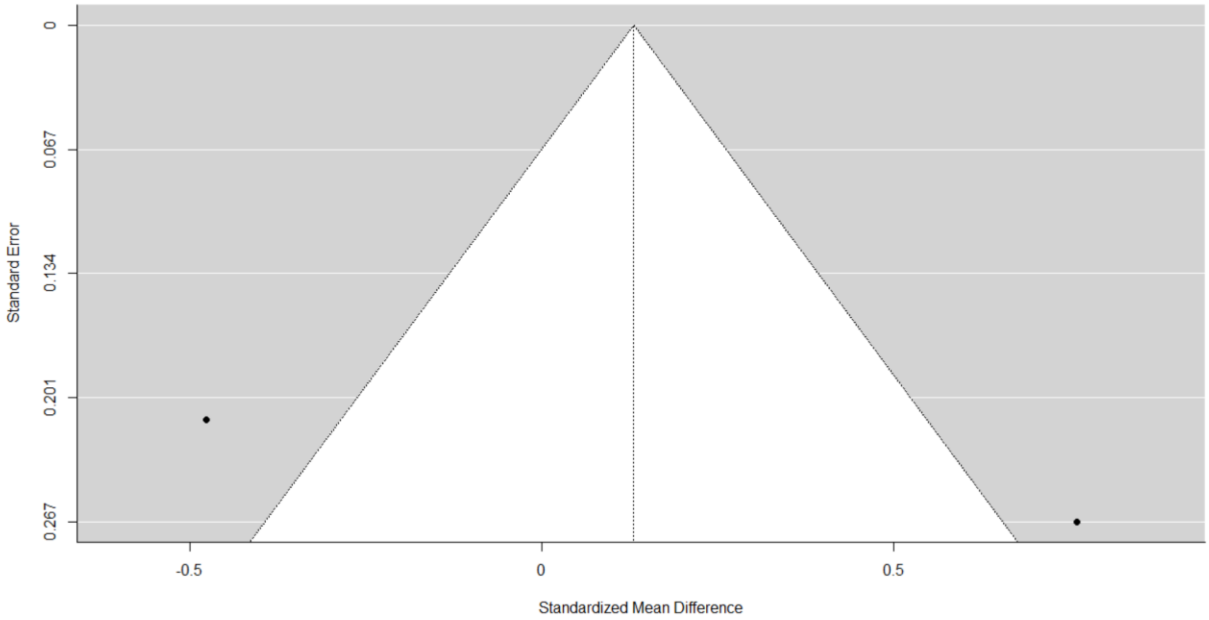

***b. Ganglion Cell Layer Thickness***

***Table S10. Description of studies***

| **Included in the meta-analysis** | |
|---|---|
| **Author, year** | **Hedge's g [variance]** |
| Bodur, 2018 | -0.9475 [0.0718] |
| Isik, 2020.1 | 0.1023 [0.04] |
| Isik, 2020.2 | 0.0756 [0.045] |
| Sánchez-Guillén, 2020 | -0.0164 [0.087] |
| Tunel, 2021 | -0.6623 [0.0811] |
| **Included in the narrative review** | |
| **Author, year** | **Descriptive summary of findings** |
| Ulucan Atas, 2020 | No significant difference between children with and without ADHD on macular ganglion cell complex thickness |

***Figure S17. Forest plot***

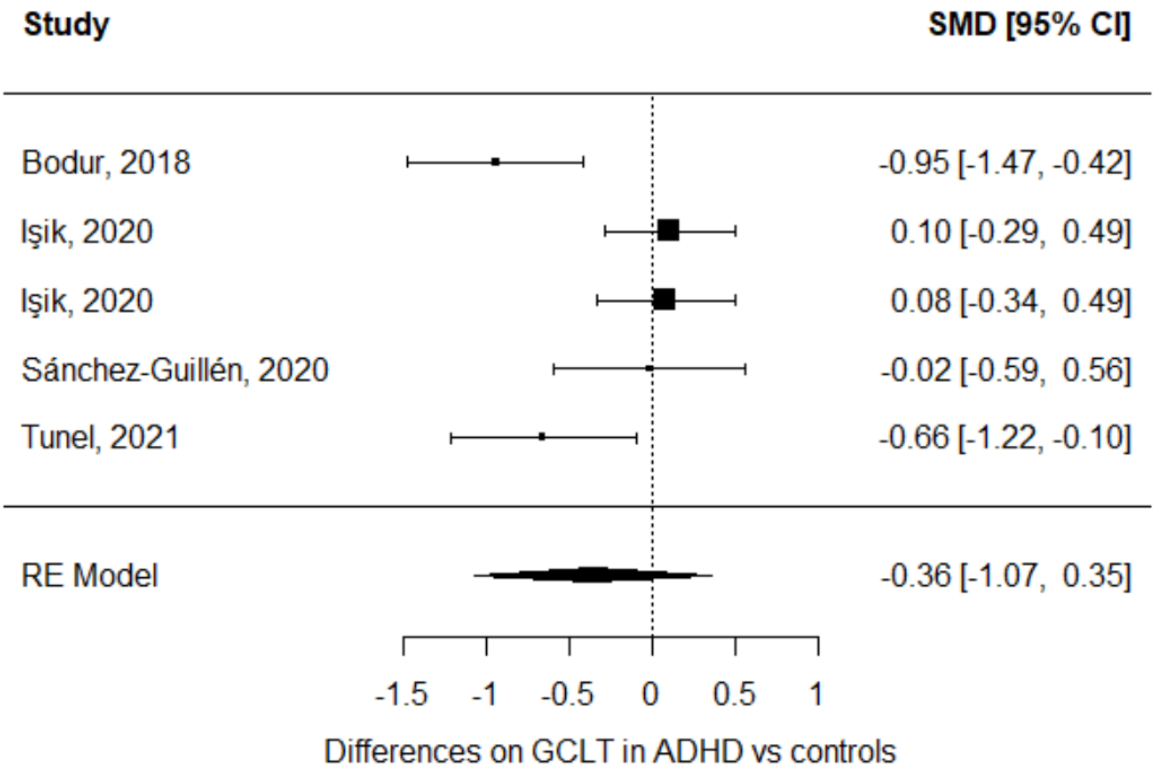


*RE: Random Effects; CI: Confidence Interval; SMD: Standardized Mean Difference; GCLT: Ganglion Cell Layer Thickness*

**Test for Heterogeneity:** Q(df = 4) = 14.7801, p-val = 0.0052

***Figure S18. Funnel plots (a. no trim and fill analysis; b. trim and fill analysis excluding Isik, 2020.2; c. trim and fill analysis excluding Isik, 2020.1)***

**Rank Correlation Test for Funnel Plot Asymmetry:** Kendall's tau = -0.4000, p = 0.4833

a.

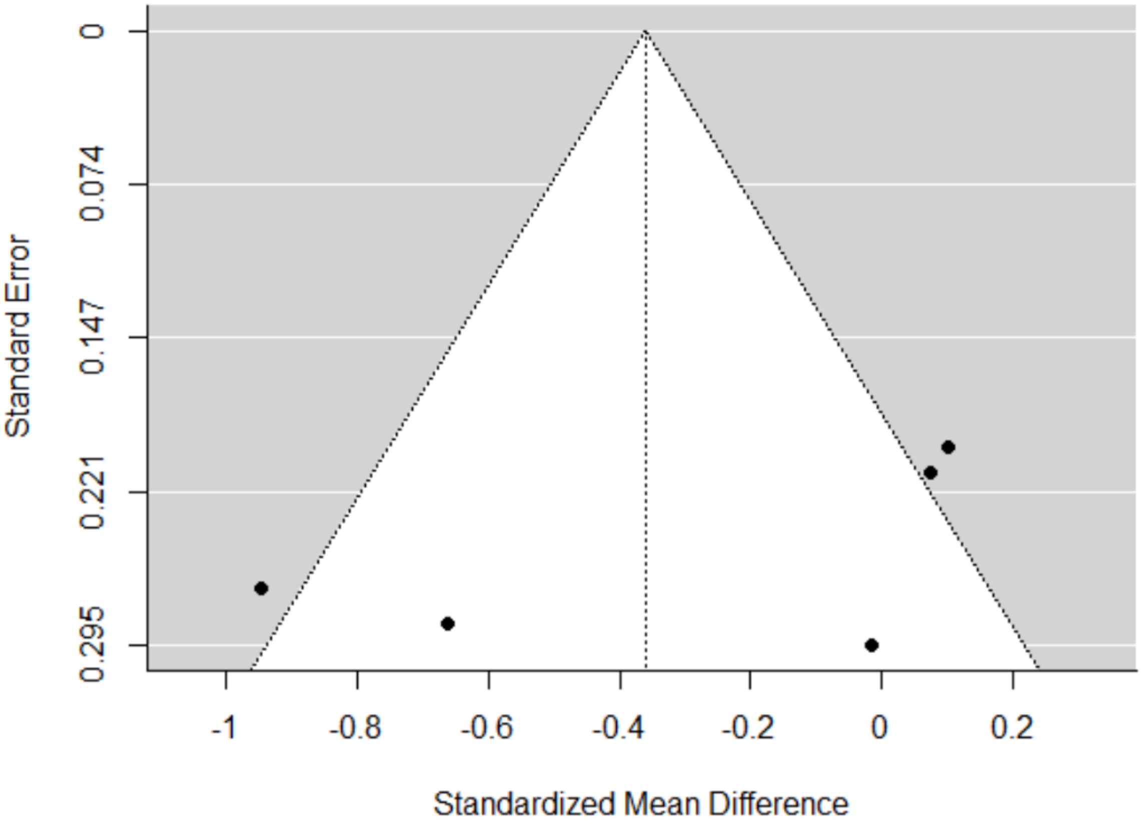


b.

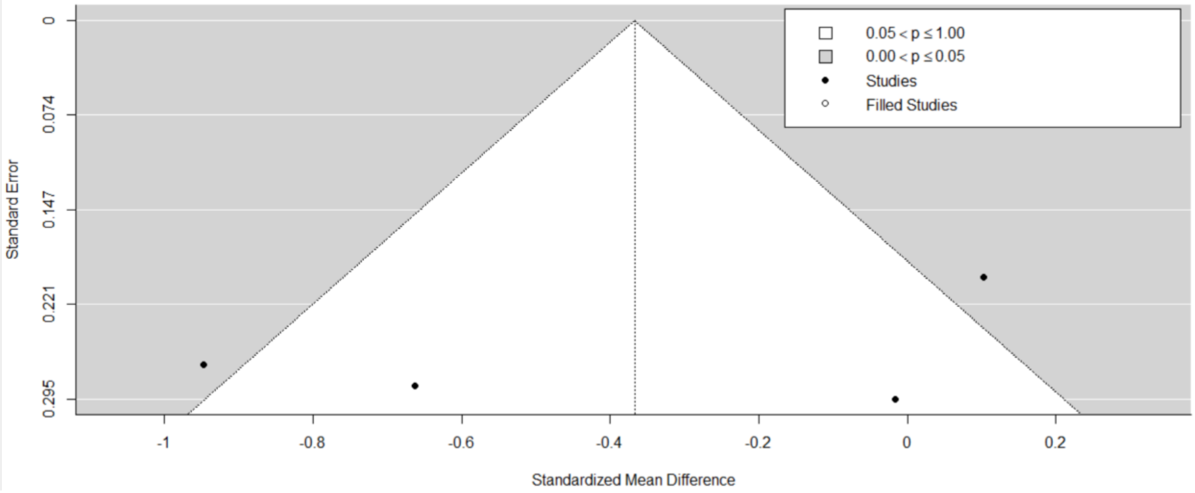

c.

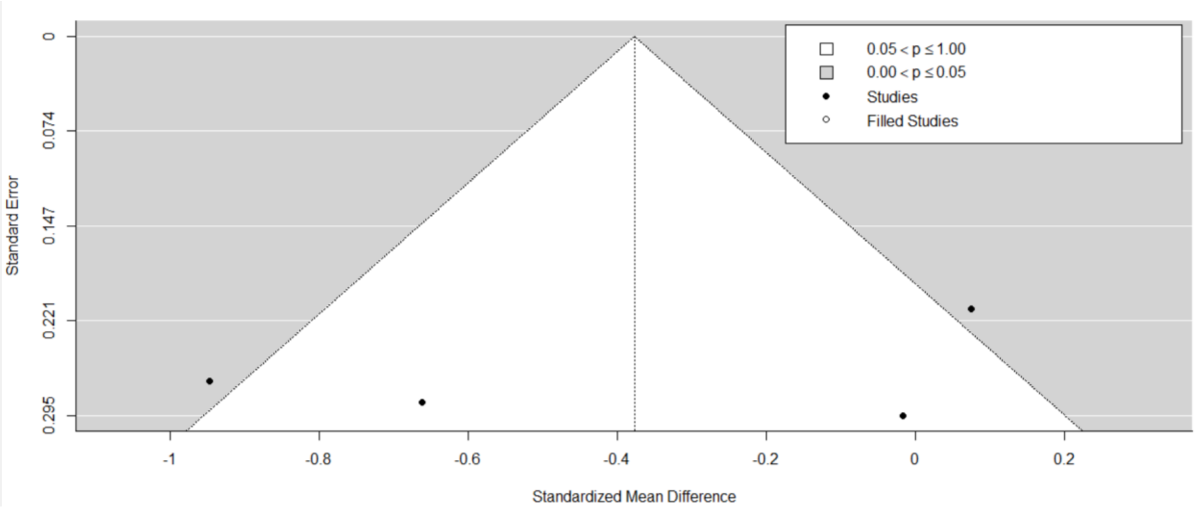


The results did not change when we only included studies on children and adolescents (i.e., excluding [58]) (Hedge's g = -0.2736, SE = 0.3271, 95% CI = -1.3146; 0.7674], t = -0.8364, p = 0.4643, significant heterogeneity: Q = 11.7499; p = 0.0083, publication bias not detected: Kendall's tau = -0.6667, p = 0.3333).

*c. Intraocular pressure*

*Table S11. Description of studies*

| **Included in the meta-analysis** | |
|---|---|
| **Author, year** | **Hedge's g [variance]** |
| Bae, 2019 | 0.2923 [0.162] |
| Guvenmez, 2020.1 | -0.4883 [0.0543] |
| Guvenmez, 2020.2 | -0.3033 [0.0522] |
| Isik, 2020.1 | 0.2626 [0.0403] |
| Isik, 2020.2 | 0.4813 [0.0463] |

*Figure S19. Forest plot*

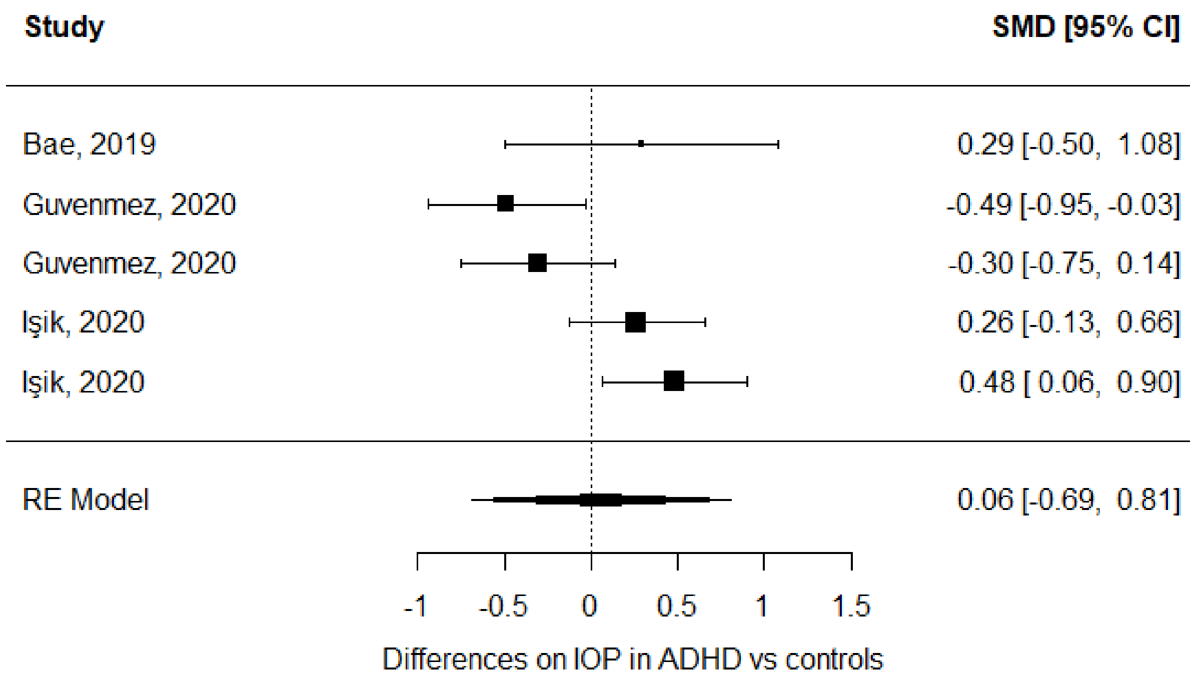


*RE: Random Effects; CI: Confidence Interval; SMD: Standardized Mean Difference; IOP: Intraocular Pressure*

**Test for Heterogeneity:** Q(df = 4) = 13.2278, p-val = 0.0102

*Figure S20. Funnel plots (a. no trim and fill analysis; b. trim and fill analysis excluding Guvenmez, 2020.2 and Isik, 2014.2; c. trim and fill analysis excluding Guvenmez, 2007.1 and Isik, 2014.1; d. trim and fill analysis excluding Guvenmez, 2007.2 and Isik, 2014.1; e. trim and fill analysis excluding Guvenmez, 2007.1 and Isik, 2014.2)*

**Rank Correlation Test for Funnel Plot Asymmetry:** Kendall's tau = -0.4000, p = 0.4833

a.

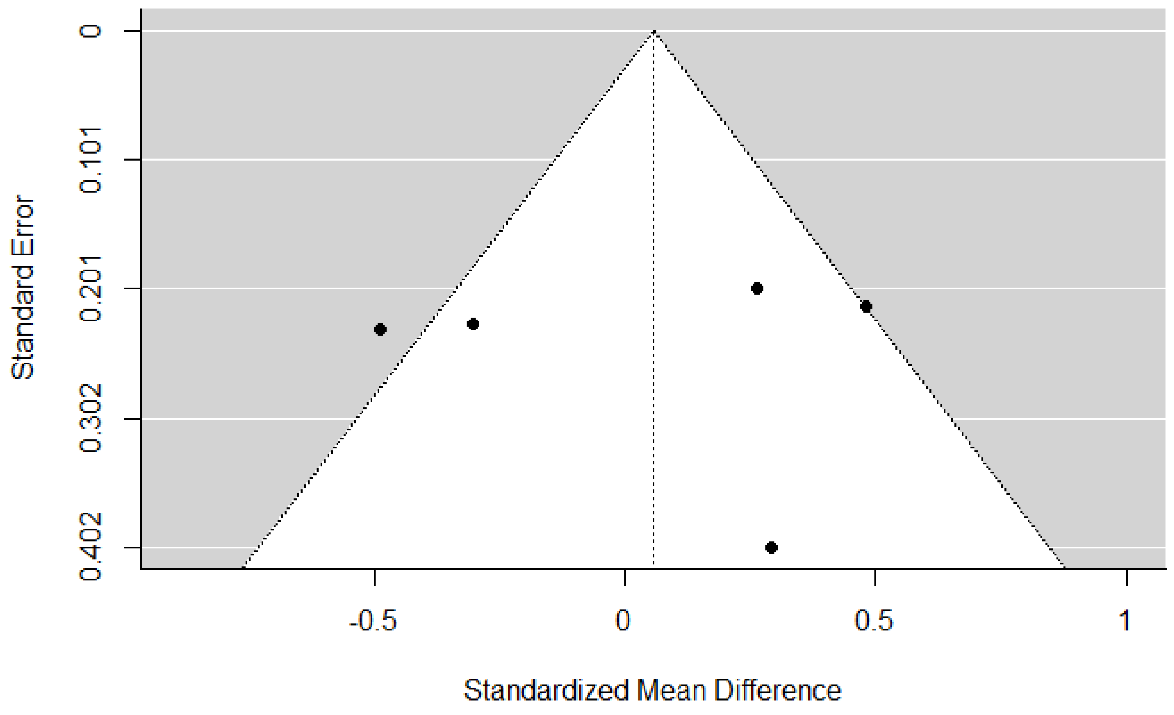


b.

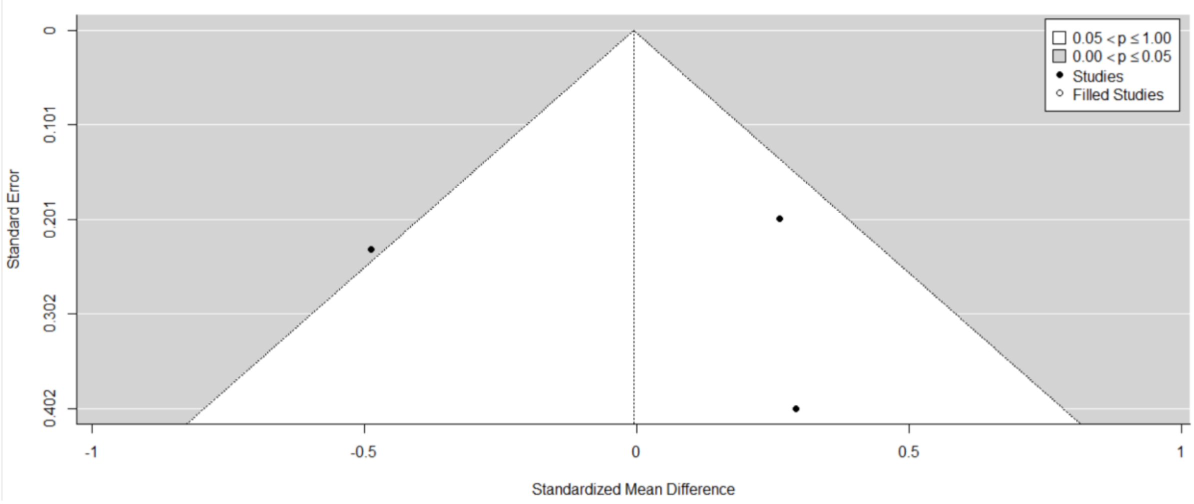

c.

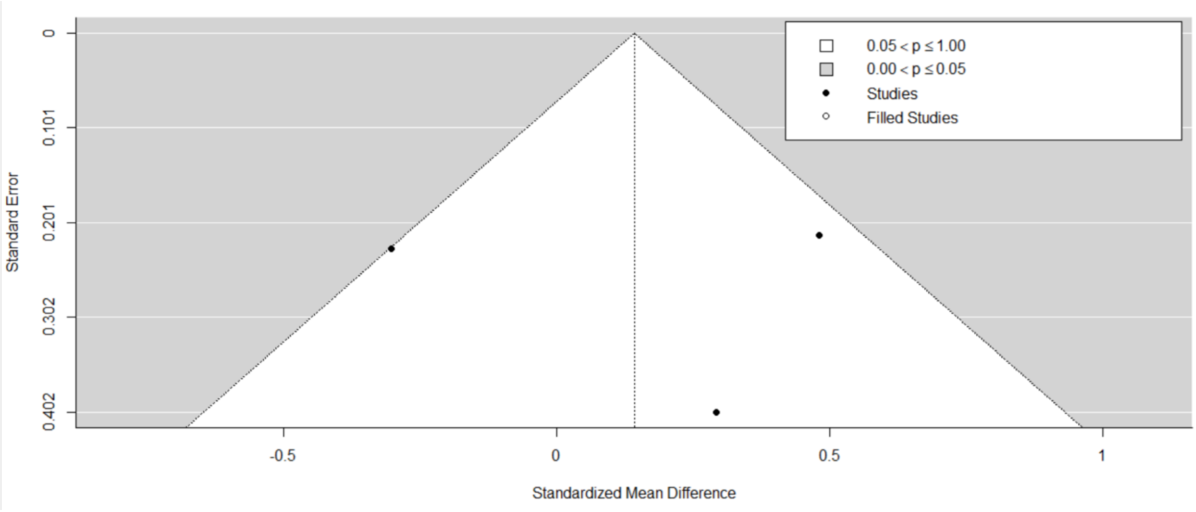
0.05 < p ≤ 1.00
0.00 < p ≤ 0.05
Studies
Filled Studies
Standard Error
0
0.101
0.201
0.302
0.402
-0.5
0
0.5
1
Standardized Mean Difference

d.

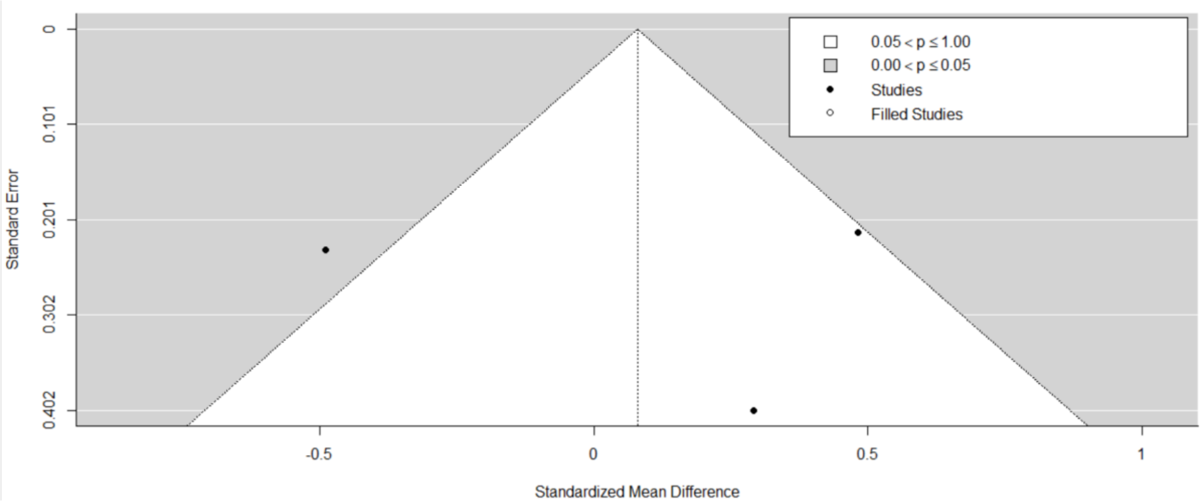
0.05 < p ≤ 1.00
0.00 < p ≤ 0.05
Studies
Filled Studies
Standard Error
0
0.101
0.201
0.302
0.402
-0.5
0
0.5
1
Standardized Mean Difference

e.

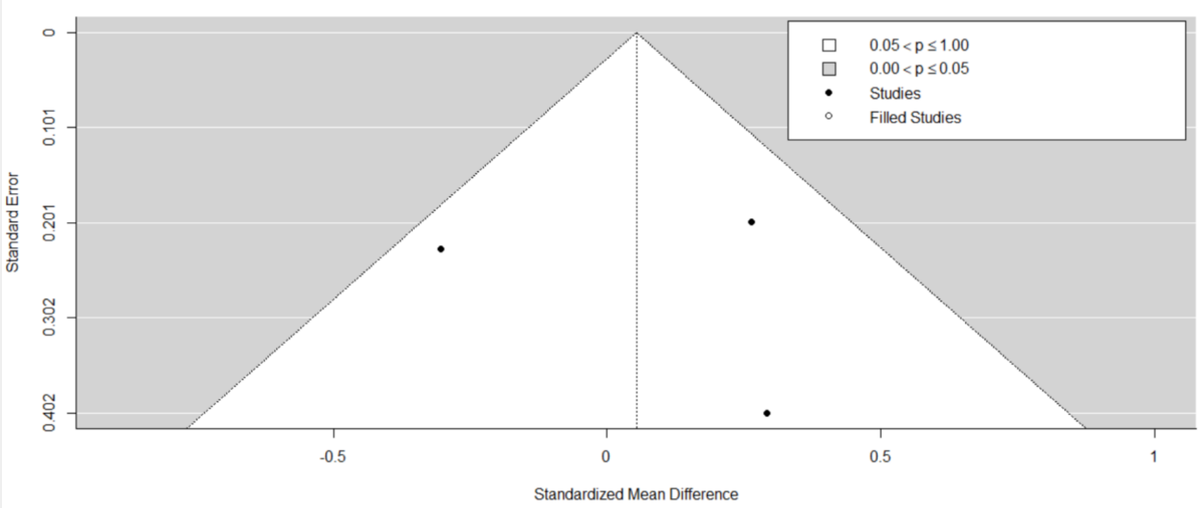
0.05 < p ≤ 1.00
0.00 < p ≤ 0.05
Studies
Filled Studies
Standard Error
0
0.101
0.201
0.302
0.402
-0.5
0
0.5
1
Standardized Mean Difference

***d. Macular Thickness***

***Table S12. Description of studies***

| **Included in the meta-analysis** | |
|---|---|
| **Author, year** | **Hedge's g [variance]** |
| Aslan, 2020 | -0.2684 [0.055] |
| Ayyildiz, 2019 | 0.0026 [0.0667] |
| Bae, 2019.1 | 0.076 [0.1604] |
| Bae, 2019.2 | 0.8416 [0.1744] |
| Bae, 2019.3 | 0.8696 [0.1754] |
| Herguner, 2018.1 | 0.0824 [0.0445] |
| Herguner, 2018.2 | -0.0068 [0.0444] |
| Işik, 2020 | 0.0319 [0.04] |
| Sánchez-Guillén, 2020.1 | -0.4963 [0.0896] |
| Sánchez-Guillén, 2020.2 | -0.3075 [0.088] |
| Sánchez-Guillén, 2020.3 | -1.7786 [0.1213] |
| Tunel, 2021.1 | -0.5129 [0.0795] |
| Tunel, 2021.2 | -0.6294 [0.0807] |
| Tunel, 2021.3 | -0.6673 [0.0812] |
| Ulucan Atas, 2020 | -0.6784 [0.0572] |
| **Included in the narrative review** | |
| **Author, year** | **Descriptive summary of findings** |
| Ababneh, 2020 | Similar prevalence of abnormal central foveal thickness in people with and without ADHD |
| Bodur, 2018 | Reduced optical nerve thickness in children with ADHD vs without |
| Ayyildiz, 2019 | Increased corneal thickness in children with ADHD vs without |

*Figure S21. Forest plot*

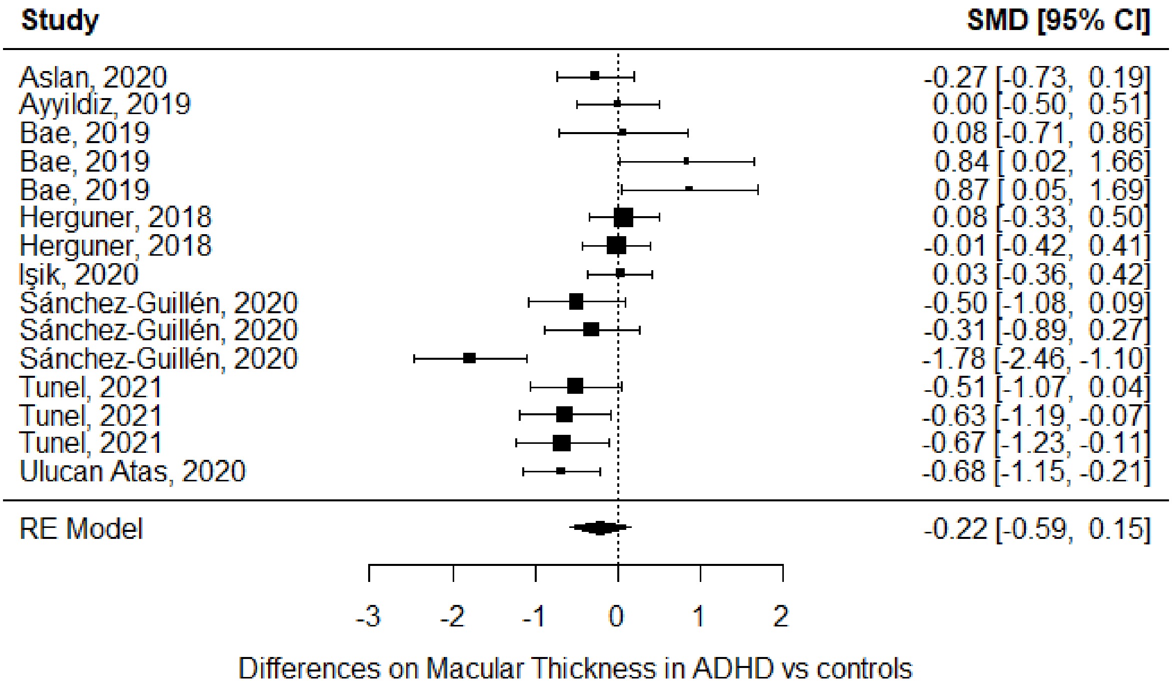


*RE: Random Effects; CI: Confidence Interval; SMD: Standardized Mean Difference*

**Test for Heterogeneity:** Q(df = 14) = 49.3732, p-val < .0001

*Figure S22. Funnel plots (a. no trim and fill analysis; b. trim and fill analysis excluding largest effect sizes for each study reporting more than one effect size)*

**Rank Correlation Test for Funnel Plot Asymmetry:** Kendall's tau = -0.0476, p = 0.8458

a.

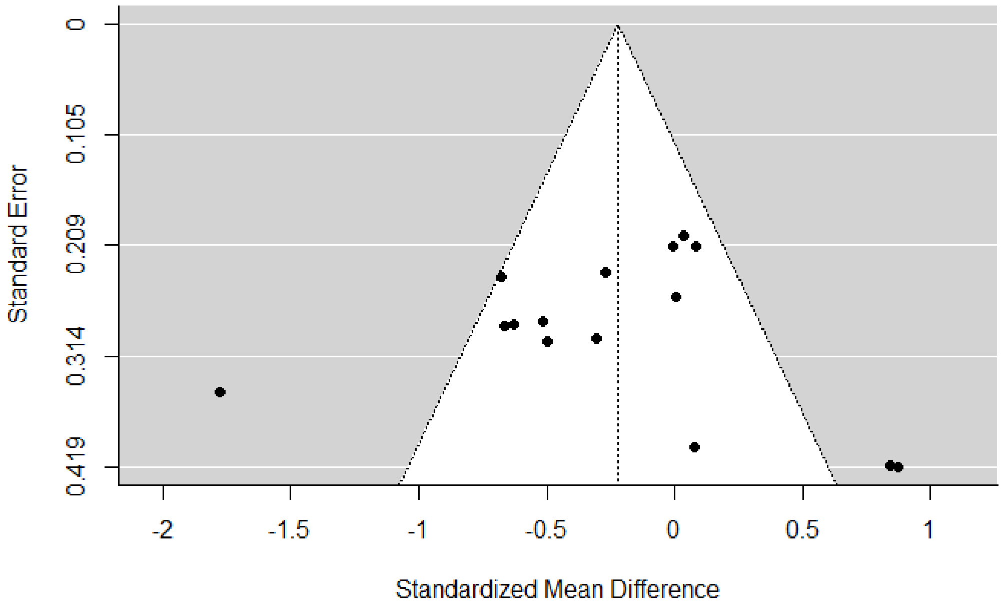


b.

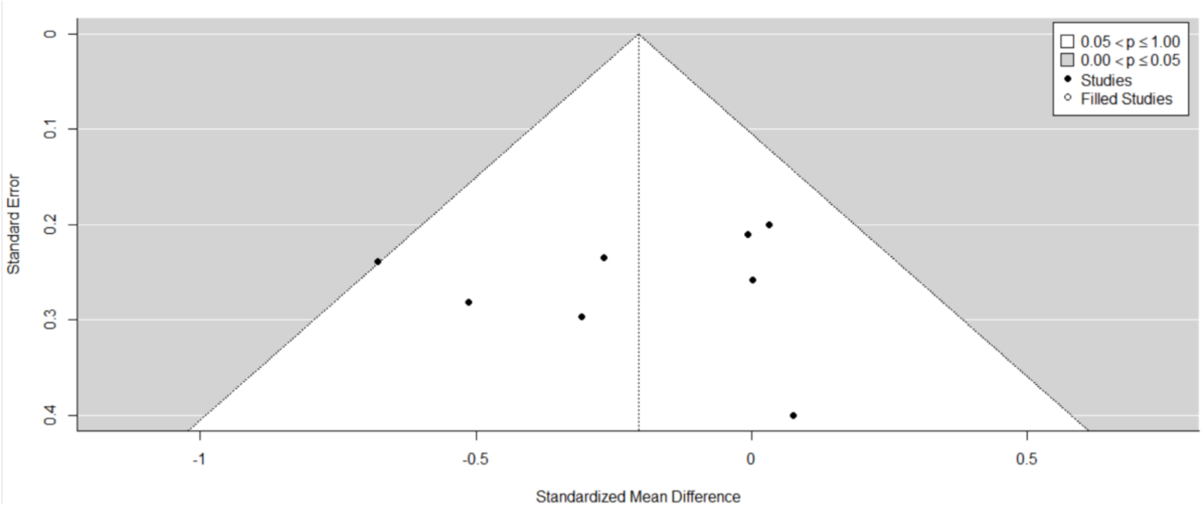


The results did not change when we only included studies on children and adolescents (i.e., excluding [58]) (Hedge's g = -0.1554, SE = 0.2013, 95% CI = -0.5985; 0.2878], t = -0.7716, p = 0.4566, significant heterogeneity: Q = 43.3212; p < 0.0001, publication bias not detected: Kendall's tau = -0.0303, p = 0.9466).

*e. Macular Volume*

*Table S13. Description of studies*

| **Included in the meta-analysis** | |
|---|---|
| **Author, year** | **Hedge's g [variance]** |
| Aslan, 2020 | 0.2602 [0.055] |
| Herguner, 2018 | 0 [0.0444] |

*Figure S23. Forest plot*

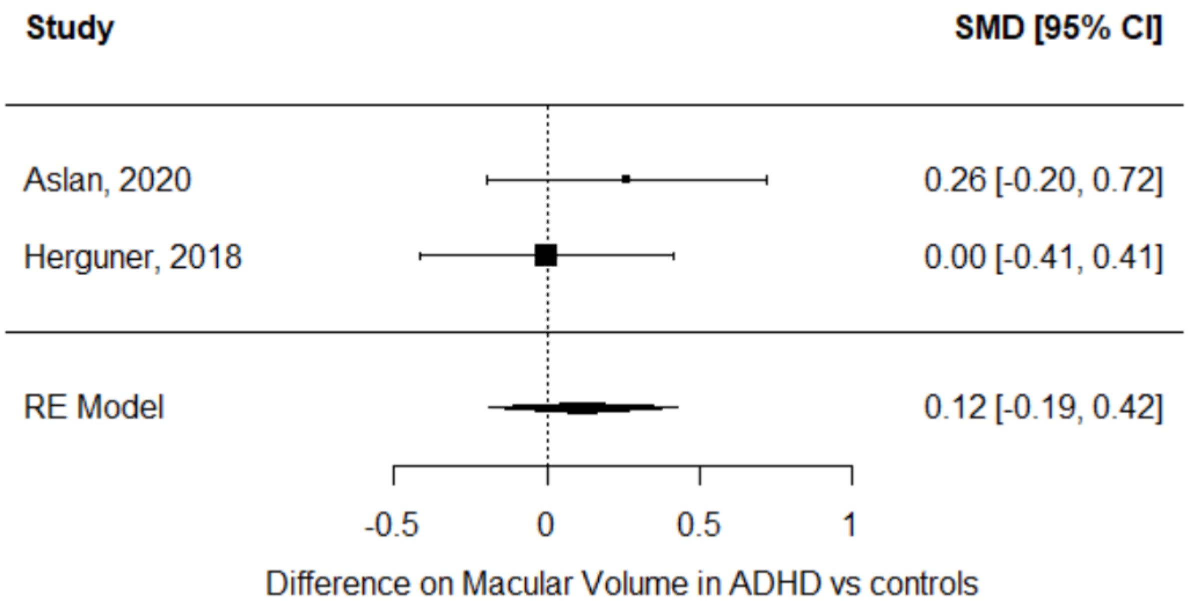


*RE: Random Effects; CI: Confidence Interval; SMD: Standardized Mean Difference*

**Test for Heterogeneity:** Q(df = 1) = 0.6813, p-val = 0.4091

***Figure S24. Funnel plot***

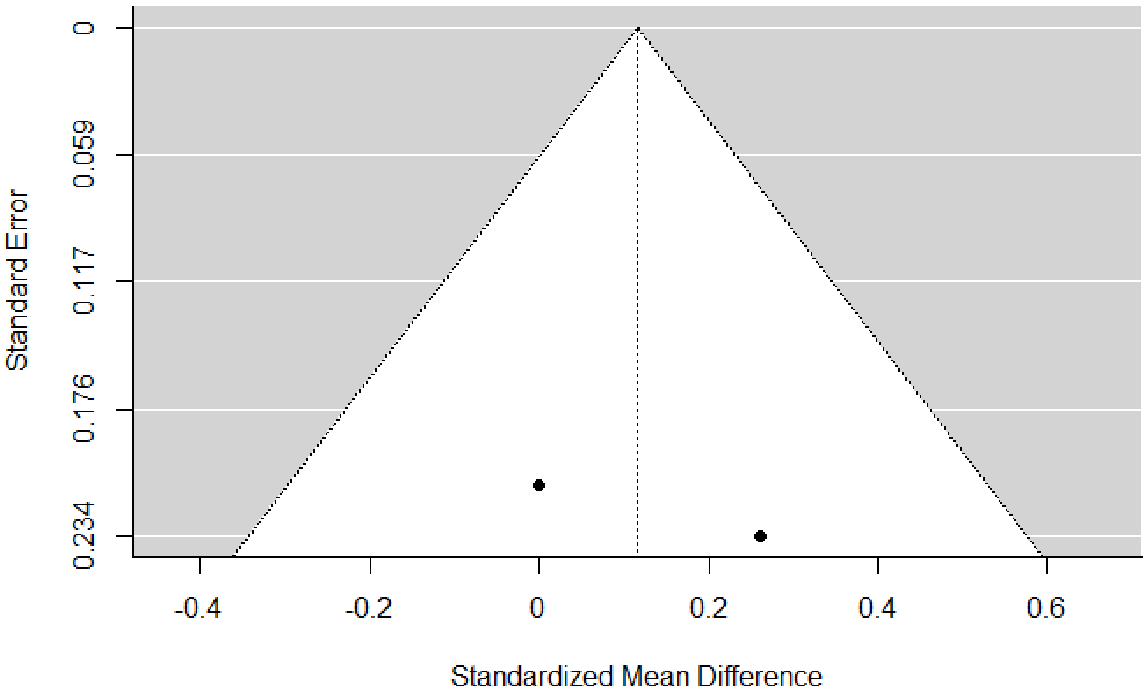

*f. Retinal Nerve Fiber Layer Thickness*

*Table S14. Description of studies*

| **Included in the meta-analysis** | |
|---|---|
| **Author, year** | **Hedge's g [variance]** |
| Aslan, 2020 | 0.2628 [0.055] |
| Ayyildiz, 2019 | -0.2952 [0.0674] |
| Bodur, 2018 | -0.3966 [0.0658] |
| Herguner, 2018 | -0.1141 [0.0445] |
| Işik, 2020 | -0.1396 [0.0401] |
| Sánchez-Guillén, 2020 | -0.2973 [0.0879] |
| Tunel, 2021 | -0.8807 [0.0844] |
| Ulucan Atas, 2020 | 0.0766 [0.0541] |

*Figure S25. Forest plots*

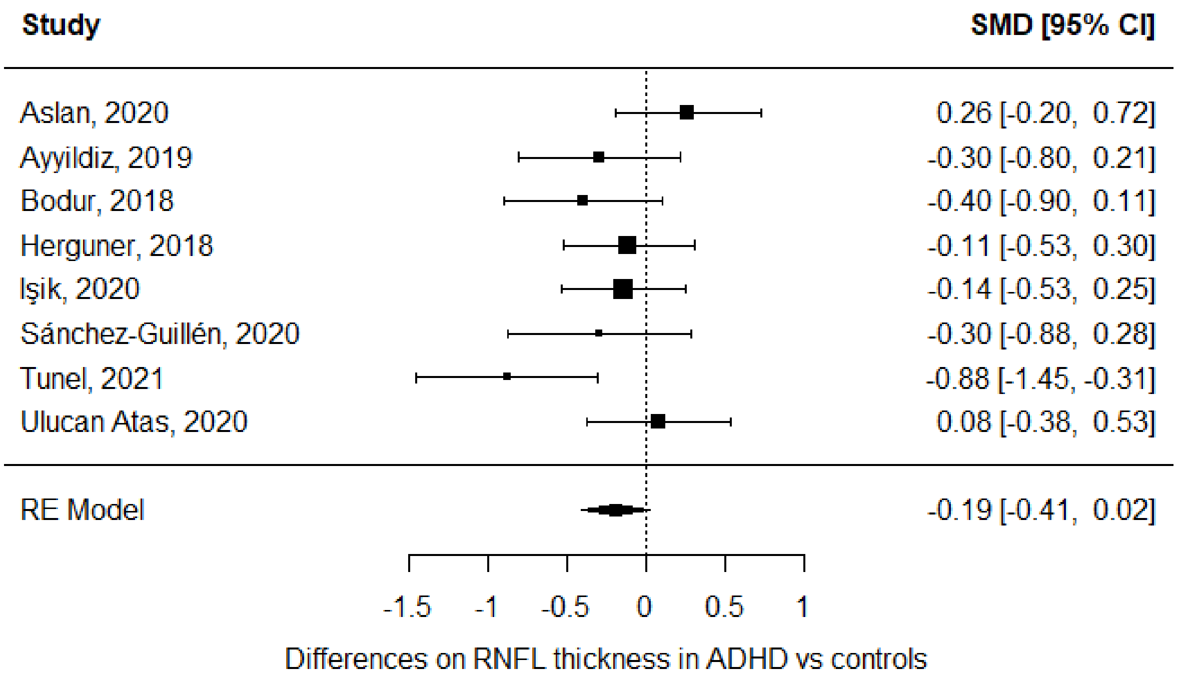


*RE: Random Effects; CI: Confidence Interval; SMD: Standardized Mean Difference; RNFL: Retinal Nerve Fiber Layer Thickness*

**Test for Heterogeneity:** Q(df = 7) = 11.8039, p-val = 0.1072

***Figure S26. Funnel plots (a. no trim and fill analysis; b. trim and fill analysis)***

a.

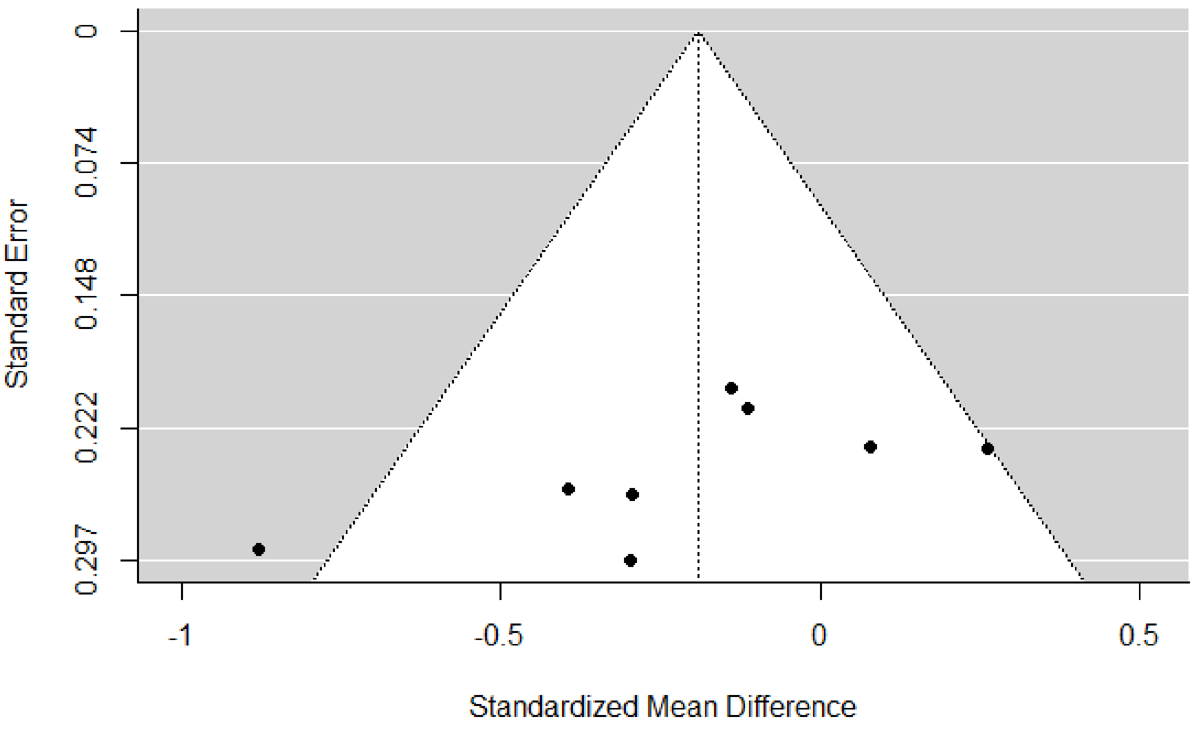


b.

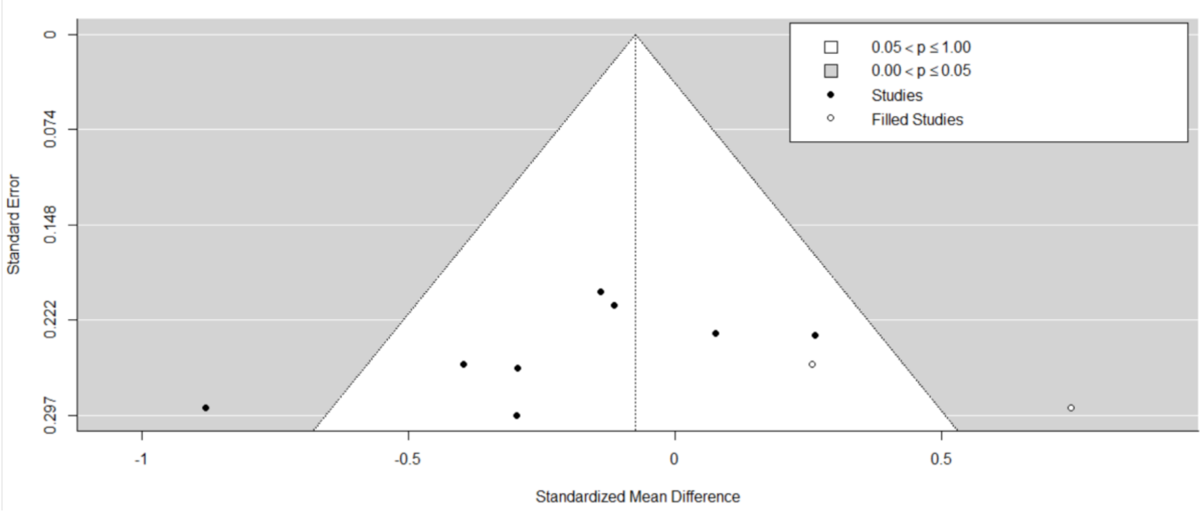


The results did not change when we only included studies on children and adolescents (i.e., excluding [58]) (Hedge's g = -0.1087, SE = 0.0893, 95% CI = -0.2836; 0.0663], t = -1.2175, p = 0.2234, non-significant heterogeneity: Q = 5.3504; p = 0.4997).

## Supplement 9: Results, forest and funnel plots for studies investigating differences on functional measures of vision

### *a. Color discrimination*

*Table S15. Description of studies*

**Included in the meta-analysis**

| **Author, year** | **Hedge's g [variance]** |
|---|---|
| Banaschewski, 2006.1 | 1.0232 [0.1677] |
| Banaschewski, 2006.2 | 0.7227 [0.158] |
| Banaschewski, 2006.3 | 0.9659 [0.1656] |
| Kim, 2014a.1 | -0.5233 [0.0689] |
| Kim, 2014a.2 | -0.7719 [0.0716] |
| Kim, 2014a.3 | 0.3113 [0.0675] |
| Kim, 2014a.4 | 0.1039 [0.0668] |
| Kim, 2014a.5 | -0.294 [0.0674] |
| Kim, 2014a.6 | -0.2219 [0.0671] |
| Kim, 2014c.1 | 0.5748 [0.0694] |
| Kim, 2014c.2 | 0.5051 [0.0688] |
| Kim, 2014c.3 | 0.4354 [0.0682] |
| Kim, 2014c.4 | 0.5875 [0.0695] |
| Kim, 2014c.5 | 0.3478 [0.0677] |
| Kim, 2015.1 | 0.1583 [0.1296] |
| Kim, 2015.2 | -0.2668 [0.1303] |
| Kim, 2015.3 | -0.0196 [0.1292] |
| Roessner, 2008.1 | 1.1968 [0.1684] |
| Roessner, 2008.2 | 0.9534 [0.1591] |
| Roessner, 2008.3 | 1.1891 [0.1681] |
| Uebel-von Sandersleben, 2017.1 | 1.0811 [0.1582] |
| Uebel-von Sandersleben, 2017.2 | 0.8109 [0.1494] |
| Uebel-von Sandersleben, 2017.3 | 1.0506 [0.1571] |

*Figure S27. Forest plot*

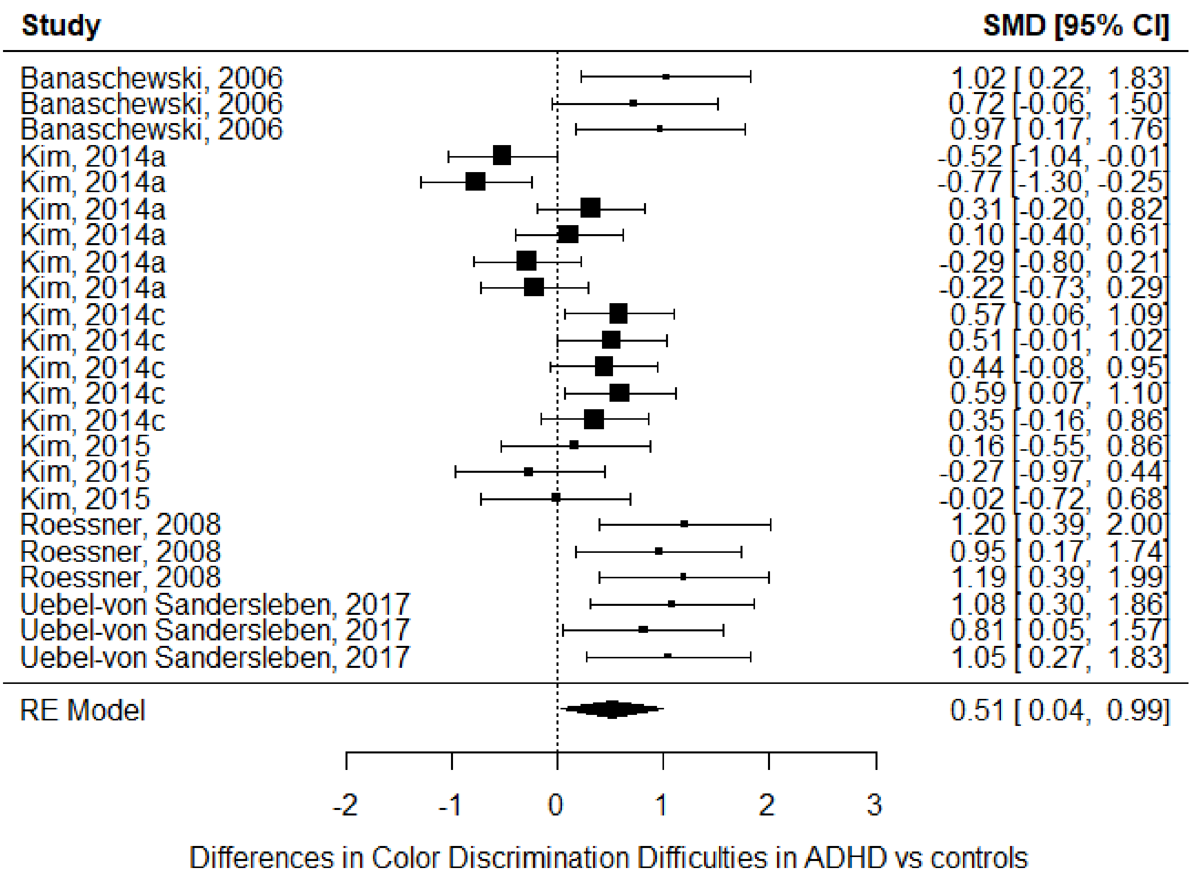


*RE: Random Effects; CI: Confidence Interval; SMD: Standardized Mean Difference*

**Test for Heterogeneity:** Q(df = 22) = 70.6120, p-val < .0001

*Figure S28. Funnel plots (a. no trim and fill analysis; b. trim and fill analysis excluding largest effect sizes for each study reporting more than one effect size, c. trim and fill analysis excluding smallest effect sizes for each study reporting more than one effect size)*

**Rank Correlation Test for Funnel Plot Asymmetry:** Kendall's tau = 0.5810, p < .0001

a.

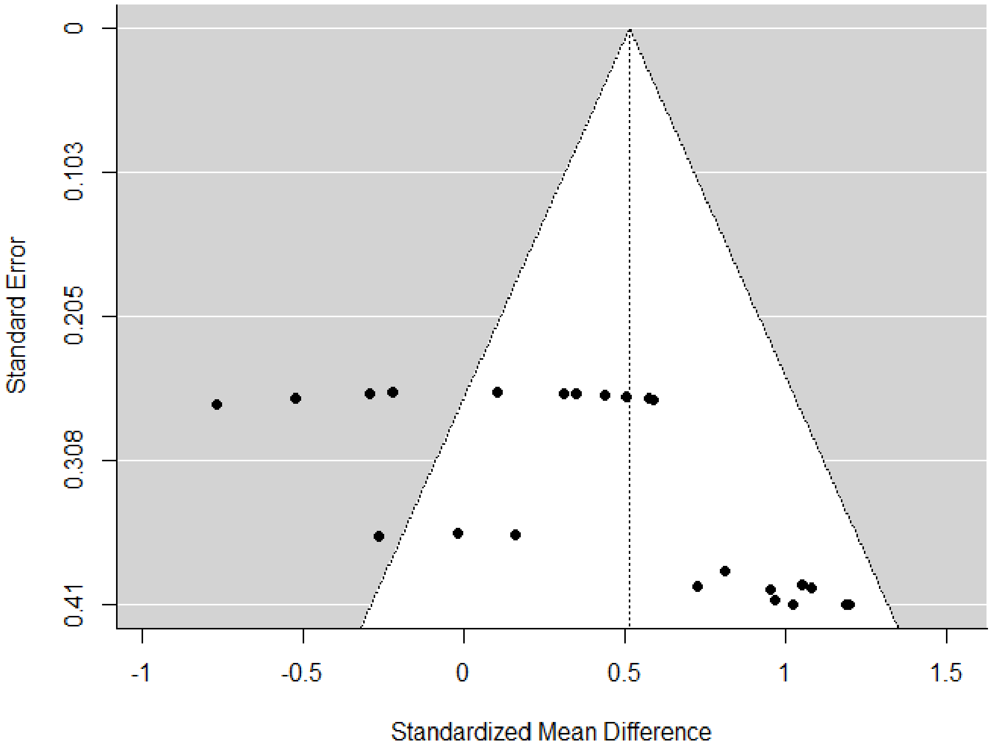


b.

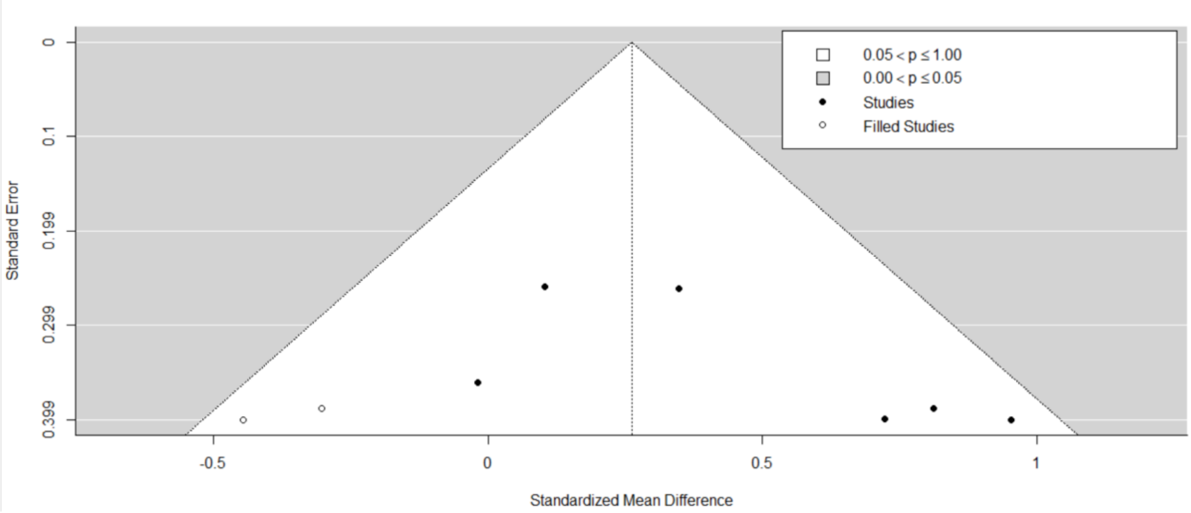


c.

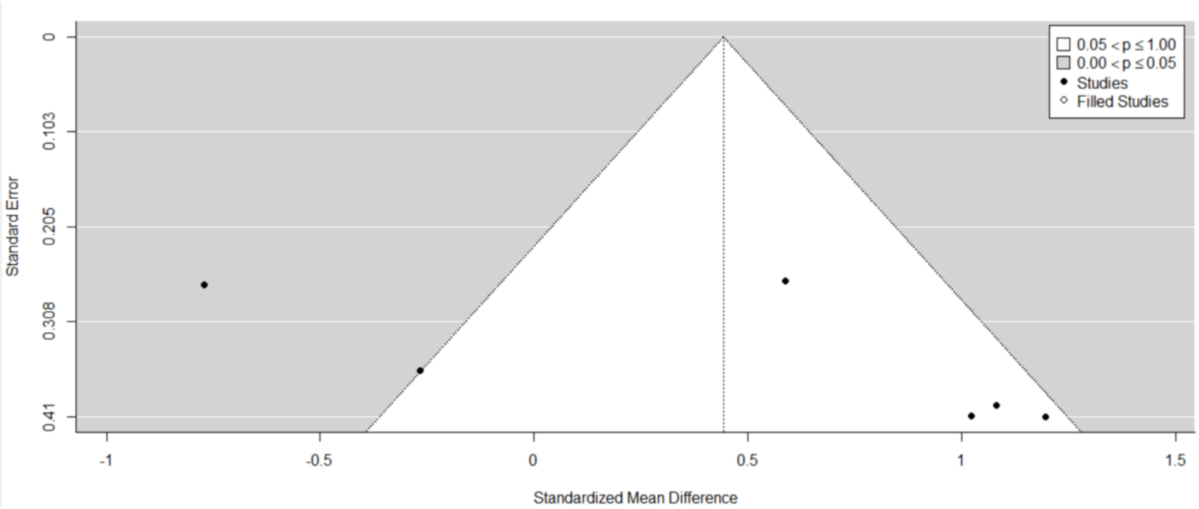


When investigating color discrimination in children and adolescents (i.e., excluding [32,35], which included adults), the meta-analytic model remained significant (Hedge's g = 0.7292, SE = 0.2677, 95% CI = 0.0198; 1.3185], t = 2.7235, p = 0.0198) and, although publication bias was detected (Kendall's tau = 0.6970, p = 0.0010), heterogeneity was marginally not significant (Q = 19.5680; p = 0.0516). We also carried out the meta-analysis on contrast sensitivity only on studies on children and adolescents (i.e., excluding [32], which was on adults), and the model remained significant (Hedge's g = -3.4250, SE = 0.9621, 95% CI = [-5.5686; -1.2814], t = -3.5601, p = 0.0052, with significant heterogeneity (Q = 293.1152; p < 0.0001) and detection of publication bias (Kendall's tau = -0.6364, p = 0.0057).

***b. Contrast sensitivity***

***Table S16. Description of studies***

| **Included in the meta-analysis** | |
|---|---|
| **Author, year** | **Hedge's g [variance]** |
| Dönmez, 2020.1 | -10.4746 [0.981] |
| Dönmez, 2020.2 | -1.6348 [0.0889] |
| Dönmez, 2020.3 | -1.4168 [0.0834] |
| Dönmez, 2020.4 | -1.4023 [0.0831] |
| Dönmez, 2020.5 | -1.2733 [0.0802] |
| Kim, 2014a.1 | 0.0353 [0.0667] |
| Kim, 2014a.2 | -0.7062 [0.0708] |
| Kim, 2015 | 0.1022 [0.1293] |
| Ulucan Atas, 2020.1 | -5.5049 [0.2588] |
| Ulucan Atas, 2020.2 | -7.3306 [0.4171] |
| Ulucan Atas, 2020.3 | -1.5839 [0.071] |
| Ulucan Atas, 2020.4 | -5.7769 [0.2795] |
| Ulucan Atas, 2020.5 | -2.1503 [0.0853] |
| **Included in the narrative review** | |
| **Author, year** | **Descriptive summary of findings** |
| Bartgis, 2009 | Reduced contrast sensitivity in ADHD compared to no-ADHD |
| Brown, 2020 | No differences between ADHD and no-ADHD on flicker fusion thresholds |
| Kim, 2014b | Reduced contrast sensitivity in ADHD compared to no-ADHD |

*Figure S29. Forest plot*

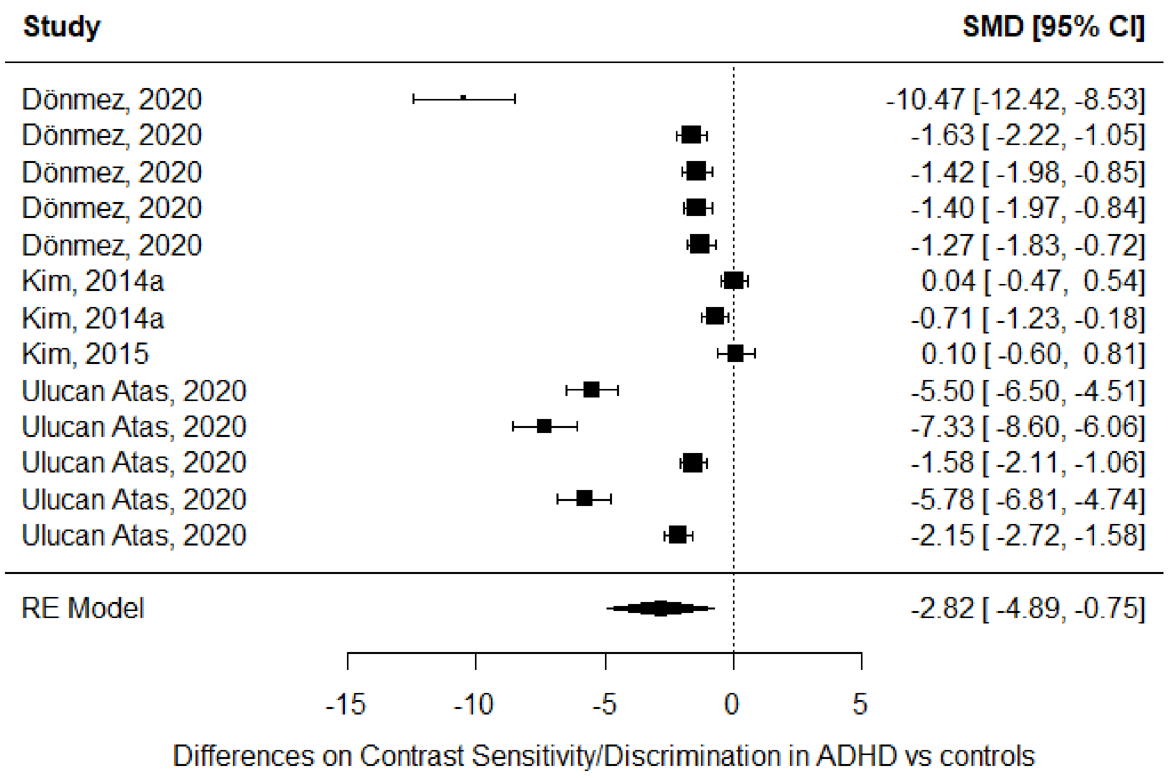


*RE: Random Effects; CI: Confidence Interval; SMD: Standardized Mean Difference*

**Test for Heterogeneity:** Q(df = 12) = 359.8974, p-val < .0001

*Figure S30. Funnel plots (a. no trim and fill analysis; b. trim and fill analysis excluding largest effect sizes for each study reporting more than one effect size, c. trim and fill analysis excluding smallest effect sizes for each study reporting more than one effect size)*

**Rank Correlation Test for Funnel Plot Asymmetry:** Kendall's tau = -0.7179, p = 0.0003

a.

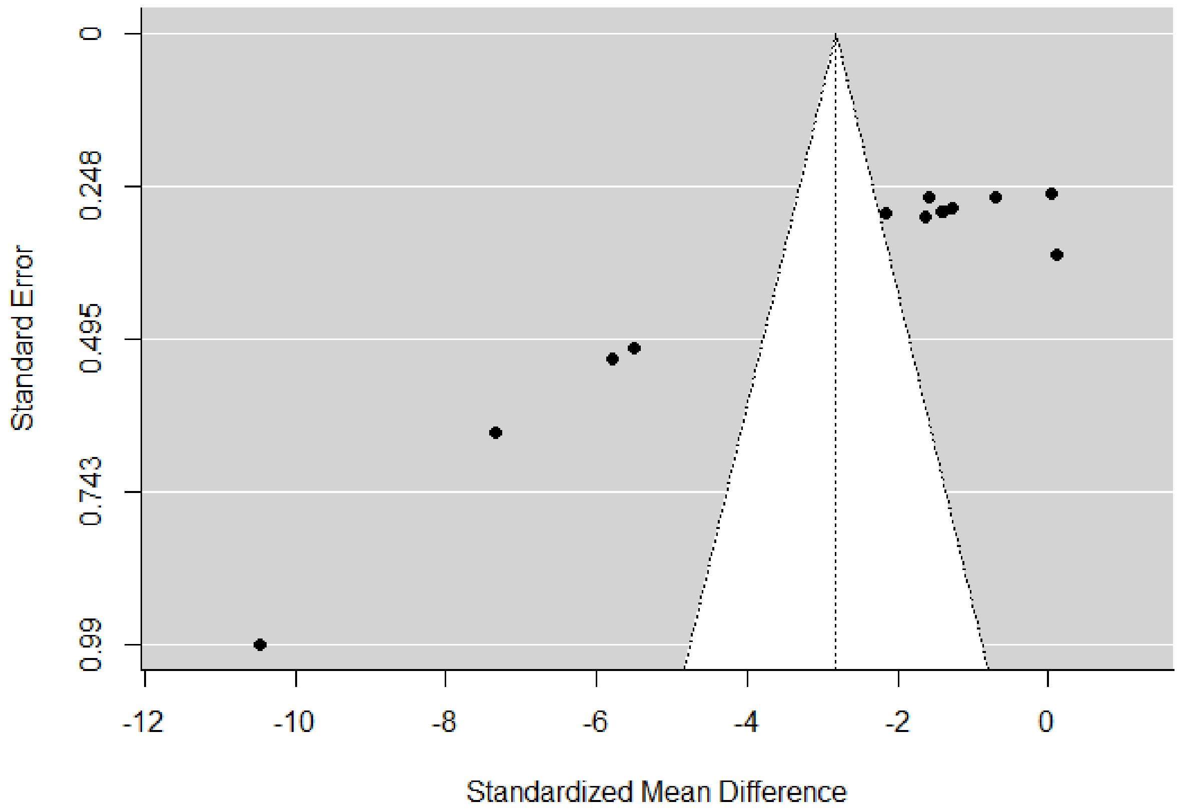


b.

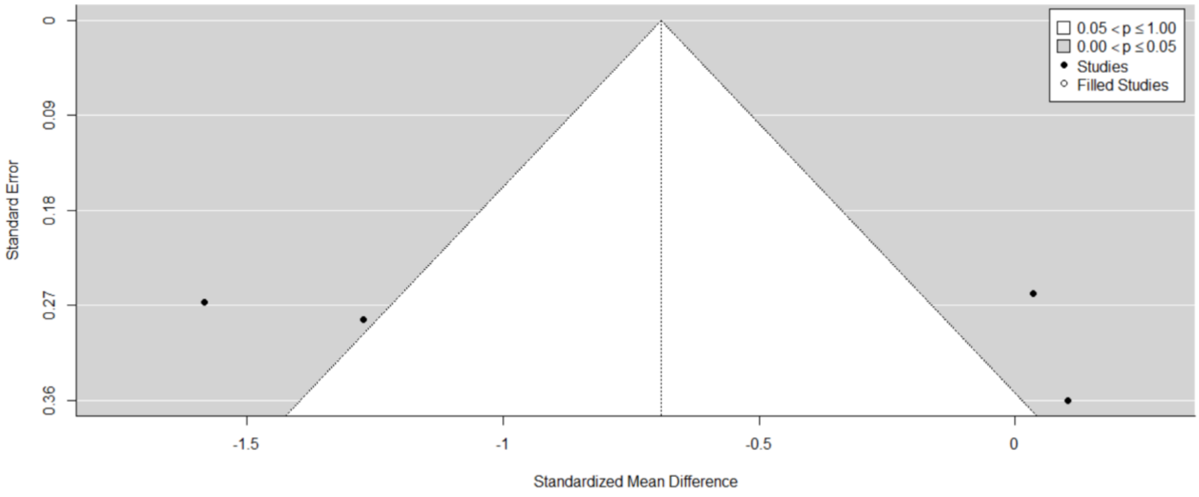

c.

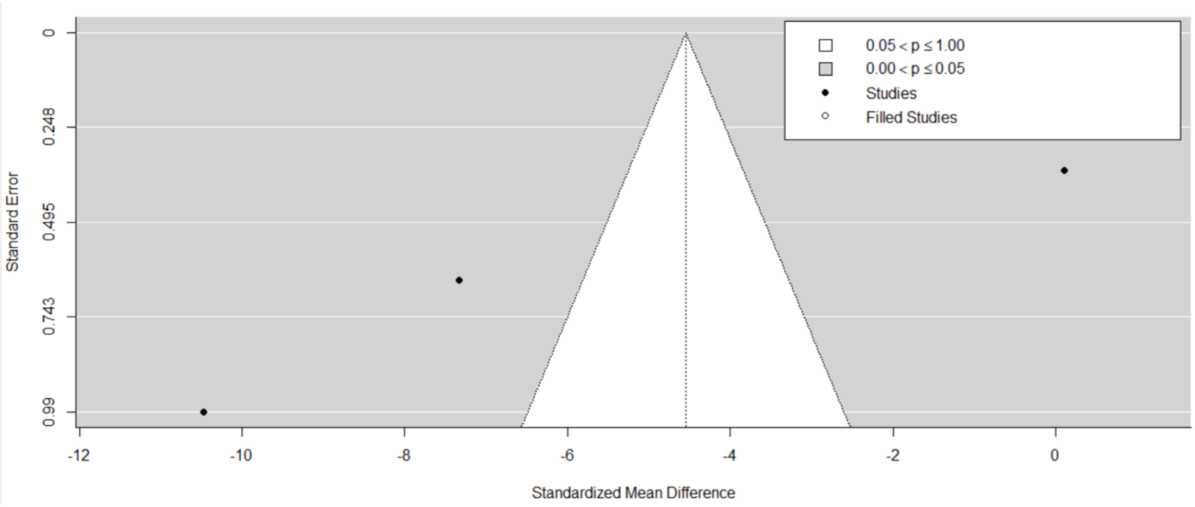
0.05 < p ≤ 1.00
0.00 < p ≤ 0.05
Studies
Filled Studies
Standard Error
0
0.248
0.495
0.743
0.99
-12
-10
-8
-6
-4
-2
0
Standardized Mean Difference

**Supplement 10: Results, forest and funnel plots for studies investigating differences on measures of visual acuity**

***a. Accommodation: lag***

***Table S17. Description of studies***

| **Included in the meta-analysis** | |
|---|---|
| **Author, year** | **Hedge's g [variance]** |
| Redondo, 2018.1 | 0.5956 [0.116] |
| Redondo, 2018.2 | 0.6042 [0.1162] |
| Redondo, 2020a | 0.5451 [0.0785] |
| Redondo, 2020b.1 | 0.7985 [0.0982] |
| Redondo, 2020b.2 | 0.7269 [0.0969] |
| Redondo, 2020b.3 | 0.5198 [0.094] |
| **Included in the narrative review** | |
| **Author, year** | **Descriptive summary of findings** |
| Fabian, 2013 | No differences on the amplitude of the accommodative response between people with and without ADHD |

***Figure S31. Forest plot***

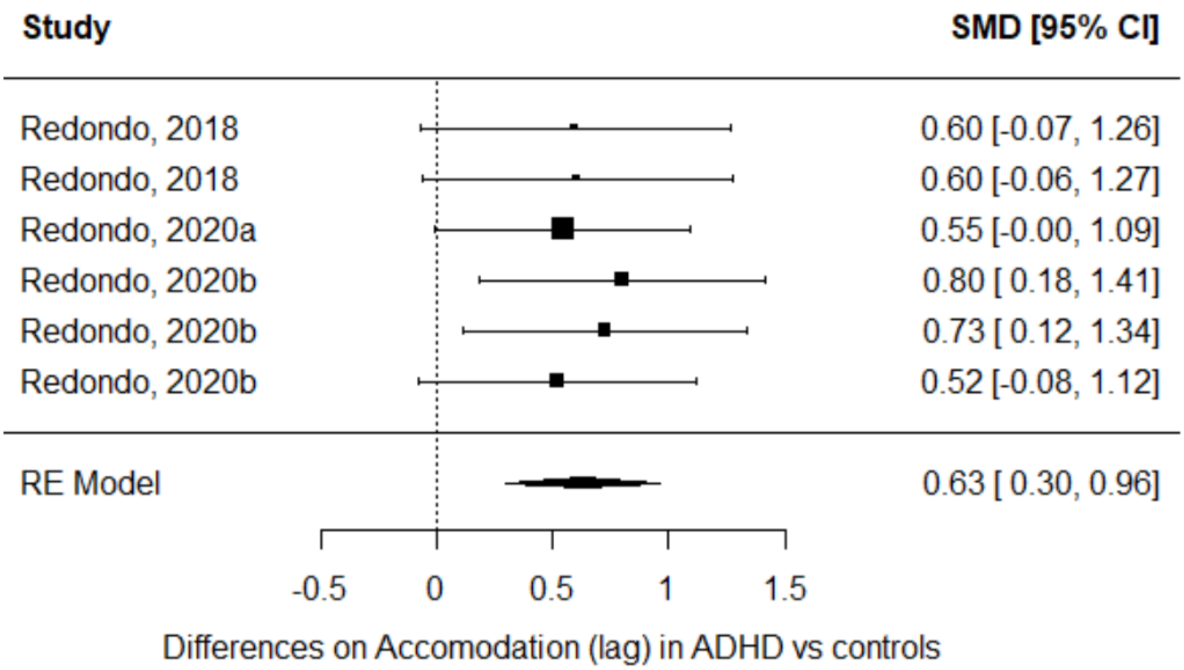


*RE: Random Effects; CI: Confidence Interval; SMD: Standardized Mean Difference*

**Test for Heterogeneity:** Q(df = 5) = 0.6230, p-val = 0.9869

***Figure S32. Funnel plots (a. no trim and fill analysis; b. trim and fill analysis excluding largest effect sizes for Redondo, 2018 and Redondo, 2020b, c. trim and fill analysis excluding smallest effect sizes for Redondo, 2018 and Redondo, 2020b)***

**Rank Correlation Test for Funnel Plot Asymmetry:** Kendall's tau = 0.3333, p = 0.4694

a.

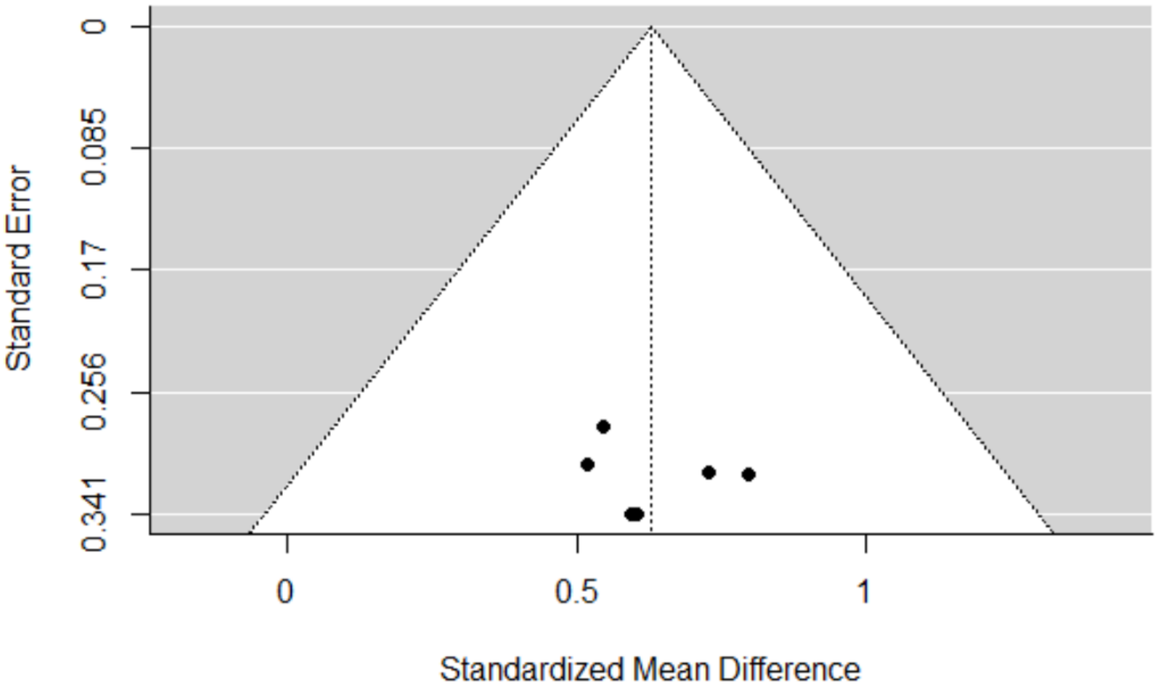


b.

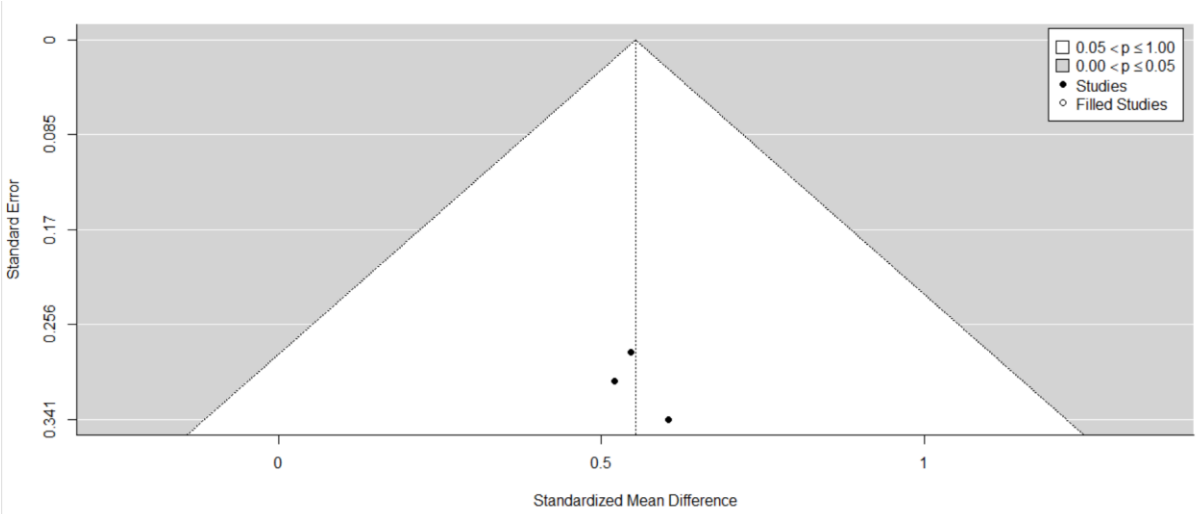

c.

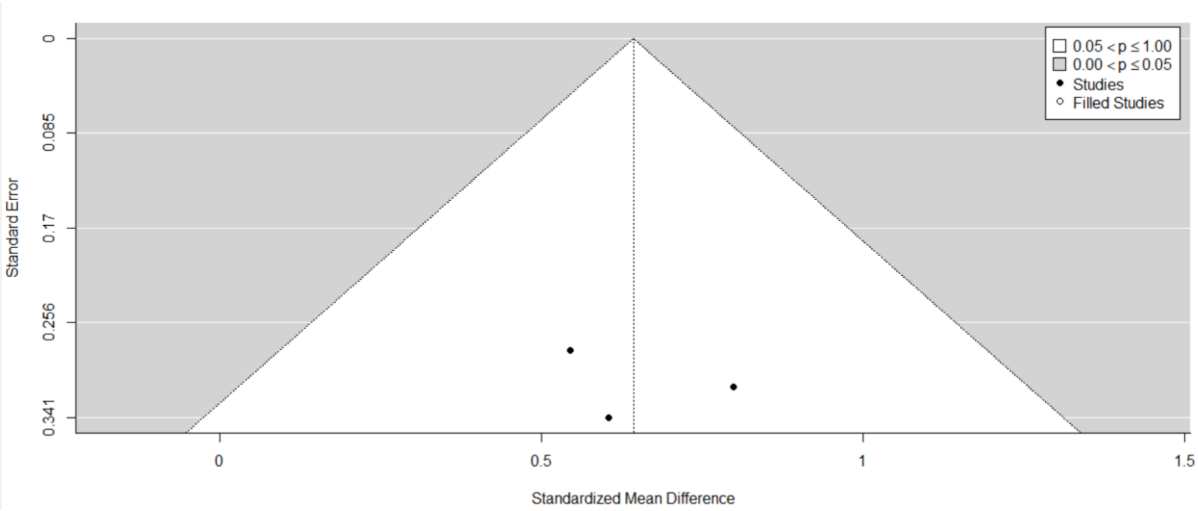
0.05 < p ≤ 1.00
0.00 < p ≤ 0.05
Studies
Filled Studies
Standard Error
0
0.085
0.17
0.256
0.341
0
0.5
1
1.5
Standardized Mean Difference

***b. Accommodation: variability***

***Table S18. Description of studies***

| **Included in the meta-analysis** | |
|---|---|
| **Author, year** | **Hedge's g [variance]** |
| Redondo, 2018.1 | 0.5956 [0.116] |
| Redondo, 2018.2 | 0.6042 [0.1162] |
| Redondo, 2020a.1 | 0.5451 [0.0785] |
| Redondo, 2020a.2 | 0.4312 [0.0775] |
| Redondo, 2020b.1 | 0.7985 [0.0982] |
| Redondo, 2020b.2 | 0.7269 [0.0969] |
| Redondo, 2020b.3 | 0.5198 [0.094] |
| Redondo, 2020b.4 | -0.2025 [0.0914] |
| Redondo, 2020b.5 | 0.0849 [0.0910] |
| Redondo, 2020b.6 | 0.0312 [0.0909] |

***Figure S33. Forest plot***

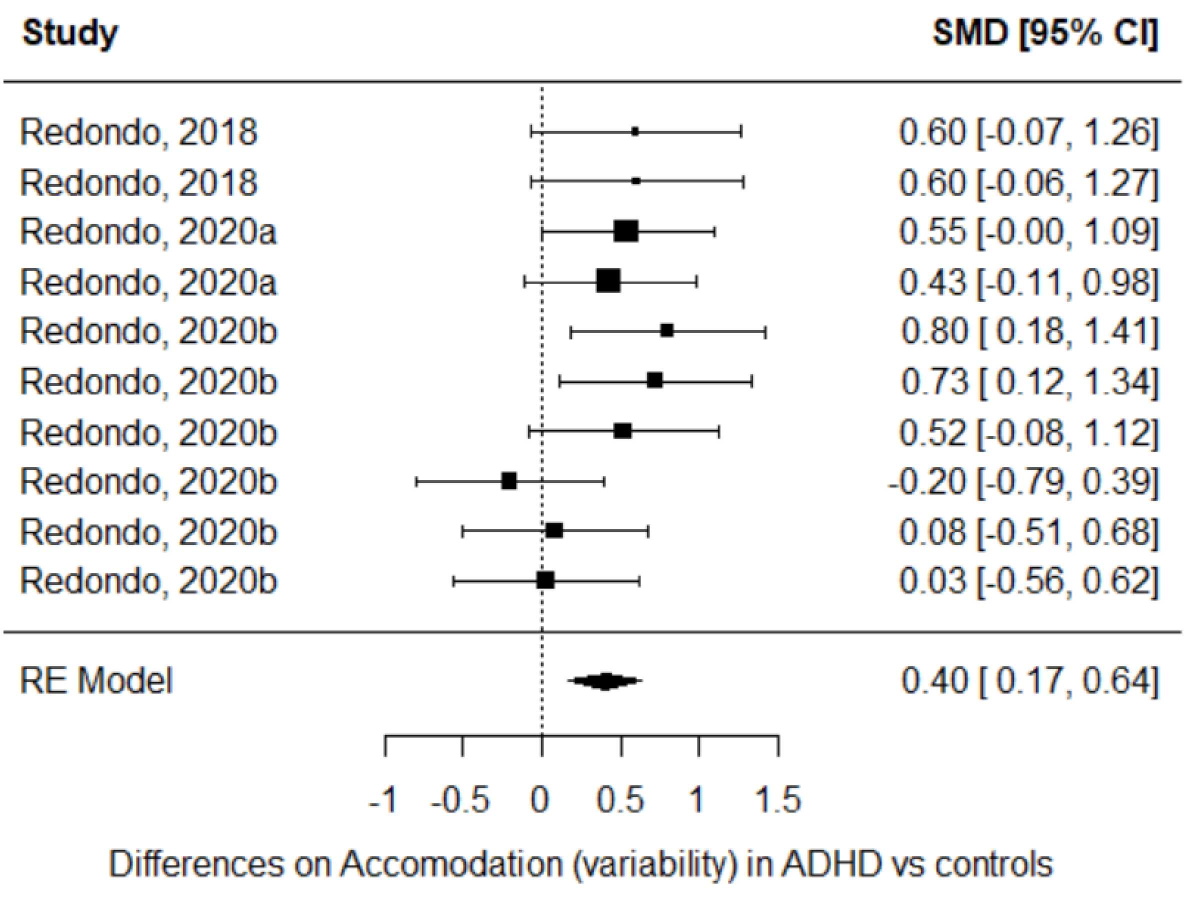


*RE: Random Effects; CI: Confidence Interval; SMD: Standardized Mean Difference*

**Test for Heterogeneity:** Q(df = 9) = 10.4021, p-val = 0.3189

***Figure S34. Funnel plots (a. no trim and fill analysis; b. trim and fill analysis excluding largest effect sizes for Redondo 2018, Redondo 2020a and Redondo 2020b, c. trim and fill analysis excluding smallest effect sizes for Redondo 2018, Redondo 2020a and Redondo 2020b)***

**Rank Correlation Test for Funnel Plot Asymmetry:** Kendall's tau = 0.4222, p = 0.1083

a.

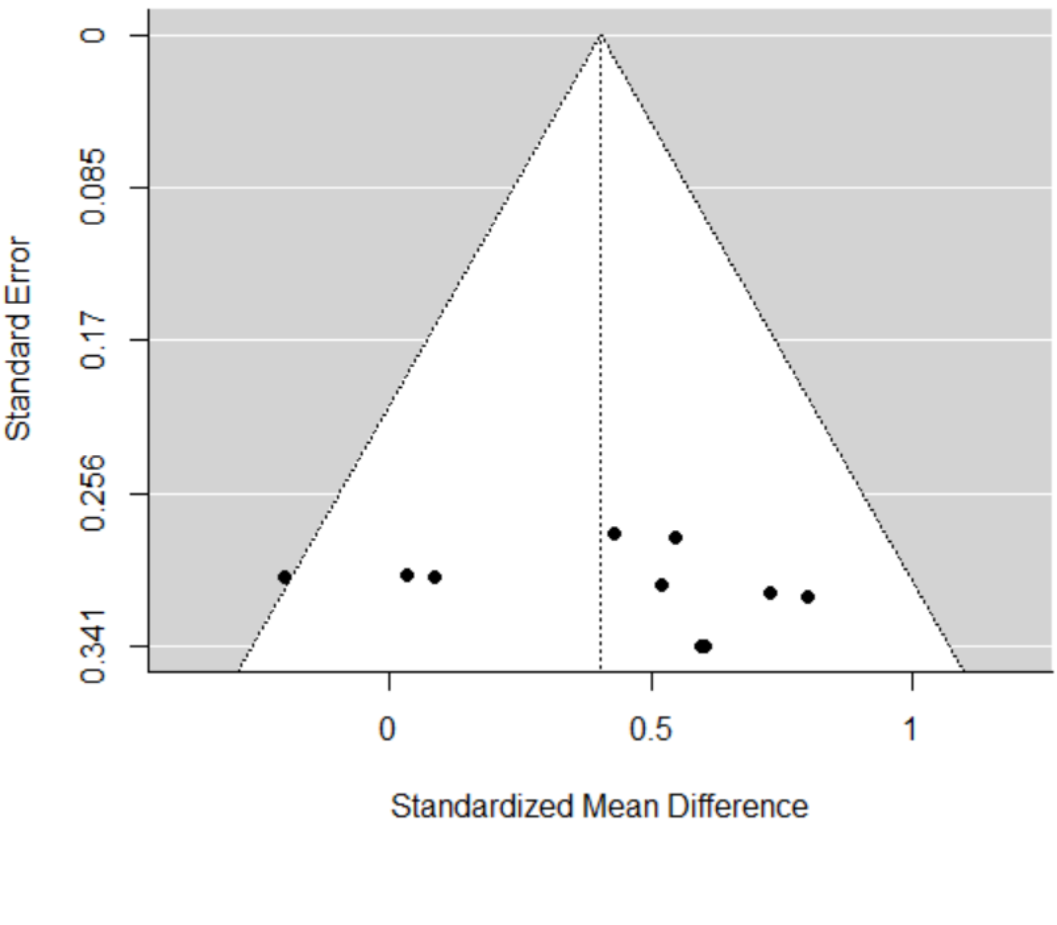


b.

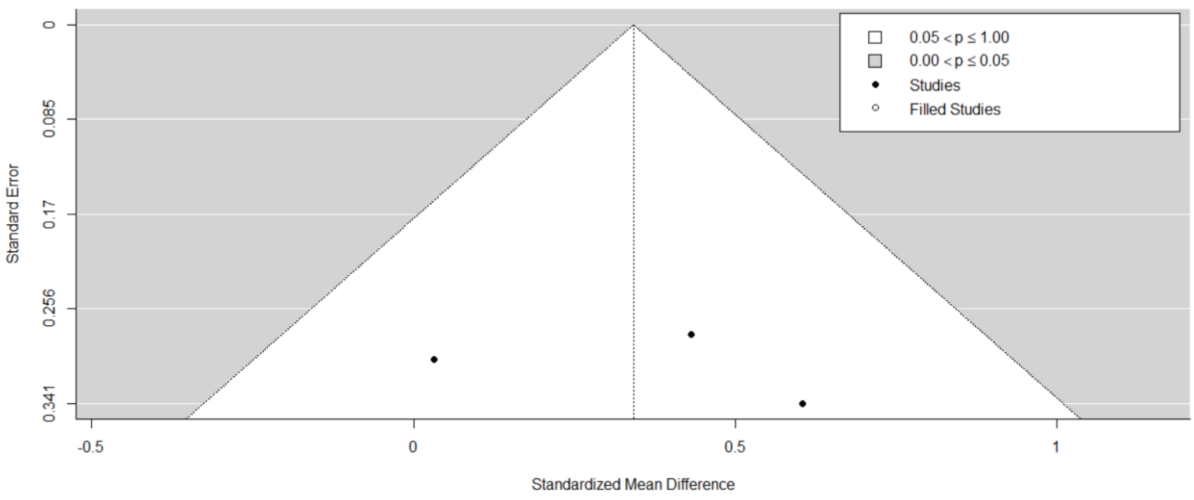
0.05 < p ≤ 1.00
0.00 < p ≤ 0.05
Studies
Filled Studies
Standard Error
0
0.085
0.17
0.256
0.341
-0.5
0
0.5
1
Standardized Mean Difference

c.

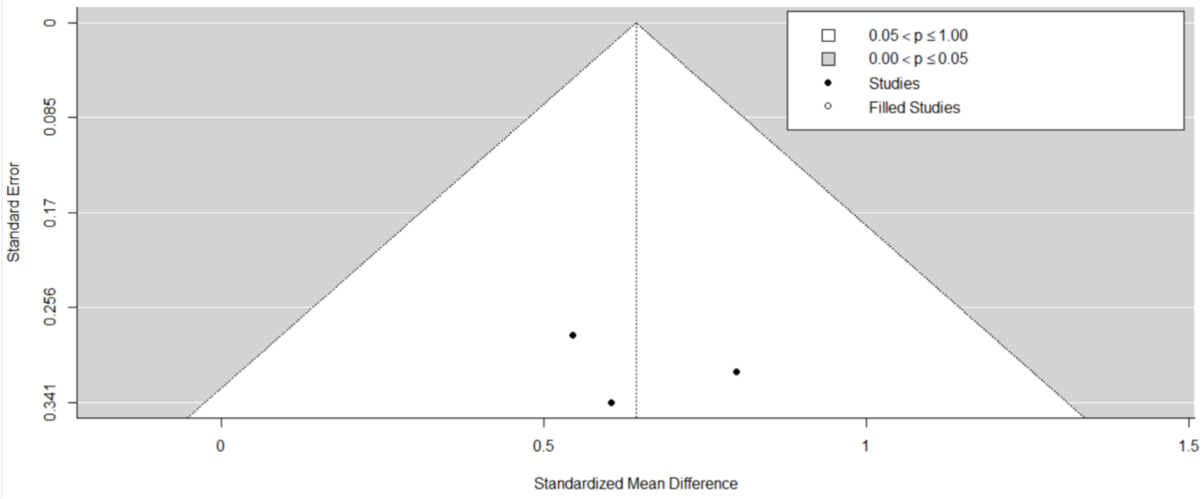
0.05 < p ≤ 1.00
0.00 < p ≤ 0.05
Studies
Filled Studies
Standard Error
0
0.085
0.17
0.256
0.341
0
0.5
1
1.5
Standardized Mean Difference

*c. Refractive Error (Spherical Equivalents)*

***Table S19. Description of studies***

| **Included in the meta-analysis** | |
|---|---|
| **Author, year** | **Hedge's g [variance]** |
| Bae, 2019.1 | -0.2034 [0.1611] |
| Bae, 2019.2 | -0.3543 [0.1628] |
| Gronlund, 2007.1 | 0.3624 [0.0445] |
| Gronlund, 2007.2 | 0.3372 [0.0444] |
| Karaca, 2020 | 0.3304 [0.0651] |
| Redondo, 2018 | 0.4333 [0.1137] |
| Redondo, 2020a | -0.2849 [0.0765] |
| Sánchez-Guillén, 2020 | -0.232 [0.0875] |
| **Included in the narrative review** | |
| **Author, year** | **Descriptive summary of findings** |
| Ababneh, 2020 | No differences in refraction between children with and without ADHD |
| Fabian, 2013 | No differences in refraction between children with and without ADHD |
| Kim, 2015 | No differences in refraction between children with and without ADHD |
| Martin, 2008 | No differences in refraction between children with and without ADHD |

***Figure S35. Forest plot***

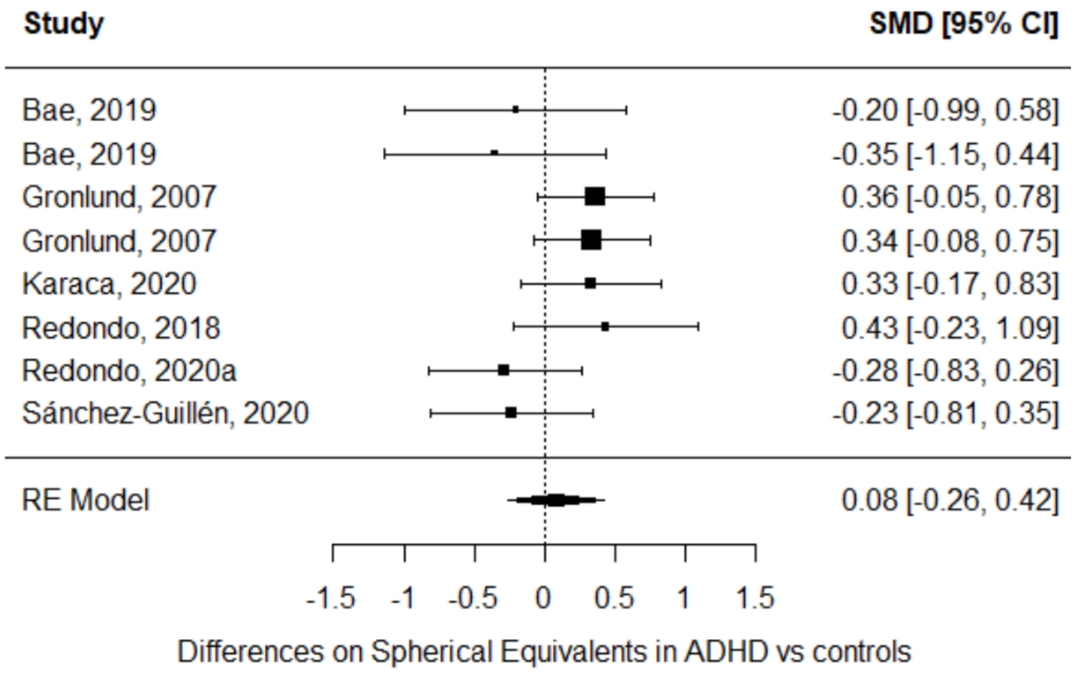


*RE: Random Effects; CI: Confidence Interval; SMD: Standardized Mean Difference*

**Test for Heterogeneity:** Q(df = 7) = 9.4744, p-val = 0.2204

***Figure S36. Funnel plots (a. no trim and fill analysis; b. trim and fill analysis excluding Bae 2019.2 and Gronlund 2007.2, c. trim and fill analysis excluding Bae 2019.1 and Gronlund 2007.1, d. trim and fill analysis excluding Bae 2019.2 and Gronlund 2007.1, e. trim and fill analysis excluding Bae 2019.1 and Gronlund 2007.2)***

**Rank Correlation Test for Funnel Plot Asymmetry:** Kendall's tau = -0.3571, p = 0.2751

a.

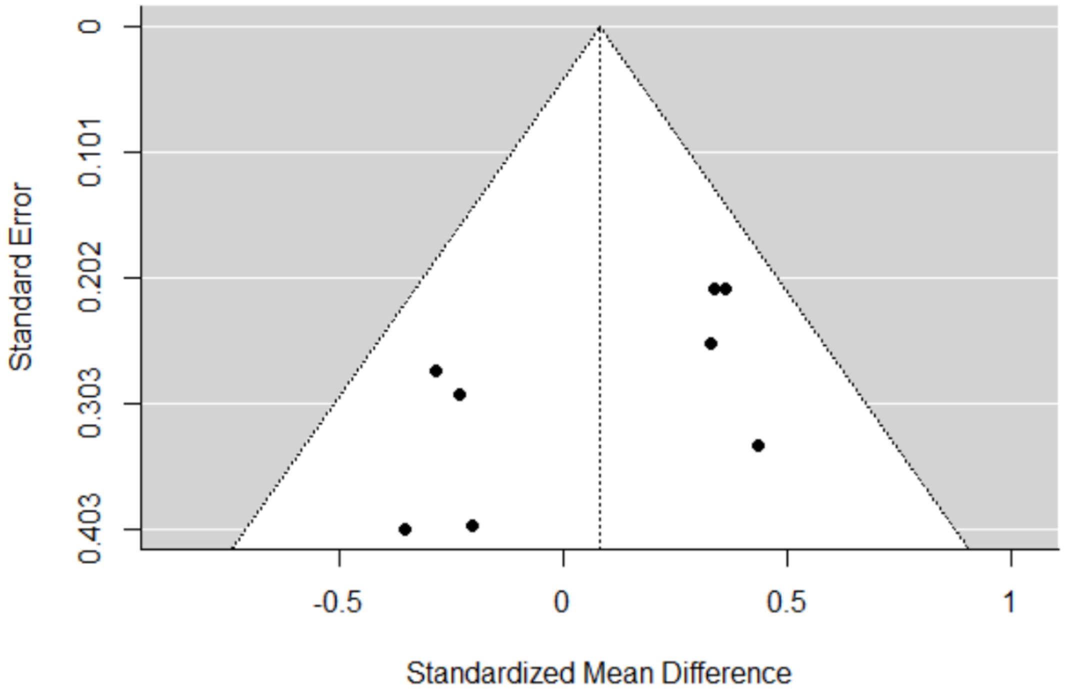


b.

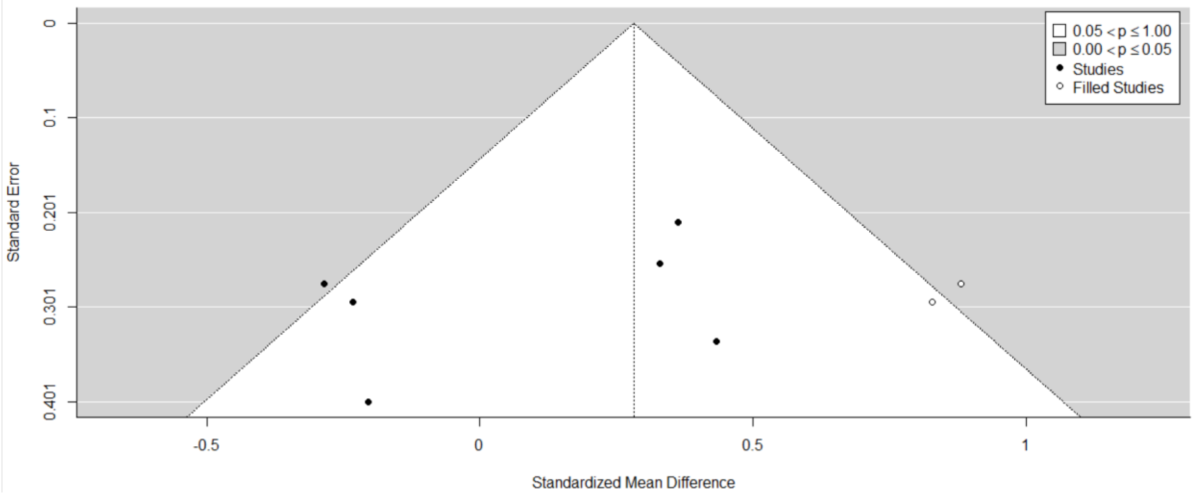

c.

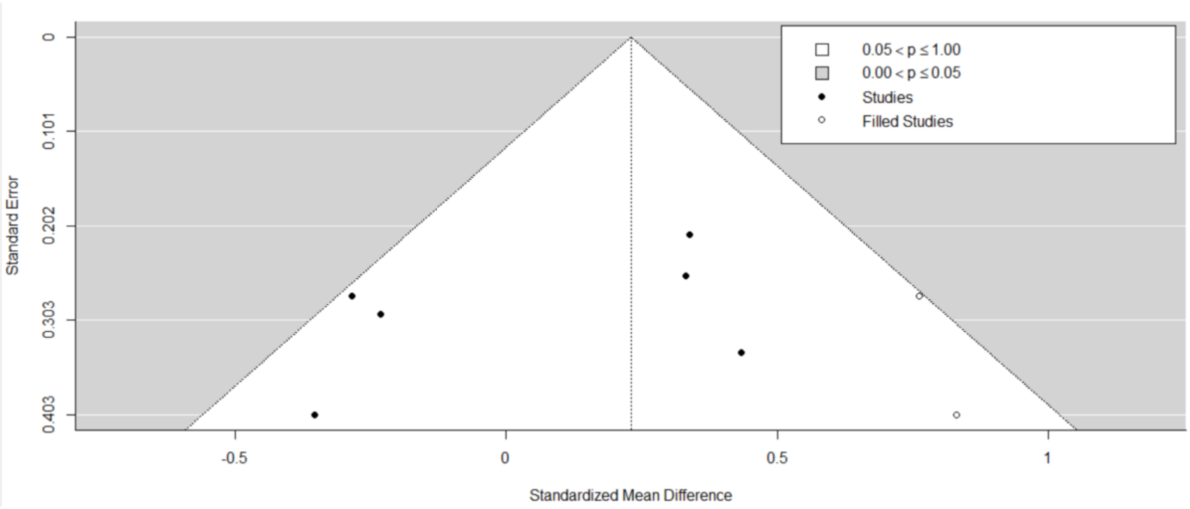
0.05 < p ≤ 1.00
0.00 < p ≤ 0.05
Studies
Filled Studies
Standard Error
0
0.101
0.202
0.303
0.403
-0.5
0
0.5
1
Standardized Mean Difference

d.

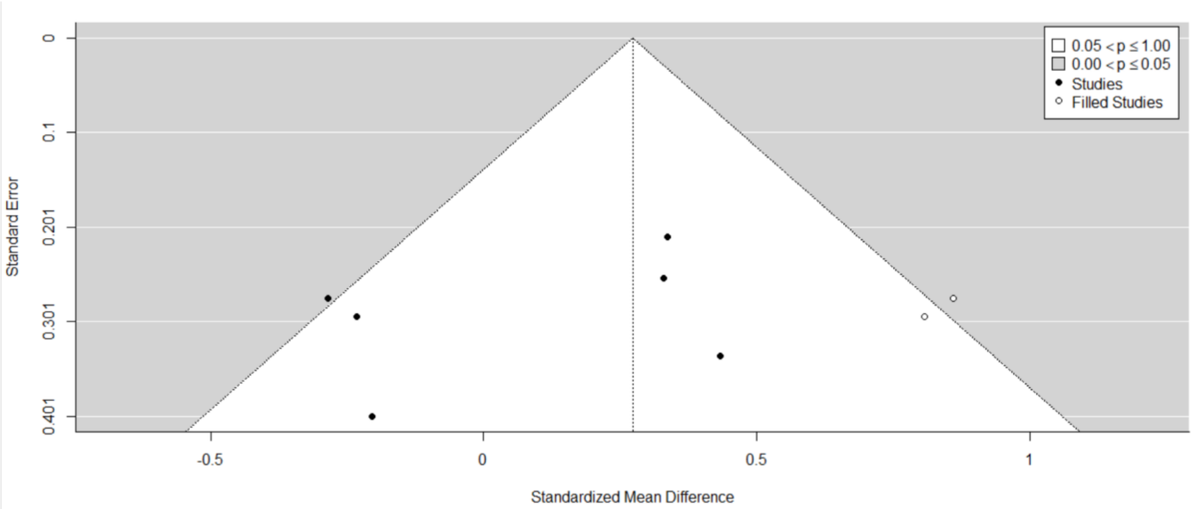
0.05 < p ≤ 1.00
0.00 < p ≤ 0.05
Studies
Filled Studies
Standard Error
0
0.1
0.201
0.301
0.401
-0.5
0
0.5
1
Standardized Mean Difference

e.

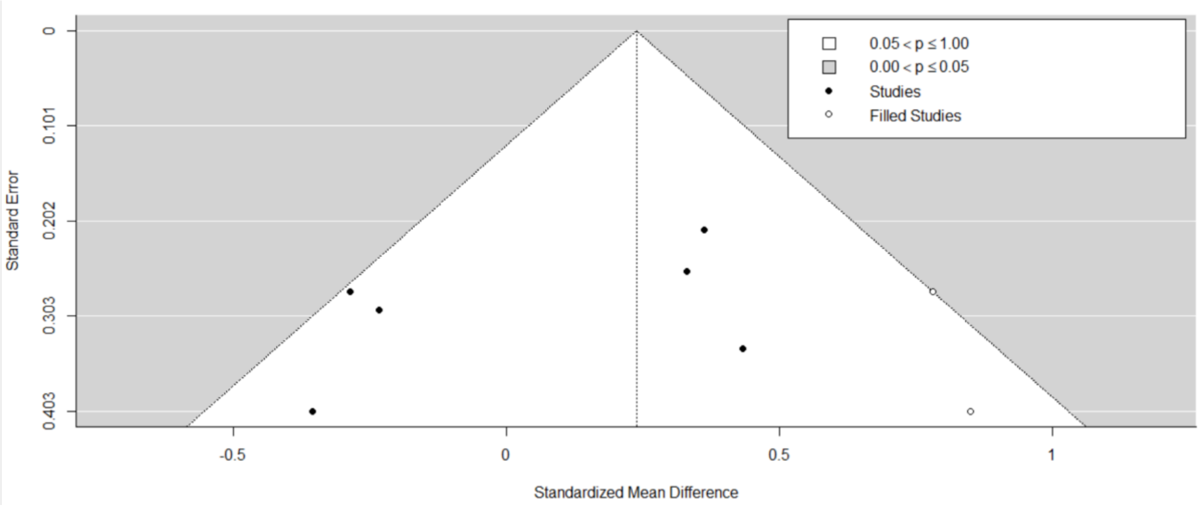
0.05 < p ≤ 1.00
0.00 < p ≤ 0.05
Studies
Filled Studies
Standard Error
0
0.101
0.202
0.303
0.403
-0.5
0
0.5
1
Standardized Mean Difference

*d. Visual Acuity*

*Table S20. Description of studies*

| **Included in the meta-analysis** | |
|---|---|
| **Author, year** | **Hedge's g [variance]** |
| DeCarlo, 2014.1 | -0.2907 [0.0233] |
| DeCarlo, 2014.2 | -0.4161 [0.0235] |
| Fabian, 2013 | 0.1242 [0.0331] |
| Kim, 2015 | -0.2597 [0.1303] |
| Redondo, 2018 | 1.2393 [0.1324] |
| **Included in the narrative review** | |
| **Author, year** | **Descriptive summary of findings** |
| Ababneh, 2020 | No differences in visual acuity between ADHD and no-ADHD |
| Gronlund, 2007 | Reduced visual acuity in ADHD vs no-ADHD |
| Martin, 2008 | Reduced visual acuity in ADHD vs no-ADHD |
| Sánchez-Guillén, 2020 | No differences in visual acuity between ADHD and no-ADHD |

*Figure S37. Forest plot*

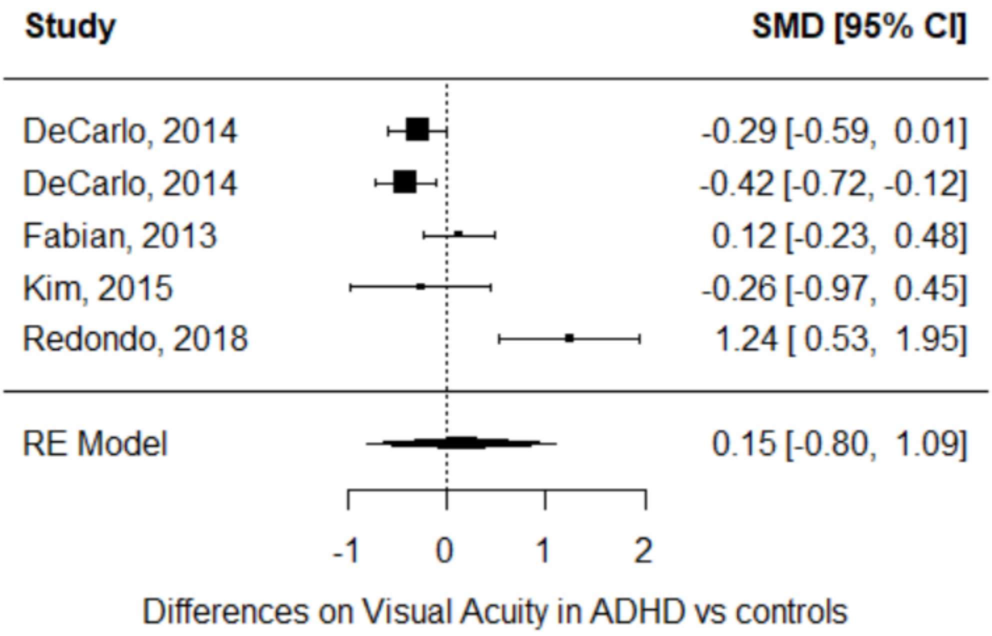


*RE: Random Effects; CI: Confidence Interval; SMD: Standardized Mean Difference*

**Test for Heterogeneity:** Q(df = 4) = 20.7980, p-val = 0.0003

*Figure S38. Funnel plots (a. no trim and fill analysis; b. trim and fill analysis excluding DeCarlo 2014.2, c. trim and fill analysis excluding DeCarlo 2014.1)*

**Correlation Test for Funnel Plot Asymmetry:** Kendall's tau = 0.6000, p = 0.2333

a.

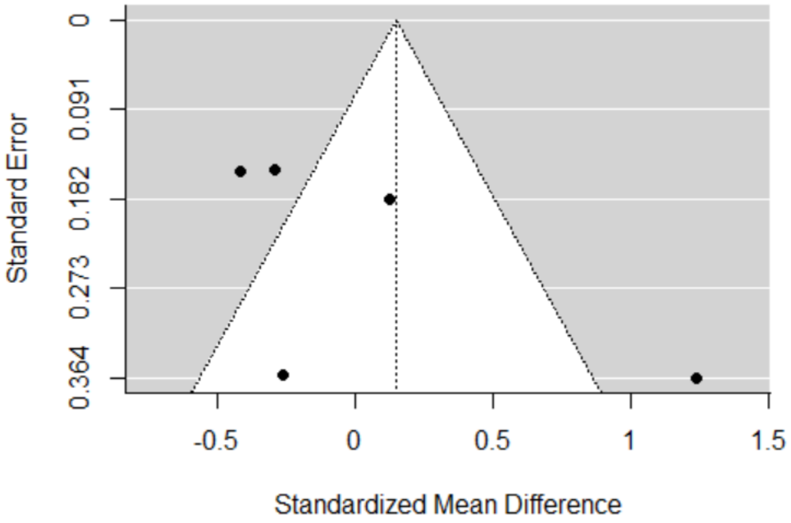


b.

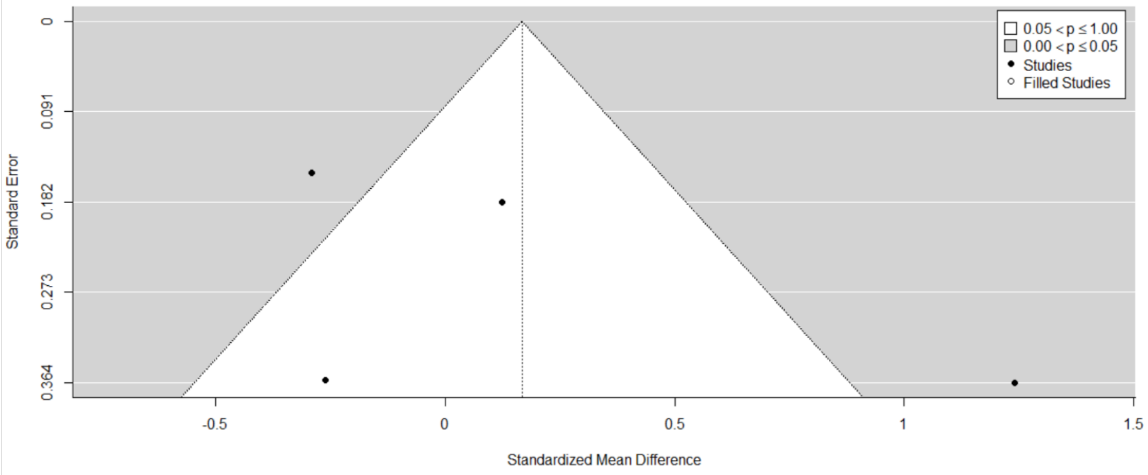


c.

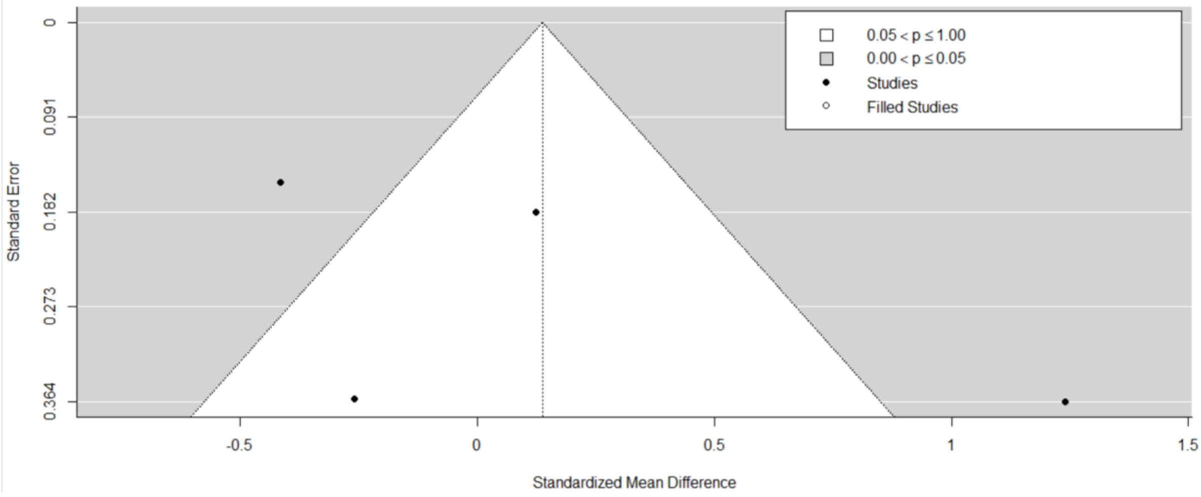

**Supplement 11: Results, forest and funnel plots for studies investigating differences on self-reported vision problems**

*Table S21. Description of studies*

| **Included in the meta-analysis** | |
|---|---|
| **Author, year** | **Hedge's g [variance]** |
| Farrar, 2001 | 0.9816 [0.1055] |
| Kim, 2014a | 0.2353 [0.0671] |
| Kim, 2014c.1 | 0.339 [0.0676] |
| Kim, 2014c.2 | 0.5012 [0.0688] |
| Kim, 2014c.3 | 0.2675 [0.0673] |
| Kim, 2014c.4 | 0.5578 [0.0693] |
| Kim, 2014c.5 | 0.914 [0.0736] |
| Kim, 2014c.6 | 0.4796 [0.0686] |
| Kim, 2014c.7 | 0.464 [0.0685] |
| Kim, 2014c.8 | 0.6248 [0.0699] |
| Kim, 2014c.9 | 0.8917 [0.0733] |
| Kim, 2014c.10 | 1.2314 [0.0793] |
| Redondo, 2018 | 1.1371 [0.1291] |

*Figure S39. Forest plot*

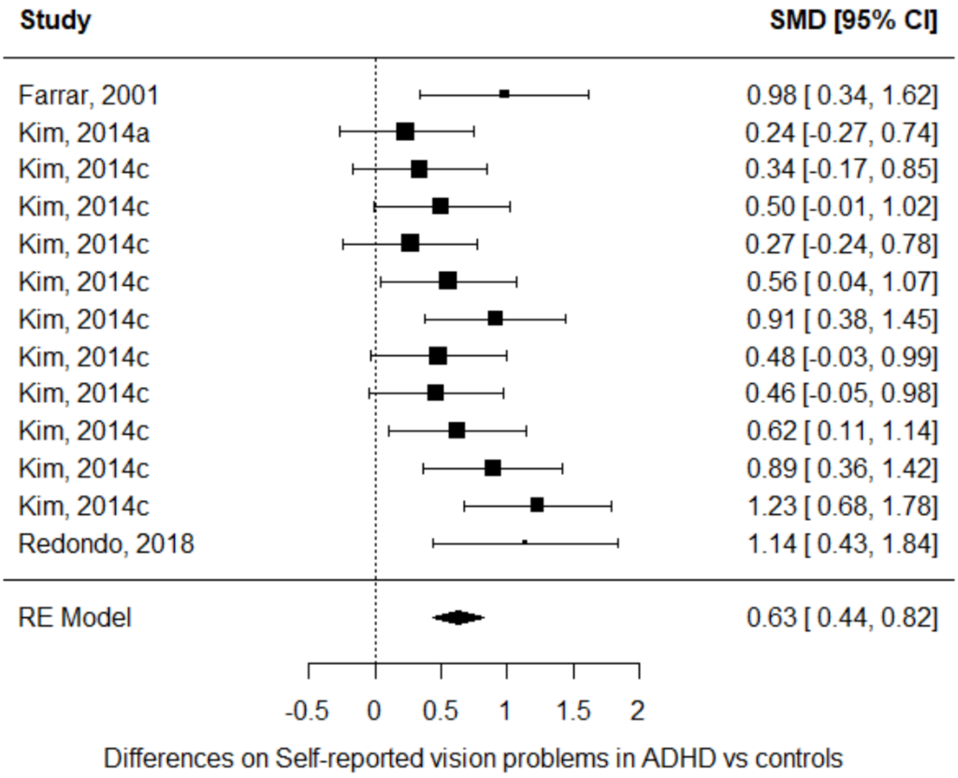


*RE: Random Effects; CI: Confidence Interval; SMD: Standardized Mean Difference*

**Test for Heterogeneity:** Q(df = 12) = 16.3265, p-val = 0.1767

***Figure S40. Funnel plots (a. no trim and fill analysis; b. trim and fill analysis excluding largest effect sizes for Kim, 2014c, c. trim and fill analysis excluding smallest effect sizes for Kim, 2014c)***

**Rank Correlation Test for Funnel Plot Asymmetry:** Kendall's tau = 0.9487, p < .0001

a.

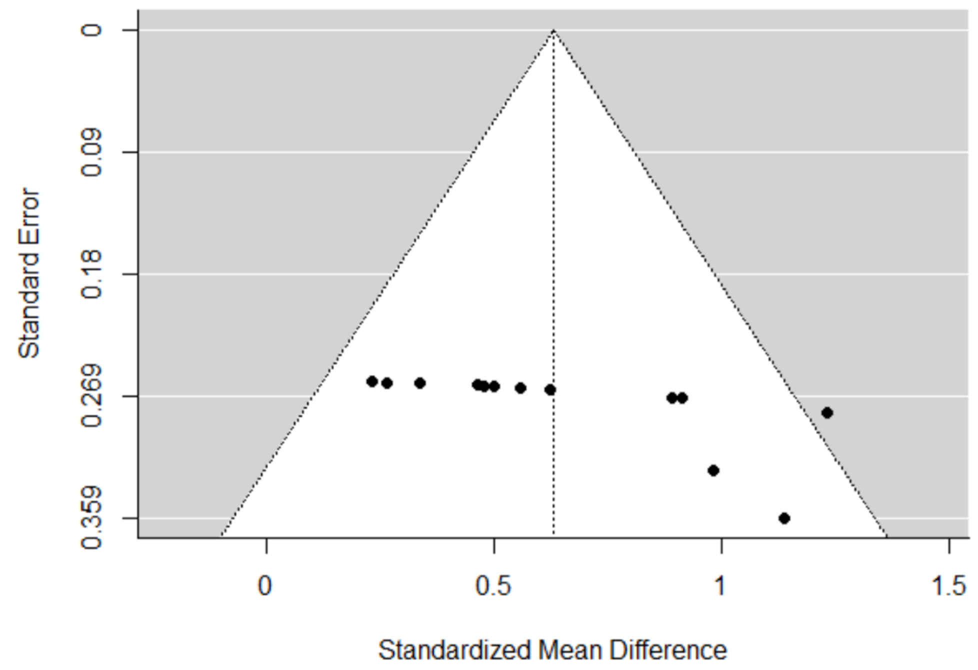


b.

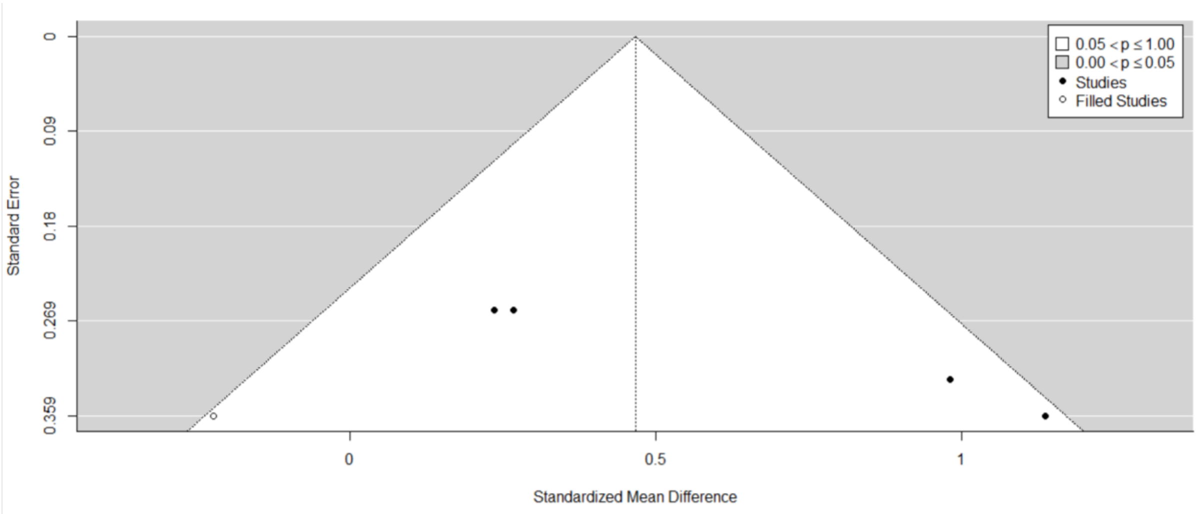

c.

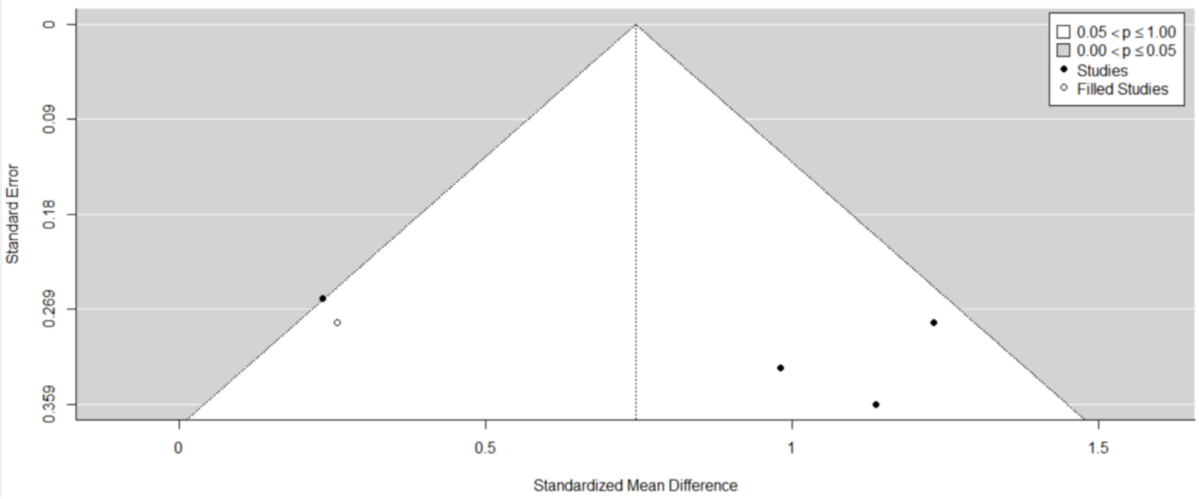

0.05 < p ≤ 1.00
0.00 < p ≤ 0.05
Studies
Filled Studies
Standard Error
0
0.09
0.18
0.269
0.359
0
0.5
1
1.5
Standardized Mean Difference
